# Synthesis of Bioactive 2-(Arylamino)thiazolo[5,4-*f*]-quinazolin-9-ones via the Hügershoff Reaction or Cu- Catalyzed Intramolecular C-S Bond Formation

**DOI:** 10.3390/molecules21060794

**Published:** 2016-06-18

**Authors:** Damien Hédou, Carole Dubouilh-Benard, Nadège Loaëc, Laurent Meijer, Corinne Fruit, Thierry Besson

**Affiliations:** 1 Normandie Univ, UNIROUEN, INSA Rouen, CNRS, COBRA, 76000 Rouen, France; d.hedou@laposte.net (D.H.); carole.dubouilh@univ-rouen.fr (C.D.-B.); corinne.fruit@univ-rouen.fr (C.F.); 2 Protein Phosphorylation & Human Disease group, Station Biologique, 29680 Roscoff, France; Nadege.Loaec@univ-brest.fr; 3 Manros Therapeutics, Centre de Perharidy, 29680 Roscoff, France; meijer@manros-therapeutics.com

**Keywords:** Hügershoff reaction, thiazolo[5,4-*f*]quinazolin-9(8*H*)-ones, microwave-assisted synthesis, protein kinases, CDK5, GSK-3, CLK1, CK1, DYRK1A

## Abstract

A library of thirty eight novel thiazolo[5,4-*f*]quinazolin-9(8*H*)-one derivatives (series **8**, **10**, **14** and **17**) was prepared via the Hügershoff reaction and a Cu catalyzed intramolecular C-S bond formation, helped by microwave-assisted technology when required. The efficient multistep synthesis of the key 6-amino-3-cyclopropylquinazolin-4(3*H*)-one (**3**) has been reinvestigated and performed on a multigram scale from the starting 5-nitroanthranilic acid. The inhibitory potency of the final products was evaluated against five kinases involved in Alzheimer’s disease and showed that some molecules of the **17** series described in this paper are particularly promising for the development of novel multi-target inhibitors of kinases.

## 1. Introduction

**Figure 1:**
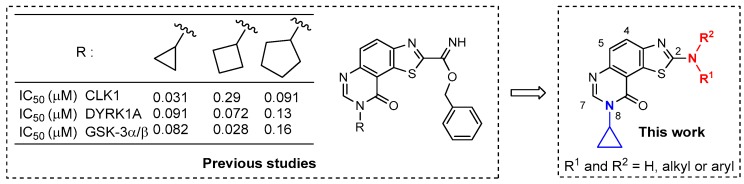
General formula of lead kinases inhibitors previously described [25] and new targeted molecules.

The occurrence and properties of the thiazole ring in various natural and synthetic products have been the interest of many research groups on account of its useful biological properties [1,2,3,4,5]. Since the 2000s our laboratory has been involved in a research program dealing with the preparation and pharmacological evaluation of original heterocyclic derivatives bearing a thiazole ring, mostly inspired by marine alkaloids [6,7,8,9,10]. In this context, our research group is mainly invested in the synthesis of C,N,S-containing bioactive molecules able to modulate the activity of deregulated kinases (CDK5, GSK-3, CLK1, CK1 and the dual-specificity kinase DYRK1A) involved to some extent in Alzheimer’s disease (AD) [11,12,13,14,15,16,17,18]. Consequently, the reactivity of 4,5-dichloro-1,2,3-dithiazolium chloride (Appel’s salt) [19] has been extensively studied for the synthesis of sulfur-nitrogen heteroarenes bearing a thiazole ring, substituted by a carbonitrile group at C-2. The chemistry of this versatile function was explored allowing access to novel imidate derivatives [11,12,13,14,15,16,17,18]. Among them some thiazolo[5,4-*f*]quinazolin-9(8*H*)-ones (Figure 1) have been revealed of particular interest in the design of multi-target-directed ligands (MTDLs), a new strategy for the development of powerful tools against neurodegenerative diseases [20,21,22,23,24].

**Figure 2:**
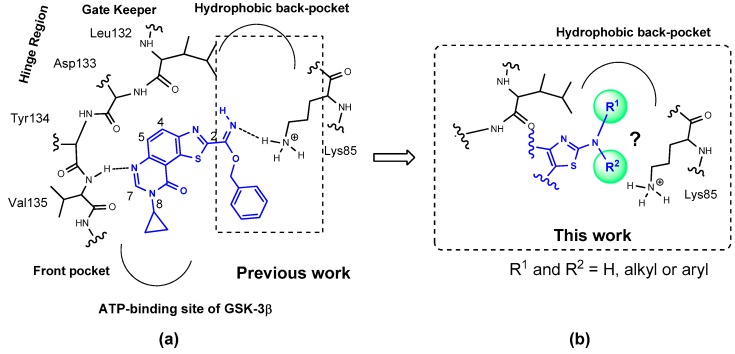
(**a**) Schematic representation of binding mode of one of the lead compounds in the ATP-binding site of GSK-3; (**b**) Description of the aminoalkyl and aryl substituents envisioned in position 2 of the thiazole with the aim of a better affinity and selectivity for target kinases (e.g., GSK-3).

The result of preliminary docking studies is schematized in Figure 2 and concerns the ATP-binding site of GSK-3β [11,12]. The nitrogen in position 6 of the thiazolo[5,4-*f*]quinazolin-9-one core seems to form a hydrogen bond with a NH-residue in the hinge segment, whilst the benzyl-carbimidate function linked to the thiazole moiety may form a polar interaction with the amino group of Lys85. In this hypothesis the bulky substituent may not fit into the hydrophobic backpocket of GSK-3β.

Considering that this region of the ATP-binding site of kinases can be used to gain affinity as well as selectivity, the synthetic route to novel C-2 substituted thiazolo[5,4-*f*]quinazolin-9-ones (Figure 1) bearing aminoalkyl and aminoaryl substituents at position C-2 of the thiazole moiety was investigated. In this study, the substituent present on position N-8 of the tricyclic structure was restrained to cyclopropyl, which has proved its efficiency in previous studies concerning this kinase group (see IC_50_ values in Figure 1) [25].

This paper describes the development of various methods allowing the preparation of a library of novel 2-aminoalkyl and 2-aminoarylthiazolo[5,4-*f*]quinazolin-9(8*H*)-ones for which interesting multi-target kinase inhibitory activities were expected. Following our previous strategies, the synthetic routes for this work were envisioned starting from commercially available anthranilic acids. A part of the chemistry described in this paper was achieved under microwave irradiation as a continuation of our global strategy consisting in the design of appropriate reagents and techniques offering operational, economic, and environmental benefits over conventional methods [26,27,28].

## 2. Results and Discussion

### 2.1. Chemistry

**Scheme 1:**
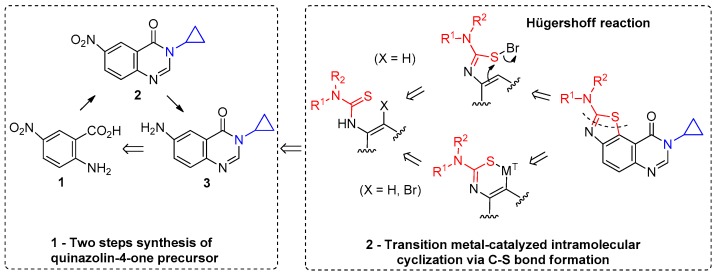
Envisioned retrosynthetic pathway for the synthesis of the target products, via Hügershoff reaction [29,30,31] or transition metal-catalyzed intramolecular C-S bond formation [32,33,34,35,36].

The target molecules were thiazolo[5,4-*f*]quinazolin-9(8*H*)-ones substituted in position N-8 by a cyclopropyl chain (Figure 1). The envisioned synthetic route is depicted in Scheme 1. The key intermediate 6-amino-3-cyclopropylquinazolin-4(3*H*)-one (**3**) was obtained from the corresponding nitro derivative **2**, itself obtained from 5-nitroanthranilic acid (**1**). The second part of the synthesis consisted in transforming the amine function into its thiourea analogue which can be cyclized into a thiazole ring by various methods. The first planned strategy uses the Hügershoff reaction, a bromine-mediated cyclization process involving electrophilic addition to the thiocarbonyl of the thiourea to afford a transient intermediate (e.g., by the seminal Br_2_ [29,30] or its recent equivalent such as benzyltrimethylammonium bromide [31]), which is then attacked by the π system of the aromatic ring, followed by rapid formation of thiazole ring. The second route imagined concerns a metal catalyzed intramolecular C-S bond formation [32,33,34,35,36] previously described for the synthesis of variously 2-substituted benzothiazoles from thiobenzanilides (Scheme 1).

#### 2.1.1. Synthesis of the Key 6-Amino-3-cyclopropylquinazolin-4(3*H*)-one (**3**)

To obtain an efficient route to the key 6-amino-3-cyclopropylquinazolin-4(3*H*)-one (**3**), the multistep strategy described in our previous works [11,12] was transformed into a convenient one-pot sequential process which was perfectly controlled using microwave heating at atmospheric pressure. Experimentally, the sequential MCR procedure consisted in irradiating of the starting 5-nitroanthranilic acid (**1**) and DMFDMA (2.5 equiv) in DMF for 15 min at 100 °C. After evaporation of the basic solvent (DMF), cyclopropylamine (1.1 equiv) and acetic acid were immediately added in the reaction flask and heated under microwaves at 100 °C for a further 15 min.

**Scheme 2:**
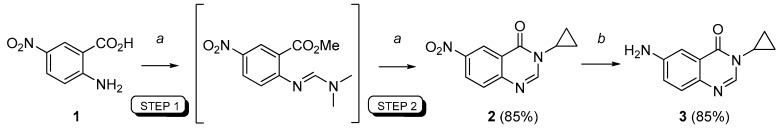
Sequential MCR procedure for convenient synthesis of 6-amino-3-cyclopropylquinazolin-4(3*H*)-one (**3**) from **1**. *Reagents and conditions:* (*a*) Step 1: DMFDMA (2.5 equiv), DMF, 100 °C (µw), 15 min; Step 2: Cyclopropylamine (1.1 eq), AcOH, 100 °C (µw), 15 min; 85%; (*b*) HCO_2_NH_4_ (5.0 equiv), Pd/C (10%), EtOH, 85 °C (μw), 15 min; 85%.

As depicted in Scheme 2 condensation of cyclopropylamine with the intermediate carbimidate gave 3-cyclopropyl-6-nitroquinazolin-4(3*H*)-one (**2**, 85% yield) which was reduced by treatment with ammonium formate in the presence of a catalytic amount of 10% palladium charcoal to afford the strategic 6-amino-3-cyclopropylquinazolin-4(3*H*)-one (**3**) in very good yield (85%). Some comments concerning the microwave procedure as well as the technical and practical aspects are warranted. In the case of the microwave-assisted synthesis, DMF and acetic acid present the advantage of having good dielectric properties, thus facilitating an efficient heating of the reaction mixture [37,38,39]. A reactor able to work at atmospheric pressure had some advantages, such as the possibility of easier scale-up and the use of common laboratory glassware. In the synthetic pathway steps described in this paper, an irradiation power at 900 W was enough to efficiently reach the programmed temperature with a short time ramp (2 min, not added to the reaction time indicated in schemes).

#### 2.1.2. Synthesis of 2-Aminoarylthiazolo[5,4-*f*]quinazolin-9-ones via Hügershoff Reaction

**Scheme 3:**
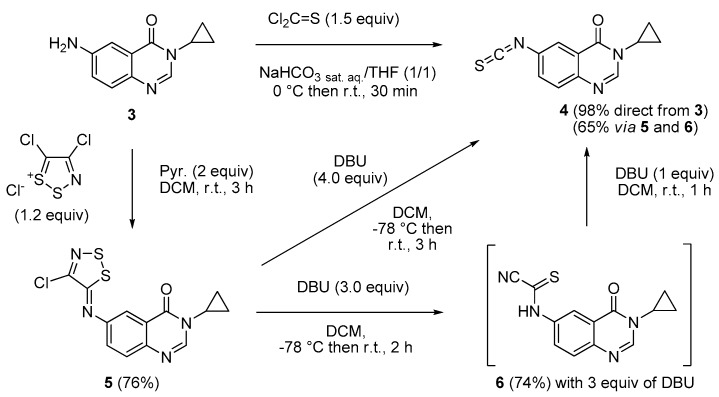
Synthetic routes for access to the key isothiocyanate **4**.

The seminal work of Hügershoff described the cyclization of thioureas with liquid bromine in chloroform to form the corresponding 2-aminobenzothiazoles [29,30]. According to these facts, the key isothiocyanate intermediate was needed as further precursor of substituted thioureas and their corresponding thiazoles. As described in Scheme 3, isothiocyanate **4** was obtained in nearly quantitative yield (98%) by treatment of amine **3** in the presence of thiophosgene in a mixture of tetrahydrofuran (THF) and a saturated aqueous solution of NaHCO_3_. The less toxic 1,1′-thiocarbonyl-diimidazole (TCDI) was found to be less effective.

According to previous works, the chemistry of 4,5-dichloro-1,2,3-dithiazolium chloride (Appel’s salt) allowed the safer synthesis of the attempted isothiocyanate precursor **4** [40,41]. Treatment of amine **3** with Appel’s salt in dichloromethane (DCM), in the presence of pyridine gave the corresponding imino-1,2,3-dithiazole derivative **5** which was stirred in DCM in the presence of 4 equiv of DBU [40], at −78 °C and then at room temperature, to afford **4** in a quite good yield (65%). Despite a low yield compared to the procedure using thiophosgene, this alternative synthesis has the advantage of being less toxic and safer than the former one. It can be noticed that treating **3** with 3 equiv of DBU led to the unique formation of the intermediate cyanothioformamide **6** (74%) [40]. The addition of 1 equiv. of DBU to **6** and stirring at room temperature for 1 h produced isothiocyanate **4** in a 88% yield (Scheme 3).

**Scheme 4:**
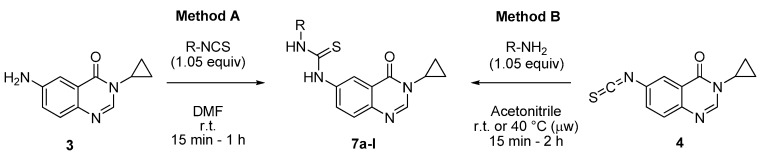
Synthesis of isothiocyanates **7a**–**l** from amines **3** or from isothiocyanates **4**.

Two methods were tested for the synthesis of the expected thioureas **7**, as depicted in Scheme 4 and Table 1. The first one depended of the availability of some isothiocyanates from commercial sources. It involved condensation of amine **3** with various aryl isothiocyanates to give compounds **7** (method A). In other cases, isothiocyanate **4** was treated with aniline derivatives to give **4** (method B). It should be noticed that in the case of less nucleophilic aromatic amines (e.g., 4-trifluoro-methylaniline and 3-aminopyridine), heating at 40 °C under microwave irradiation was required for access to the corresponding thioureas **7d** and **7g**.

**Scheme 5:**
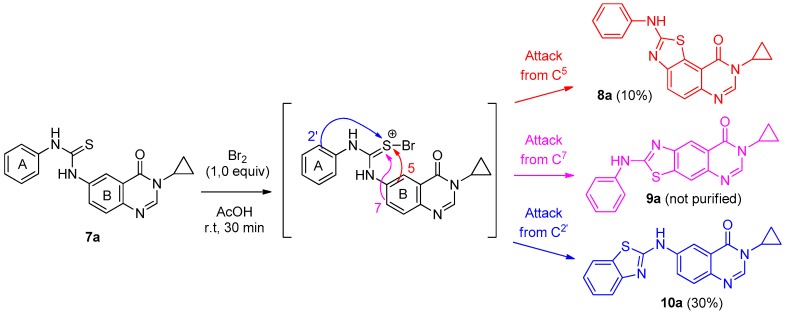
Hügershoff cyclization mechanism applied to thiourea **7a** and compounds **8a**, **9a** and **10a** identified.

With thioureas **7a**–**7l** in hand, the conditions of the Hügershoff reaction were examined using the *N*-phenylthiourea **7a**, with various oxidizing agents and solvents. The best results were obtained when **7a** was treated with 1.0 equiv of Br_2_ in acetic acid at room temperature (Scheme 5). However, although cyclization seemed effective, it was not regioselective. In the case of the *N*-phenyl derivative, the process resulted in a mixture of three isomeric compounds. NMR analysis demonstrated that the minor derivatives were the two tricyclic isomers **8a** and **9a**, whilst the major product was the benzothiazole derivative **10a** resulting from the attack of the benzene ring (A in Scheme 5) on the electrophilic sulfur atom, after oxidation of the thiourea with bromide. This result demonstrated that the benzene group (A) of the starting diarylthiourea **7a** possessed the most electron-rich *ortho*-carbon, compared to the quinazolin-4-one ring (B in Scheme 5). 

**Scheme 6:**
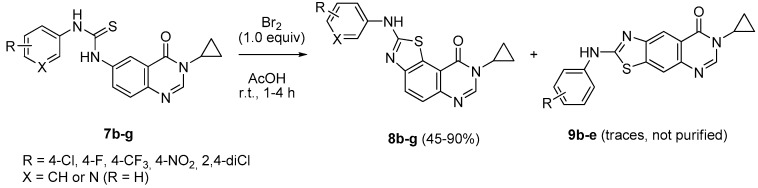
Synthesis of 8-cyclopropyl-2-(arylamino)thiazolo[5,4-*f*]quinazolin-9(8*H*)-ones **8b–f** and their pyrido analog **8g**.

Furthermore, analysis of isomers **8a** and **9a** confirmed that the most abundant was the angular derivative **8a** which was purified in poor yield (10%) whilst traces of **9a** did not allow its complete purification. Although the compound **10a** was isolated in a low yield (30%), the quantities obtained were sufficient for the biological experiments. Taking all these facts into account, the experimental conditions described above were extended to series **7b**–**7g** in which electron-deficient phenyl substituents are present in ring A of the thiourea derivatives (Scheme 6). Results are given in Table 2 where it was observed that isomers **9b**–**e** were apparently formed (per NMR analysis of crude reaction mixtures) without being purified. It was noted that compound **8b** was not separated from **9b**, whatever chromatographic methods were used.

**Scheme 7:**
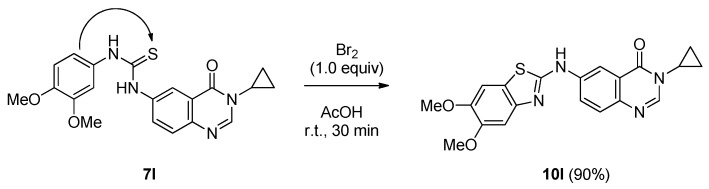
Hügershoff conditions applied to **7l** giving the corresponding 2-substituted benzothiazole **10l** selectively.

The Hügershoff conditions were also applied to a thiourea possessing highly electron-rich phenyl substituents (e.g., 3,4-diOMe). Compound **7l** was stirred for 30 min at room temperature to give unambiguously the corresponding benzothiazole **10l** (90% yield) via the regioselective cyclization of the 3,4-dimethoxyphenylthiourea into the thiazole ring (Scheme 7). At this stage of the study, a small library of 2-(arylamino)-8-cyclopropylthiazolo[5,4-*f*]quinazolin-9(8*H*)-ones **8a**, **8c**–**g** was prepared accompanied by the two 2-(benzo[*d*]thiazol-2-ylamino)-8-cyclopropylthiazolo[5,4-*f*]quinazolin-9(8*H*)-one derivatives **10a** and **10l**.

The results of this oxidative cyclization of thioureas depend of the substituents present on the aryl groups with a low tolerance. To overcome this drawback a metal catalyzed C-H functionalization/intramolecular C-S bond formation was envisioned, inspired by the work of Doi *et al.* on the synthesis of variously 2-substituted benzothiazoles from thiobenzanilides [32,33].

#### 2.1.3. Synthesis of 2-Aminoaryl- and 2-Aminoalkylthiazolo[5,4-*f*]quinazolin-9-ones via Metal Catalyzed C-S Bond Formation

According to the conditions described by Doi *et al.* [32,33], thiourea **7a** was stirred in the presence of palladium dichloride (PdCl_2_), copper iodide (CuI) and tetrabutylmammonim bromide (TBAB) in a DMSO/NMP (1:1, *v*/*v*) mixture as solvent. After 2 h of heating at 120 °C, all the starting material **7a** disappeared and workup revealed a mixture of three products. Analyses allowed us to identify **8a**, **9a** and **10a** (see Scheme 5), demonstrating a lack of selectivity under such conditions.

Needing regioselectivity in the cyclization process, we focused our attention on the work described ten years ago by Batey and Evindar on the conversion of *ortho*-bromoanilides or *orth*o-bromothioanilides into the corresponding benzoxazoles or benzothiazoles via intramolecular C-O or C-S bond formation [34,35,36]. Optimal conditions for cyclization used a catalyst combination of CuI (5 mol %) and 1.10-phenanthroline (10 mol %) in the presence of Cs_2_CO_3_ (2.0 equiv) in dimethoxyethane (DME).

**Scheme 8:**
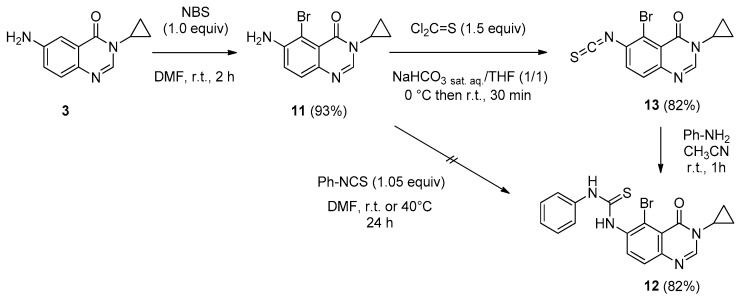
Synthesis of thiourea **12** via condensation of aniline with isothiocyanate **13**.

In a first approach, it was decided to prepare the phenylthiourea **12** for testing the metal-catalyzed cyclization conditions. Our study started from the *ortho*-brominated derivative of **3** which was easily obtained by stirring the starting amine **3** with *N*-bromosuccinimide (NBS, 1.0 equiv) in DMF at room temperature for 2 h. 6-Amino-5-bromo-3-cyclopropylquinazolin-4(3*H*)-one (**11**) was obtained in a high yield of 93% (Scheme 8).

The key *ortho*-brominated thiourea **12** was synthesized according to the aforementioned strategy used for the synthesis of **7a**. Unfortunately condensation of amine **11** with phenyl isothiocyanate was not fructuous, certainly due to the steric hindrance of the bromide associated with the low nucleophilicity of the aromatic amino group. To overcome this drawback the 5-bromo-6-isothiocyanatoquinazolin-4(3*H*)-one (**13**) was readily prepared (82% yield) by treatment of **11** with thiophosgene in a mixture of THF/NaHCO_3_ sat. aq_._ (1:1, *v*/*v*), as previously described. The phenyl-thiourea **12** was obtained by condensation of aniline with isothiocyanate **13** in acetonitrile at room temperature for 1 h (82% yield) (Scheme 8).

Experimental conditions for metal-catalyzed cyclization of thiourea **12** were adapted according to Batey *et al.* [34,35,36]: CuI 10 mol %, 1,10-phenantroline (20 mol %) and Cs_2_ CO_3_ (2.0 equiv) as a base. Instead of 24 h of conventional heating conditions, only 1 h of microwave-assisted heating (80 °C) allowed complete conversion of the starting material **12** (according to NMR analysis) into the cyclized product **8a** (Table 3, entry 1).

**Scheme 9:**
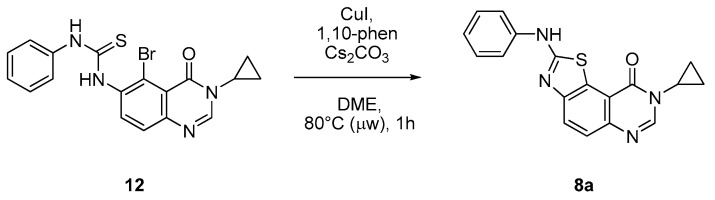
General description of the intramolecular metal-catalyzed cyclization of **12**.

Encouraged by this good result, a short optimization study was engaged in order to extend the best experimental conditions to a larger scope of products (Scheme 9, Table 3). Various conditions were screened, changing the quantities of copper catalyst, ligand and base. It was quickly confirmed that 10 mol % of CuI was necessary for a successful cyclization, under ligand-free conditions and in the presence of 2.0 equiv of Cs_2_ CO_3_ as a base (Table 3, entry 2).

**Scheme 10:**
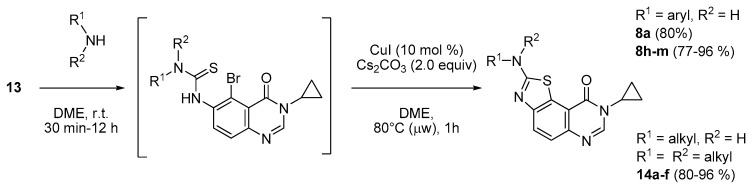
One-pot sequential CuI catalyzed intramolecular cyclization of intermediates thioureas (from **13**) for the synthesis of series **8** and **14**.

Once cyclization conditions were optimized, a sequential one-pot version of the transformation of isothiocyanate **13** into the tricyclic arene **8** series was considered. This approach required the complete conversion of the starting material **13** into the corresponding thiourea intermediate (in square brackets in Scheme 10) before addition of other reagents (CuI and Cs_2_CO_3_) and microwave-assisted cyclization by heating for 1 h. Depending on the nature of aniline, the conversion time can vary from 30 min (electron-rich compounds e.g., 4-OMe, 3,4-diOMe or 4-NMe_2_ anilines) to an overnight stirring (12 h) as observed for the deactivated aniline substituted by a sulfonamide function (see compound **8m** in Scheme 10 and Table 4). Because electron-poor anilines required several hours for completion of the first part of the reaction, the Hügershoff reaction was found to be more efficient in these cases (products **8d**–**f**).

The methodology described for the anilines **8h**–**m** was then extended to various alkylamines chosen for their frequency in various bioactive molecules. All the thiourea intermediates were formed *in situ* after 30 min of stirring. Microwave-assisted heating at 80 °C for 1 h complete the sequence to afford the expected cyclized compounds (series **14a**–**f**) in very good yields (80%–96%, see Table 5).

**Scheme 11:**
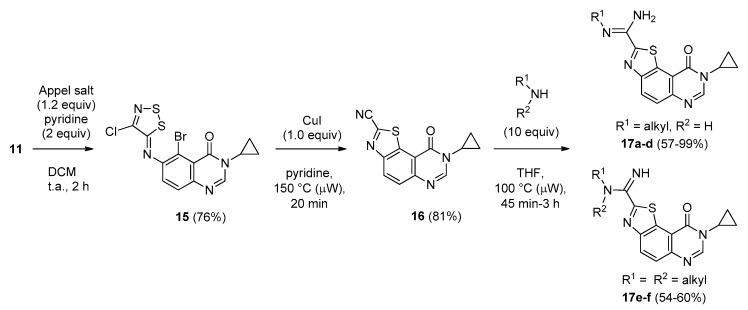
Synthesis of carboximidamides **17a**–**f** from brominated amine **11**.

With the intent to compare the biological results of the aforementioned products **14a**–**f** with their imidate analogues, the synthesis of a small library of 9-oxo-(8*H*)thiazolo[5,4-*f*]quinazoline-2-carboximidamides was undertaken via the synthesis of the key 8-cyclopropyl-9-oxo-8,9-dihydrothiazolo[5,4-*f*]quinazoline-2-carbonitrile (**16** in Scheme 11). Compound **16** was obtained by reaction of **11** with 4,5-dichloro-1,2,3-dithiazolium chloride (Appel’s salt) following by a copper-mediated cyclization of the intermediate imine **15** (Scheme 11). As described in previous works [16,17] the versatile carbonitrile function in position 2 of the thiazole ring may allow the synthesis of various amidine or imidate derivatives. A new set of six novel carboximidamides **17a**–**f** was prepared by stirring **16** with the appropriate amines (10 equiv) under microwave-assisted heating at 100 °C. The chemical structures and yields obtained for the synthesis of the series of compounds **17a**–**f** prepared are described in Table 6.

These studies showed that the synthetic access to fourteen novel 2-arylamino-substituted thiazolo[5,4-*f*]quinazolin-9(8*H*)-ones can be performed from *ortho*-brominated thioureas via a regio-selective intramolecular C-S coupling reaction, catalyzed by copper iodide. Optimization of experimental conditions served to develop a one-pot sequential process helped by microwave assisted heating. This allowed the convenient synthesis of new thiazoloquinazolin-9(8*H*)-ones substituted in position 2 by various aromatic and aliphatic amine groups for which biological activity was expected.

### 2.2. Biological Studies

Products of series **7a**–**l**, **8a**–**m**, **10a** and **10l**, **14a**–**f** and **17a**–**f** were tested in five different *in vitro* kinase assays (CDK5/p25 (cyclin-dependent kinase), CK1δ/ε (casein kinase 1), GSK-3α/β (glycogen synthase kinase 3), DYRK1A (dual-specificity tyrosine phosphorylation regulated kinase) and CLK1 (cdc2-like kinase 1) to evaluate their inhibition potency [43,44,45,46,47,48]. All compounds were first tested at a final concentration of 10 µM. Compounds showing less than 50% inhibition were considered as inactive (IC_50_ > 10 µM). Compounds displaying more than 50% inhibition at 10 µM were next tested over a wide range of concentrations (usually 0.01 to 10 µM), and IC_50_ values were determined from the dose-response curves (Sigma-Plot). Harmine (Table 7) is a β-carboline alkaloid known to be a potent inhibitor of DYRK1A [49]. It was also tested as positive control and its IC_50_ values were compared to those obtained for the compounds under study.

Results given in Table 7 demonstrate that none of the tricyclic derivatives prepared in series **7**, **8**, **10** and **14** showed significant inhibitory activity against the set of five kinases tested. Other derivatives substituted in position 2 of the thiazole by an aminoaryl or aminoalkyl group were completely inactive against all kinases.

On a general aspect, most interesting biological activity of the tested compounds was oriented towards one kinase of the initial panel: CLK1. This latter is one of the four isoforms (CLK1-4) of the cdc2-like kinase family. In humans, the highest levels of CLK1 expression were found in the brain. It was described that inhibitors CLK inhibitors may alter the splicing of microtubule-associated protein tau implicated in Alzheimer’s disease and Parkinson’s disease [48]. Three molecules of series **8** (**8a**, **8g** and **8j**), compound **10a** and products **14a**, **14d** and **14e**, have shown micromolar activity (1.3 μM < IC_50_ < 8.1 μM) against the CLK1 kinase.

In series **8a–m** and in all the compounds prepared in series **7**, **8**, **10** and **14**, only one compound inhibits in the micromolar range (1.3 μM < IC_50_ < 7.3 μM) CLK1, DYRK1A and GSK3. This compound is the 8-cyclopropyl-2-[(4-(dimethylamino)phenyl]amino-thiazolo [5,4-*f*]quinazolin-9(8*H*)-one (**8j**) which possesses a tertiary amine in the *ortho*-position of the aminoarene substituent.

Undoubtedly, the most active molecules prepared in this study were members of the **17a**–**f** seriesin which the final carboximidamide functions were obtained after attack of various amines on the initial carbonitrile function. Most of these compounds showed micromolar or submicromolar activities against CLK1 (0.38 μM < IC_50_ < 0.61 μM), DYRK1A (0.14 μM < IC_50_ < 0.82 μM) and GSK-3α/β (0.23 μM < IC_50_ < 0.43 μM).

The two derivatives **17e** and **17f** are the most active products of this study, their interesting IC_50_ values being slightly better for **17e** than for **17f**. These two isosters are also bioisosters, considering the results given in Table 7.

Comparing the results of this study with our previous works, it seems rather obvious that having such molecular scaffolds with submicromolar affinities for various kinases is related to the presence of carboximidamide or carboximidate functions that result from the substitution of a carbonitrile group itself present in position 2 of the thiazole.

Because drugs focusing their activity against a single target may generate low benefits, recent studies are encouraged in the direction of multi-targeting strategies. In the present case, the novel structures studied are not the most valuable for the further discovery of multi-kinases inhibitors. Developing molecules showing submicromolar affinities on a panel of two or three kinases needs to leave a substituted carbon on position 2 of the thiazole part of these scaffolds.

## 3. Materials and Methods

### 3.1. General Information

Materials were obtained commercially and used without further purification. All reactions were monitored by thin-layer chromatography with silica gel 60 F254 precoated aluminium plates (0.25 mm). Visualization was performed with UV light at wavelengths of 254 and 312 nm. Purifications were conducted with a flash column chromatography system equipped with a dual UV/Vis spectrophotometer (200–600 nm), a fraction collector (176 tubes), a dual piston pump (1 to 200 mL/min, *P*_max_ = 15 bar), which allowed quaternary gradients, and an additional inlet for air purge. The melting points of solid compounds were measured with a STUART Melting Point SMP3 instrument (Bibby Scientific Ltd., Roissy, France) with a precision of 1.5 °C. IR spectra were recorded with a Spectrum 100 Series FTIR spectrometer (PerkinElmer, Villebon S/Yvette, France). Liquids and solids were investigated with a single-reflection attenuated total reflectance (ATR) accessory; the absorption bands are given in cm^−1^. The NMR spectra (^1^H and ^13^C) were acquired at 295 K using a WP 300 spectrometer AVANCE 300 MHz spectrometer (Bruker, Wissembourg, France) at 300 and 75.4 MHz, using TMS as an internal standard. Coupling constants *J* are in Hz, and chemical shifts are given in ppm. Signals in ^13^C spectra were assigned based on the result of 13C DEPT135 experiments (see Supplementary Materials). Mass spectrometry was performed by the Mass Spectrometry Laboratory of the University of Rouen. The mass spectra [ESI, EI, and field desorption (FD)] were recorded with a LCP 1er XR spectrometer (WATERS, Guyancourt, France). Microwave experiments were conducted in a commercial microwave reactor especially designed for synthetic chemistry. Start S™ (Milestone S.r.l., Bergamo, Italy) is a multi-mode cavity with a microwave power delivery system ranging from 0 to 1200 W. The temperatures of the reactions were mainly monitored via contact-less infrared pyrometer which was calibrated in control experiments with a fibre-optic contact thermometer protected in a Teflon coated ceramic well inserted directly in the reaction mixture. Open vessel experiments were carried out in a 50–250 mL round bottom flask fitted with a reflux condenser. The vessel contents were stirred by means of an adjustable rotating magnetic plate located below the floor of the microwave cavity and a Teflon-coated magnetic stir bar inside the vessel. Temperature and power profiles were monitored in both cases through the EASY-Control software provided by the manufacturer (Milestone S.r.l., Bergamo, Italy). The times indicated in the various protocols are the times measured when the mixtures reached the programmed temperature after a ramp period of 2 min.

### 3.2. Chemistry

*3-Cyclopropyl-6-nitroquinazolin-4(3H)-one* (**2**). A stirred suspension of 5-nitroanthranilic acid **1** (1.5 mmol, 1 equiv.) in DMFDMA (500 µL, 2.5 equiv.) and DMF (1.5 mL) was heated at 100 °C for 15 min under microwave (after a ramp period of 2 min at 900 W). Solvents were removed *in* *vacuo* and cyclopropylamine (1.65 mmol, 1.1 equiv.) was added followed with AcOH (1.5 mL). The mixture was irradiated at 100 °C (900 W) for 15 min (ramp: 2 min). Evaporation of the solvent gave a crude product which was purified by column chromatography using petroleum ether/methylene chloride (100:0 to 0:100, *v/v*) as eluent. Compound **2** was isolated as a light yellow solid (295 mg, 85%), mp. 174–176 °C; ^1^H-NMR (DMSO-d_6_) δ 8.82 (d, *J* = 2.7 Hz, 1H, H_5_), 8.54 (dd, *J* = 9.0, 2.7 Hz, 1H, H_7_), 8.45 (s, 1H, H_2_), 7.86 (d, *J* = 9.0 Hz, 1H, H_8_), 3.34–3.25 (m, 1H, NCH), 1.08–0.99 (m, 4H, CH);^13^C-NMR (DMSO-d_6_) δ 160.6, 151.6, 151.3, 145.1, 128.9, 128.1, 121.9, 121.4, 29.5, 5.9; ν_max_ 3356, 3102, 1675, 1600, 1512, 1300, 1275, 936, 855, 752 cm^−1^; HRMS calcd for C_11_H_10_N_3_O_3_ [M + H]^+^ 232.0722 found 232.0719.

*6-Amino-3-cyclopropylquinazolin-4(3H)-one* (**3**). A stirred mixture of 6-nitro-3-cyclopropyl-quinazolin-4(3H)-one **2** (15 mmol, 1.0 equiv.), ammonium formate (5.0 equiv.) and a catalytic (10 mol %) amount of 10% palladium charcoal in ethanol (0.2 M solution) was heated under microwaves for 20 min (300 W, 85 °C). The reaction mixture was filtered through Celite^®^ and washed with hot ethanol. The solvent was removed in *vacuo* to give the crude product which was dissolved in ethyl acetate, washed with water, dried over MgSO_4_ and concentrated under reduced pressure to give the reduced compound as a pale yellow solid (2.6 g, 85%), mp. 172–174 °C; ^1^H-NMR (DMSO-*d*_6_) δ 7.94 (s, 1H, H_2_), 7.37 (d, *J* = 8.7 Hz, 1H, H_8_), 7.22 (d, *J* = 2.7 Hz, 1H, H_5_), 7.07 (dd, *J* = 8.7, 2.7 Hz, 1H, H_7_), 5.66 (br s, 2H, NH_2_), 3.20–3.16 (m, 1H, NCH), 1.03–1.01 (m, 2H, CH), 0.90 (m, 2H, CH); ^13^C-NMR (DMSO-*d*_6_) δ 161.3, 148.1, 142.7, 138.3, 127.9, 122.3, 122.0, 106.1, 28.9, 6.0; ν_max_ 3420, 3336, 3222, 1653, 1627, 1600, 1491, 1325, 830, 791 cm^−1^; HRMS calcd for C_11_H_12_N_3_O [M + H]^+^ 202.0980 found 202.0974.

*3-Cyclopropyl-6-isothiocyanatoquinazolin-4(3H)-one* (**4**). Method A: A solution of DBU (542 mg, 3.57 mmol, 3.0 equiv.) in methylene chloride (1.0 mL) was added drop wise to a stirred solution of 6-aminoquinazolin-4(3*H*)-one **5** (400 mg, 1.19 mmol) in methylene chloride (7 mL) maintained at −78 °C. The resulting mixture was warmed to room temperature and stirred for 2 h. One equivalent of DBU (181 mg, 1.19 mmol) was added and the black solution was stirred for 1 h. Evaporation of solvent gave a crude mixture which was adsorbed on Celite^®^ and purified by flash chromatography using ethyl acetate and methylene chloride (0/100 to 50/50, *v/v*) as eluent to furnish **4** as a white solid (188 mg, 65%). Method B: Thiophosgene (0.57 mL, 7.47 mmol, 1.5 equiv.) was added drop wise to a stirred solution of 6-aminoquinazolin-4(3*H*)-one **3** (1.00 g, 4.97 mmol) in a mixture of THF (5.0 mL) and a saturated solution of NaHCO_3_ (5.0 mL) maintained at 0 °C. The resulting mixture was warmed to room temperature and stirred for 30 min. On completion, the reaction mixture was diluted with water (25 mL) and ethyl acetate (25 mL). The aqueous layer was extracted with ethyl acetate and the combined organic layers were washed with water and brine. Evaporation of solvent gave a crude mixture which was adsorbed on Celite^®^ and purified by flash chromatography using ethyl acetate and methylene chloride (0/100 to 50/50, *v/v*) as eluent to furnish **4** as a colorless solid (1.18 g, 98%); mp. 146–148 °C; ^1^H-NMR (DMSO-*d*_6_) δ 8.31 (s, 1H, H_2_), 8.04 (d, *J* = 2.2 Hz, 1H, H_5_), 7.82 (dd, *J* = 8.6, 2.2 Hz, 1H, H_7_), 7.70 (d, *J* = 8.6 Hz, 1H, H_8_), 3.28–3.21 (m, 1H, NCH), 1.08–0.97 (m, 4H, CH); ^13^C-NMR (CDCl_3_) δ 161.2, 147.4, 146.2, 137.8, 131.7, 130.6, 129.2, 123.3, 122.9, 29.6, 6.7; ν_max_ 2118, 1669, 1602, 833, 535 cm^−1^; HRMS calcd for C_12_H_10_N_3_OS [M + H]^+^ 244.0545 found 244.0537.

*6-[(4-Chloro-5H-1,2,3-dithiazol-5-yl)amino]-3-cyclopropylquinazolin-4(3H)-one* (**5**). A suspension of 6-amino-3-cyclopropylquinazolin-4(3*H*)-one (**3**, 300 mg, 1.49 mmol) and 4,5-dichloro-1,2,3-dithiazolium chloride (1.2 equiv.) in DCM (0.1 M solution) was stirred for 1 h at room temperature under an argon atmosphere. Pyridine (2.0 equiv.) was added and the mixture was stirred again for 2 h at room temperature. The resulting solution was concentrated under *vacuo* to give a crude residue which was purified by chromatography on silica gel with EtOAc/DCM (5/95 then 50/50, *v/v*) to give the expected iminodithiazole as a yellow solid (382 mg, 76%), mp. 190–192 °C; ^1^H-NMR (DMSO-*d*_6_) δ 8.29 (s, 1H, H_2_), 7.97 (d, *J* = 2.4 Hz, 1H, H_5_), 7.78 (d, *J* = 8.7 Hz, 1H, H_8_), 7.65 (dd, *J* = 8.7, 2.4 Hz, 1H, H_7_), 3.20–3.16 (m, 1H, NCH), 1.09–0.94 (m, 4H, CH); ^13^C-NMR (DMSO-*d*_6_) δ 161.0, 160.4, 149.0, 147.7, 147.0, 145.4, 129.1, 128.2, 122.3, 114.2, 29.3, 5.9; ν_max_ 3073, 1659, 1592, 1530, 1454, 1305, 951, 842, 581 cm^−1^; HRMS calcd for C_13_H_10_N_4_OS_2_Cl_2_ [M + H]^+^ 336.9985 found 336.9974.

*(3-Cyclopropyl-4-oxo-3,4-dihydroquinazolin-6-yl)carbamothioyl cyanide* (**6**). A solution of DBU (542 mg, 3.57 mmol, 3.0 equiv.) in methylene chloride (1.0 mL) was added drop wise to a stirred solution of iminodithiazole **5** (400 mg, 1.19 mmol) in methylene chloride (7.0 mL) maintained at −78 °C. The resulting mixture was warmed to room temperature and stirred for 2 h. Evaporation of solvent gave a crude mixture which was adsorbed on Celite^®^ and purified by flash chromatography using ethyl acetate/methylene chloride (0/100 to 50/50, *v/v*) as eluent to furnish **6** as an orange solid (250 mg, 74%), mp. 196–198 °C; ^1^H-NMR (DMSO-*d*_6_) δ 13.72 (br s, 1H, NH), 8.91 (d, *J* = 2.4 Hz, 1H, H_5_), 8.32 (s, 1H, H_2_), 8.16 (dd, *J* = 9.0, 2.7 Hz, 1H, H_7_), 7.76 (d, *J* = 9.0 Hz, H_8_), 3.20–3.16 (m, 1H, NCH), 1.09–0.94 (m, 4H, CH); ^13^C-NMR (DMSO-*d*_6_) δ 161.1, 160.8, 148.4, 146.0, 136.9, 128.7, 128.0, 121.4, 118.7, 114.0, 29.3, 5.9; ν_max_ 3519, 3430, 2944, 2055, 1641, 1601, 1487, 1389, 1256, 1193, 1021, 847, 739 cm^−1^; HRMS calcd for C_13_H_11_N_4_OS [M + H]^+^ 271.0654 found 271.0655.

*6-Amino-5-bromo-3-cyclopropylquinazolin-4(3H)-one* (**11**). To a stirred solution of 6-amino-3-cyclo-propylquinazolinone **3** (5.5 g, 27.3 mmol) was added NBS (4.9 g, 27.3 mmol, 1.0 equiv.) at room temperature. After 2 h of stirring at room temperature, the solvent was removed under *vacuum* and the crude residue was purified by chromatography on silica gel using EtOAc/DCM (20/80 to 50/50, *v/v*) as eluent to give the compound **11** as a colorless solid (7.1 g, 93%), mp. 172–174 °C; ^1^H-NMR (DMSO-*d*_6_) δ 8.03 (s, 1H, H_2_), 7.40 (d, *J* = 8.7 Hz, 1H, H_8_), 7.28 (d, *J* = 8.7 Hz, 1H, H_7_), 5.86 (br s, 2H, NH_2_), 3.18–3.13 (m, 1H, NCH), 1.03–0.99 (m, 2H, CH), 0.91–0.85 (m, 2H, CH); ^13^C-NMR (DMSO-*d*_6_) δ 159.6, 146.1, 144.0, 140.4, 127.4, 121.7, 119.5, 100.5, 29.4, 6.0; ν_max_ 3088, 3017, 2926, 2848, 1675, 1508, 1335, 1141, 855, 752, 699 cm^−1^; HRMS calcd for C_11_H_11_N_3_O^79^Br [M + H]^+^ 280.0085 found 280.0095 (100%), calcd for C_11_H_11_N_3_O^81^Br [M + H]^+^ 282.0065 found 282.0072 (93%).

*1-(5-Bromo-3-cyclopropyl-4-oxo-3,4-dihydroquinazolin-6-yl)-3-phenylthiourea* (**12**). A solution of isothiocyanate **13** (0.200 g, 0.62 mmol) and aniline (61 µL, 0.65 mmol, 1.05 equiv.) in acetonitrile (2.0 mL) was stirred at room temperature overnight. the solvent was removed under vacuum und the crude residue was adsorbed on Celite^®^ and purified by flash chromatography using ethyl acetate and methylene chloride (0/1 to 5/5, *v/v*) to furnish the expected thiourea **12** as a colorless solid (0.211 g, 82%), mp. 212–214 °C; ^1^H-NMR (DMSO-*d*_6_) δ 10.11 (s, 1H, NH), 9.47 (s, 1H, NH), 8.31 (s, 1H, H_2_), 7.92 (d, *J* = 8.7 Hz, 1H, H_8_), 7.62 (d, *J* = 8.7 Hz, 1H, H_7_), 7.57–7.55 (m, 2H, Ph), 7.39–7.34 (m, 2H, Ph), 7.19–7.14 (m, 1H, Ph), 3.22–3.17 (m, 1H, NCH), 1.04–0.92 (m, 4H, CH); ^13^C-NMR (DMSO-*d*_6_) δ 180.2, 159.6, 148.2, 147.8, 139.0, 137.9, 135.1, 128.6, 126.4, 124.8, 123.7, 119.6, 119.1, 29.2, 6.0; ν_max_ 3315, 2930, 1677, 1619, 1515, 1324, 1297, 897, 696, 501 cm^−1^; HRMS calcd for C_18_H_16_N_4_OS^79^Br [M + H]^+^ 415.0228 found 415.0233 (93%), for C_18_H_16_N_4_OS^81^Br [M + H]^+^ 417.0208 found 417.0197 (100%).

*5-Bromo-3-cyclopropyl-6-isothiocyanatoquinazolin-4(3H)-one* (**13**). According to the previous procedure described for compound **4**, 6-amino-5-bromoquinazolin-4(3*H*)-one (**11**, 1.00 g, 3.57 mmol) was stirred with thiophosgene (0.410 g, 5.35 mmol, 1.5 equiv.) in a mixture of THF (3.5 mL) and a saturated solution of NaHCO_3_ (3.5 mL) to furnish **13** as a colorless solid (0.940 g, 82%), mp. 210–212 °C; ^1^H-NMR (DMSO-*d*_6_) δ 8.36 (s, 1H, H_2_), 7.90 (d, *J* = 8.7 Hz, 1H, H_8_), 7.67 (d, *J* = 8.7 Hz, 1H, H_7_), 3.23–3.16 (m, 1H, NCH), 1.05–0.93 (m, 4H, CH); ^13^C-NMR (CDCl_3_) δ 159.9, 148.0, 147.6, 138.7, 132.1, 131.3, 128.3, 121.1, 120.0, 30.1, 6.7 (2C); ν_max_ 3061, 2921, 2122, 1688, 1588, 1454, 1314, 1259, 838 cm^−1^; HRMS calcd for C_12_H_9_N_3_OS^79^Br [M + H]^+^ 321.9650 found 321.9647, for C_12_H_9_N_3_OS^81^Br [M + H]^+^ 323.9629 found 323.9630.

*(Z)-5-Bromo-6-[(4-chloro-5H-1,2,3-dithiazol-5-ylidene)amino]-3-cyclopropylquinazolin-4(3H)-one* (**15**). A suspension of 6-amino-5-bromo-3-cyclopropylquinazolin-4(3*H*)-one (**11**, 2.0 g, 1.0 equiv.) and 4,5-dichloro-1,2,3-dithiazolium chloride (1.2 equiv.) in DCM (0.1 M solution) was stirred for 1 h at room temperature under an argon atmosphere. Pyridine (2.0 equiv.) was added and the mixture was stirred again for 2 h at room temperature. The resulting solution was concentrated under *vacuo* to give a crude residue which was purified by chromatography on silica gel with EtOAc/DCM (5/95 then 50/50, *v/v*) to give the desired iminodithiazole as an orange solid (2.3 g, 76%), mp. 204–206 °C; ^1^H-NMR (DMSO-*d*_6_) δ 8.33 (s, 1H, H_2_), 7.75 (d, *J* = 8.7 Hz, 1H, H_7_), 7.58 (d, *J* = 8.7 Hz, 1H, H_8_), 3.24–3.17 (m, 1H, NCH), 1.06–1.00 (m, 2H, CH), 0.97–0.94 (m, 2H, CH); ^13^C-NMR (DMSO-*d*_6_) δ 163.2, 159.6, 150.5, 147.9, 146.8, 145.8, 129.0, 124.6, 120.3, 111.3, 29.7, 6.0 (2C); ν_max_ 3003, 1862, 1675, 1593, 1452, 1325, 1293, 1148, 826, 759 cm^−1^; HRMS calcd for C_13_H_9_N_4_OS_2_^79^BrCl [M + H]^+^ 414.9090 found 414.9088.

*8-Cyclopropyl-9-oxo-8,9-dihydrothiazolo[5,4-f]quinazoline-2-carbonitrile* (**16**). A suspension of imine **15** (2.00 g, 1.0 equiv.), copper iodide (CuI, 2.0 equiv.) in pyridine (0.33 M solution) was irradiated under microwaves at 115 °C (power input: 300 W) for 20 min. After cooling, the mixture was diluted in EtOAc, washed with a saturated aqueous solution of sodium thiosulfate. The organic layer was dried over MgSO_4_ and the solvent was removed *in vacuo*. The crude residue was purified by column chromatography on silica gel with EtOAc/methylene chloride (0/100 to 20/80, *v/v*) as eluent to give the expected compound as a colorless solid (1.05 g, 81%), mp. 248–250 °C; ^1^H-NMR (DMSO-*d*_6_) δ 8.63 (d, *J* = 8.7 Hz, 1H, H_4_), 8.61 (s, 1H, H_2_), 7.99 (d, *J* = 9.0 Hz, 1H, H_5_), 3.42–3.36 (m, 1H, NCH), 1.13–1.09 (m, 4H, CH); ^13^C-NMR (DMSO-*d_6_*) δ 160.2, 150.3, 149.5, 148.2, 139.2, 131.6, 129.9, 127.8, 115.4, 113.5, 29.7, 5.9 (2C); ν_max_ 3067, 2233 (C≡N), 1664, 1579, 1441, 1353, 1303, 1222, 1038, 839, 692 cm^−1^; HRMS calcd for C_13_H_9_N_4_OS [M + H]^+^ 269.0497 found 269.0487.

#### 3.2.1. General Procedures for the Synthesis of *N*-(3-Cyclopropyl-4-oxo-3,4-dihydroquinazolin-6-yl)-*N′*-aryl or Alkylthioureas **7a**–**l** from 3-Cyclopropyl-6-isothiocyanatoquinazolin-4(3*H*)-one (**4**) or 6-Amino-3-cyclopropylquinazolin-4(3*H*)-one (**3**)

Method A: To a stirred solution of isothiocyanate **4** (250 mg, 1.03 mmol) in acetonitrile (3 mL) was added the appropriate amine (1.08 mmol, 1.05 equiv.) at room temperature. The resulting mixture was stirred at to room temperature for nucleophilic amines for 15 min to 2 h or under microwave at 40 °C for 1 h. On completion, the solvent was evaporated under *vacuum*. Trituration of crude mixture in diethyl ether (6 mL) followed by filtration furnished the expected thiourea derivatives **7a**, **c**–**d, g**, **j**–**l**.

Method B: To a stirred solution of 6-amino-3-cyclopropylquinazolinone (**3**, 250 mg, 1.24 mmol) in DMF (4 mL) was added the appropriate arylisothiocyanate (1.05 equiv.) at room temperature. The resulting mixture was stirred at room temperature for 15 min to 4 h. On completion, the solvent was evaporated under *vacuum*. Trituration of crude mixture in diethyl ether (8 mL) followed by filtration furnished the expected thiourea derivatives **7a**–**b**; **e**–**f**; **h**, **i**.

*N-(3-Cyclopropyl-4-oxo-3,4-dihydroquinazolin-6-yl)-N′-phenylthiourea* (**7a**): According to the method A (40 °C, 1 h) or the method B (r.t., 2 h), product **7a** was obtained as a colorless solid (291 mg, 84%) or (333 mg, 80%), mp > 265 °C; ^1^H-NMR (DMSO-*d*_6_) δ 10.09 (s, 1H, NH), 10.00 (s, 1H, NH), 8.25 (d, *J* = 1.8 Hz, 1H, H_5_), 8.21 (s, 1H, H_2_), 7.92 (dd, *J* = 8.7, 1.8 Hz, 1H, H_7_), 7.60 (d, *J* = 8.7 Hz, 1H, H_8_), 7.46 (d, *J* = 8.0 Hz, 2H, Ph), 7.34 (t, *J* = 8.0 Hz, 2H, Ph), 7.14 (t, *J* = 8.0 Hz, 1H, Ph), 3.26–3.19 (m, 1H, NCH), 1.03–0.92 (m, 4H, CH); ^13^C-NMR (DMSO-*d*_6_) δ 179.7, 161.0, 146.8, 144.1, 139.1, 138.3, 130.1, 128.5, 127.0, 124.5, 123.6, 121.1, 118.8, 29.1, 5.9; *ν*_max_ 3292, 3069, 2990, 1649, 1592, 1511, 1492, 1303, 1246, 1163, 1037, 821, 610, 580 cm^−1^; HRMS calcd for C_18_H_17_N_4_OS [M + H]^+^ 337.1123 found 337.1109.

*N-(4-Chlorophenyl)-N′-(3-cyclopropyl-4-oxo-3,4-dihydroquinazolin-6-yl)thiourea* (**7b**): According to the method B (r.t., 1 h), compound **7b** was obtained as a colorless solid (418 mg, 91%), mp > 265 °C; ^1^H-NMR (DMSO-*d*_6_) δ 10.17 (s, 1H, NH), 10.06 (s, 1H, NH), 8.26 (d, *J* = 1.8 Hz, 1H, H_5_), 8.23 (s, 1H, H_2_), 7.93 (dd, *J* = 8.7, 1.8 Hz, 1H, H_7_), 7.63 (d, *J* = 8.7 Hz, 1H, H_8_), 7.53 (d, *J* = 8.7 Hz, 2H, ArH), 7.42 (d, *J* = 8.7 Hz, 2H, ArH), 3.28–3.18 (m, 1H, NCH), 1.08–0.95 (m, 4H, CH); ^13^C-NMR (DMSO-*d*_6_) δ 179.8, 161.1, 147.0, 144.2, 138.2, 130.2, 128.5, 128.4, 127.2, 125.3, 121.3, 119.0, 29.2, 6.0; ν_max_ 3290, 1645, 1506, 1488, 1305, 1245, 1087, 787, 654, 525 cm^−1^; HRMS calcd for C_18_H_16_N_4_OS Cl [M + H]^+^ 371.0733 found 371.0721.

*N-(3-Cyclopropyl-4-oxo-3,4-dihydroquinazolin-6-yl)-N′-(4-fluorophenyl)thiourea* (**7c**): According to the method A (r.t., 2 h), compound **7c** was isolated as a colorless solid (331 mg, 90%), mp. 194–196 °C; ^1^H-NMR (DMSO-*d*_6_) δ 10.07 (s, 1H, NH), 9.94 (s, 1H, NH), 8.26 (d, *J* = 2.4 Hz, 1H, H_5_), 8.22 (s, 1H, H_2_), 7.93 (dd, *J* = 8.7, 2.4 Hz, 1H, H_7_), 7.62 (d, *J* = 8.7 Hz, 1H, H_8_), 7.50–7.46 (m, 2H, ArH), 7.22–7.16 (m, 2H, ArH), 3.25–3.20 (m, 1H, NCH), 1.08–0.94 (m, 4H, CH); ^13^C-NMR (DMSO-*d*_6_) δ 180.1, 161.1, 159.3 (d, *J* = 240 Hz, C4′-F), 146.9, 144.1, 138.3, 135.5, 130.2, 127.1, 126.2 (d, *J* = 8.25 Hz, 2C, C2′-F), 121.2, 119.0, 115.2 (d, *J* = 22.5 Hz, 2C, C3′-F), 29.2, 6.0; ν_max_ 3324, 3195, 3055, 1651, 1633, 1606, 1510, 1300, 1221, 836, 717, 545 cm^−1^; HRMS calcd for C_18_H_16_N_4_OFS [M + H]^+^ 355.1029 found 355.1014.

*N-(3-Cyclopropyl-4-oxo-3,4-dihydroquinazolin-6-yl)-N′-[4-(trifluoromethyl)phenyl)]-thiourea* (7**d**): According to the method A (40 °C, 1 h), compound **7d** was isolated as a light yellow solid (351 mg, 84%), mp. 198–200 °C; ^1^H-NMR (DMSO-*d*_6_) δ 10.33 (s, 1H, NH), 10.29 (s, 1H, NH), 8.28 (d, *J* = 2.7 Hz, 1H, H_5_), 8.23 (s, 1H, H_2_), 7.94 (dd, *J* = 8.7, 2.7 Hz, 1H, H_7_), 7.76 (d, *J* = 8.7 Hz, 2H, ArH), 7.67 (d, *J* = 8.7 Hz,2H, ArH), 7.64 (d, *J* = 8.7 Hz, 1H, H_8_), 3.25–3.21 (m, 1H, NCH), 1.08–0.94 (m, 4H, CH); ^19^F NMR (282 MHz, DMSO-*d*_6_) δ −60.4 (3F, CF_3_); ^13^C-NMR (DMSO-*d*_6_) δ 179.8, 161.1, 147.1, 144.3, 143.1, 138.0, 130.2, 127.2, 125.7 (q, *J* = 37.5 Hz, 2C, C3′-F), 124.1 (q, *J* = 32.0 Hz, C4′-F), 122.9, 121.2, 119.0, 29.2, 6.0; ν_max_ 3167, 2988, 1684, 1604, 1487, 1323, 1289, 1190, 1068, 835, 613 cm^−1^; HRMS calcd for C_19_H_16_N_4_OF_3_S [M + H]^+^ 405.0997 found 405.0996.

*N-(3-Cyclopropyl-4-oxo-3,4-dihydroquinazolin-6-yl)-N′-(4-nitrophenyl)thiourea* (**7e**): According to the method B (r.t., 30 min.), compound **7e** was isolated as a yellow solid (351 mg, 74%), mp. 198–200 °C; ^1^H-NMR (DMSO-*d*_6_) δ 10.56 (s, 1H, NH), 10.55 (s, 1H, NH), 8.31 (d, *J* = 2.3 Hz, 1H, H_5_), 8.24 (s, 1H, H_2_), 8.22 (d, *J* = 9.1 Hz, 2H, ArH), 7.95 (dd, *J* = 8.7, 2.3 Hz, 1H, H_7_), 7.85 (d, *J* = 8.7 Hz, 2H, ArH), 7.65 (d, *J* = 8.7 Hz, 1H, H_8_), 3.28–3.22 (m, 1H, NCH), 1.06–0.95 (m, 4H, CH); ^13^C-NMR (DMSO-*d*_6_) δ 179.5, 161.0, 147.2, 146.0, 144.4, 142.4, 137.8, 130.1, 127.3, 122.4, 121.3, 119.1, 29.2, 6.0; ν_max_ 3331, 3215, 3094, 3067, 1650, 1626, 1490, 1322, 1246, 1106, 847, 705, 527 cm^−1^; HRMS calcd for C_18_H_16_N_5_O_3_S [M + H]^+^ 382.0974 found 382.0959.

*N-(3-Cyclopropyl-4-oxo-3,4-dihydroquinazolin-6-yl)-N′-(2,4-dichlorophenyl)thiourea* (**7f**): According to the method B (r.t., 1 h), compound **7f** was isolated as a colorless solid (446 mg, 89%), mp > 265 °C; ^1^H-NMR (DMSO-*d*_6_) δ 10.28 (s, 1H, NH), 9.70 (s, 1H, NH), 8.32 (d, *J* = 2.4 Hz, 1H, H_5_), 8.24 (s, 1H, H_2_), 7.95 (dd, *J* = 2.4, 8.7 Hz, 1H, H_7_), 7.72 (d, *J* = 2.4 Hz, 1H, H_3′_); 7.64 (d, *J* = 8.7 Hz, 1H, H_8_), 7.61 (d, *J* = 8.4 Hz, 1H, H_6′_), 7.46 (dd, *J* = 2.4, 8.4 Hz, 1H, H_5′_), 3.28–3.20 (m, 1H, NCH), 1.08–0.95 (m, 4H, CH); ^13^C-NMR (DMSO-*d*_6_) δ 180.5, 161.1, 147.1, 144.3, 138.0, 135.4, 131.4, 131.2, 131.0, 130.2, 129.0, 127.5, 127.2, 121.3, 119.3, 29.2, 6.0; ν_max_ 3234, 1650, 1518, 1445, 1346, 1209, 840, 808, 665, 499 cm^−1^; HRMS calcd for C_18_H_15_N_4_OSCl_2_ [M + H]^+^ 405.0344 found 405.0344.

*N-(3-Cyclopropyl-4-oxo-3,4-dihydroquinazolin-6-yl)-N′-(pyridin-3-yl)thiourea* (**7g**): According to the method A (40 °C (µW), 1 h), compound **7g** was isolated as a colorless solid (309 mg, 89%), mp. 188–190 °C; ^1^H-NMR (DMSO-*d*_6_) δ 10.29 (s, 1H, NH), 10.05 (s, 1H, NH), 8.62 (d, *J* = 2.4 Hz, 1H, H_2′_), 8.35 (d, *J* = 4.5 Hz, 1H, H_6′_), 8.27 (d, J = 2.1 Hz, 1H, H_5_), 8.24 (s, 1H, H_2_), 7.96–7.92 (m, 2H, H_7_ + H_4′_), 7.64 (d, *J* = 8.7 Hz, 1H, H_8_), 7.39 (dd, *J* = 2.4, 4.5 Hz, 1H, H_5′_), 3.27–3.22 (m, 1H, NCH), 1.06–0.95 (m, 4H, CH); ^13^C-NMR (DMSO-*d*_6_) δ 180.4, 161.1, 147.0, 145.44, 145.37, 144.3, 138.0, 136.1, 131.4, 130.3, 127.2, 123.2, 121.3, 119.1, 29.2, 6.0; ν_max_ 3217, 3011, 1656, 1539, 1485, 1363, 1257, 839, 707, 558 cm^−1^; HRMS calcd for C_17_H_16_N_5_OS [M + H]^+^ 338.1076 found 338.1071.

*N-(3-Cyclopropyl-4-oxo-3,4-dihydroquinazolin-6-yl)-N′-(p-tolyl)thiourea* (**7h**): According to the method B (r.t., 2 h), compound **7h** was obtained as a colorless solid (386 mg, 89%), mp > 265 °C; ^1^H-NMR (DMSO-*d*_6_) δ 9.99 (s, 1H, NH), 9.90 (s, 1H, NH), 8.27 (d, *J* = 1.8 Hz, 1H, H_5_), 8.22 (s, 1H, H_2_), 7.94 (dd, *J* = 8.7, 1.8 Hz, 1H, H_7_), 7.61 (d, *J* = 8.7 Hz, 1H, H_8_), 7.35 (d, *J* = 8.1 Hz, 2H, ArH), 7.16 (d, *J* = 8.1 Hz, 2H, ArH), 3.26–3.19 (m, 1H, NCH), 2.30 (s, 3H, CH_3_), 1.05–0.94 (m, 4H, CH); ^13^C-NMR (DMSO-*d*_6_) δ 179.7, 161.1, 146.8, 144.0, 138.5, 136.5, 133.9, 130.2, 129.0, 127.0, 123.9, 121.2, 118.8, 29.2, 20.5, 6.0; ν_max_ 3290, 3072, 2993, 1648, 1596, 1513, 1492, 1303, 1246, 1163, 1037, 827, 654, 608, 580 cm^−1^; HRMS calcd for C_19_H_19_N_4_OS [M + H]^+^ 351.1280 found 351.1276.

*N-(3-Cyclopropyl-4-oxo-3,4-dihydroquinazolin-6-yl)-N′-(4-methoxyphenyl)thiourea* (**7i**): According to the method B (r.t., 2 h), compound **7i** was isolated as a colorless solid (395 g, 87%), mp > 265 °C; ^1^H-NMR (DMSO-*d*_6_) δ 9.91 (s, 1H, NH), 9.81 (s, 1H, NH), 8.27 (d, *J* = 1.8 Hz, 1H, H_5_), 8.22 (s, 1H, H_2_), 7.94 (dd, *J* = 8.7, 1.8 Hz, 1H, H_7_), 7.61 (d, *J* = 8.7 Hz, 1H, H_8_), 7.34 (d, *J* = 8.1 Hz, 2H, ArH), 6.93 (d, *J* = 8.1 Hz, 2H, ArH), 3.76 (s, 3H, OCH_3_), 3.26–3.19 (m, 1H, NCH), 1.05–0.94 (m, 4H, CH); ^13^C-NMR (DMSO-*d*_6_) δ 180.0, 161.1, 156.7, 146.8 144.0, 138.54 131.8, 130.2, 127.0, 126.0, 121.2, 118.9, 113.8, 55.2, 29.2, 6.0; ν_max_ 3296, 1648, 1565, 1514, 1306, 817, 719, 574 cm^−1^; HRMS calcd for C_19_H_19_N_4_O_2_S [M + H]^+^ 367.1229 found 367.1222.

*N-(3-Cyclopropyl-4-oxo-3,4-dihydroquinazolin-6-yl)-N′-(4-methoxyphenyl)thiourea* (**7j**): According to the method A (r.t., 10 min.), compound **7j** was isolated as a grey solid (340 mg, 87%), mp. 210–212 °C; ^1^H-NMR (DMSO-*d*_6_) δ 9.77 (s, 1H, NH), 9.70 (s, 1H, NH), 8.27 (d, *J* = 2.4 Hz, 1H, H_5_), 8.20 (s, 1H, H_2_), 7.93 (dd, *J* = 8.7, 2.4 Hz, 1H, H_7_), 7.59 (d, *J* = 8.7 Hz, 1H, H_8_), 7.22 (d, *J* = 9.0 Hz, 2H, ArH), 6.72 (d, *J* = 9.0 Hz, 2H, ArH), 2.89 (s, 6H, NCH_3_), 3.33–3.21 (m, 1H, NCH), 1.05–0.93 (m, 4H, CH); ^13^C-NMR (DMSO-*d*_6_) δ 179.7, 161.1, 148.3, 146.7, 143.9, 138.7, 130.2, 127.9, 126.9, 125.7, 121.1, 118.8, 112.3, 40.3, 29.1, 6.0; ν_max_ 3290, 3245, 1649, 1524, 1311, 1243, 704 cm^−1^; HRMS calcd for C_20_H_22_N_5_OS [M + H]^+^ 380.1545 found 380.1530.

*N-(3-Cyclopropyl-4-oxo-3,4-dihydroquinazolin-6-yl)-N′-(2,4-dimethoxyphenyl)thiourea* (**7k**): According to the method A (r.t., 30 min.), product **7k** was isolated as a black solid (396 mg, 97%), mp. 166–168 °C; ^1^H-NMR (DMSO-*d*_6_) δ 9.90 (s, 1H, NH), 9.22 (s, 1H, NH), 8.33 (d, *J* = 2.4 Hz, 1H, H_5_), 8.21 (s, 1H, H_2_), 7.95 (dd, *J* = 8.7, 2.4 Hz, 1H, H_7_), 7.60 (d, *J* = 8.7 Hz, 1H, H_8_), 7.47 (d, *J* = 8.4 Hz, 1H, H_6′_), 6.64 (d, *J* = 2.7 Hz, 1H, H_3′_), 6.53 (dd, *J* = 8.4, 2.7 Hz, 1H, H_5′_), 3.83 (s, 3H, OCH_3_), 3.78 (s, 3H, OCH_3_), 3.25–3.20 (m, 1H, NCH), 1.05–0.94 (m, 4H, CH); ^13^C-NMR (DMSO-*d*_6_) δ 180.1, 161.1, 159.5, 154.2, 146.8, 143.9, 138.6, 130.1, 128.2, 126.9, 121.1, 120.1, 118.8, 104.3, 99.0, 55.7, 55.3, 29.1, 6.0; ν_max_ 3156, 2969, 1674, 1601, 1510, 1487, 1282, 1236, 1206, 1031, 834, 686, 540 cm^−1^; HRMS calcd for C_20_H_21_N_4_O_3_S [M + H]^+^ 397.1334 found 397.1319.

*N-(3-Cyclopropyl-4-oxo-3,4-dihydroquinazolin-6-yl)-N′-(3,4-dimethoxyphenyl)thiourea* (**7l**): According to the method A (r.t., 5 min.), compound **7l** was isolated as a black solid (359 mg, 88%), mp. 196–198 °C; ^1^H-NMR (DMSO-*d*_6_) δ 9.87 (s, 1H, NH), 9.83 (s, 1H, NH), 8.24 (d, *J* = 2.4 Hz, 1H, H_5_), 8.21 (s, 1H, H_2_), 7.93 (dd, *J* = 8.7, 2.4 Hz, 1H, H_7_), 7.60 (d, *J* = 8.7 Hz, 1H, H_8_), 7.10 (s, 1H, ArH), 6.93 (s, 2H, ArH), 3.75 (s. 3H, OCH_3_), 3.74 (s, 3H, OCH_3_), 3.26–3.16 (m, 1H, NCH), 1.05–0.94 (m, 4H, CH); ^13^C-NMR (DMSO-*d*_6_) δ 179.7, 161.1, 148.4, 146.8, 146.4, 144.1, 138.5, 131.9, 130.4, 126.9, 121.2, 119.1, 116.5, 111.7, 109.3, 55.7, 55.5, 29.1, 6.0; ν_max_ 3271, 1670, 1602, 1511, 1233, 1130, 1024, 835, 566 cm^−1^; HRMS calcd for C_20_H_21_N_4_O_3_S [M + H]^+^ 397.1334 found 397.1342.

#### 3.2.2. General Procedure for the Synthesis of 8-Cyclopropyl-2-arylaminothiazolo[5,4-*f*]quinazolin-9(8H)-ones **8d**–**g** and 3-Cyclopropyl-6-[(benzo[*d*]thiazol-2-yl)amino]quinazolin-4(3*H*)-ones **10a**; **10l** via the Hügershoff Reaction

To a solution of appropriate thiourea derivative **7** (125 mg, 1 equiv.) in acetic acid (0.2 M solution) maintained at 0 °C was added dropwise bromine (Br_2_, 1.0 equiv.). The resulting mixture was warmed to room temperature and stirred for 30 min. to 4 h. On completion, the solvent was removed under *vacuum*. The crude residue was dissolved in dichloromethane (25 mL). The organic layer was washed with a saturated solution of NaHCO_3_, then with water and brine. Evaporation of solvent gave a crude mixture which was adsorbed on Celite^®^ and purified by flash chromatography using ethyl acetate and methylene chloride (100:0 to 0:100%, *v/v*) as eluent to furnish the expected fused 2-arylaminothiazole **8** or **10**.

*8-Cyclopropyl-2-([4-(trifluoromethyl)phenyl]amino)thiazolo[5,4-f]quinazolin-9(8H)-one* (**8d**): from **7d** (125 mg, 0.31 mmol) in presence of bromine (16.0 µL, 0.31 mmol, 1.0 equiv.), 1 h at room temperature. After purification, **8d** was isolated as a colorless solid (98 mg, 79%), mp > 265 °C; ^1^H-NMR (DMSO-*d*_6_) δ 11.06 (s, 1H, NH), 8.31 (s, 1H, H_2_), 8.09 (d, *J* = 8.2 Hz, 1H, H_4_), 8.03 (d, *J* = 9.0 Hz, 2H, Ph), 7.75 (d, *J* = 9.0 Hz, 2H, Ph), 7.67 (d, *J* = 8.2 Hz, 1H, H_5_), 3.36–3.28 (m, 1H, NCH), 1.10–1.03 (m, 4H, CH); ^19^F NMR (282 MHz, DMSO-*d*_6_) δ −60.4 (3F, CF_3_); ^13^C-NMR (DMSO-*d*_6_) δ 164.5, 160.5, 150.7, 146.2, 143.8, 144.3, 143.1, 126.3, 125.9, 125.6, 124.8 (q, *J* = 37.5 Hz, 2C, C3′-F), 124.1 (q, *J* = 32.0 Hz, C4′-F), 122.9 (2C), 121.2, 117.5, 115.4, 29.3, 5.9; ν_max_ 3263, 3061, 3018, 1661, 1532, 1318, 1066, 1108, 831 cm^−1^; HRMS calcd for C_19_H_14_N_4_OSF_3_ [M + H]^+^ 403.0842 found 403.0840.

*8-Cyclopropyl-2-[(4-nitrophenyl)amino]thiazolo[5,4-f]quinazolin-9(8H)-one* (**8e**): from **7e** (125 mg, 0.33 mmol) in presence of bromine (16.8 µL, 0.33 mmol, 1.0 equiv.), 4 h at room temperature. After purification, **8e** was isolated as a yellow solid (108 mg, 86%), mp > 265 °C; ^1^H-NMR (DMSO-*d*_6_) δ 11.41 (s, 1H, NH), 8.34 (s, 1H, H_7_), 8.31 (d, *J* = 9.3 Hz, 2H, Ph), 8.15 (d, *J* = 8.6 Hz, 1H, H_4_), 8.07 (d, *J* = 9.3 Hz, 2H, Ph), 7.72 (d, *J* = 8.6 Hz, 1H, H_5_), 3.38–3.24 (m, 1H, NCH), 1.10–1.01 (m, 4H, CH); ^13^C-NMR (DMSO-*d*_6_) δ 164.0, 160.5, 150.4, 146.4, 146.2, 144.0, 140.8, 125.9, 125.3, 125.2, 117.1, 115.4, 29.3, 5.9; ν_max_ 3291, 3111, 1643, 1560, 1500, 1327, 1307, 1254, 1110, 826 cm^−1^; HRMS calcd for C_18_H_14_N_5_O_3_S [M + H]^+^ 380.0817 found 380.0824.

*8-Cyclopropyl-2-(2,4-dichlorophenylamino)thiazolo[5,4-f]quinazolin-9(8H)-one* (**8f**): from **7f** (125 mg, 0.31 mmol) in presence of bromine (15.8 µL, 0.31 mmol, 1.0 equiv.), 1 h 30 at room temperature. After purification, **8f** was isolated as a colorless solid (112 mg, 90%), mp > 265 °C; ^1^H-NMR (DMSO-*d*_6_) δ 10.25 (s, 1H, NH), 8.47 (d, *J* = 8.9 Hz, 1H, H_6′_), 8.31 (s, 1H, H_7_), 8.03 (d, *J* = 8.6 Hz,1H, H_4_), 7.71 (d, *J* = 2.4 Hz, 1H, H_3′_), 7.65 (d, *J* = 8.4 Hz, 1H, H_5_), 7.51 (dd, *J* = 2.4, 8.6 Hz, 1H, H_5′_), 3.33–3.28 (m, 1H, NCH), 1.08–1.01 (m, 4H, CH); ^13^C-NMR (DMSO-*d*_6_) δ 165.9, 160.5, 150.4, 146.1, 143.6, 136.0, 129.1, 127.8, 127.5, 125.4, 124.9, 124.7, 124.0, 115.4, 29.3, 6.0; ν_max_ 3217, 3094, 1662, 1584, 1521, 1456, 1298, 901, 831, 622, 536 cm^−1^; HRMS calcd for C_18_H_13_N_4_OSCl_2_ [M + H]^+^ 403.0187 found 403.0180.

*8-Cyclopropyl-2-(pyridin-3-ylamino)thiazolo[5,4-f]quinazolin-9(8H)-one* (**8g**): from **7g** (125 mg, 0.37 mmol) and bromine (19.0 µL, 0.37 mmol, 1.0 equiv.), 2 h at room temperature. After purification, **8g** was isolated as a colorless solid (100 mg, 80%), mp > 265 °C; ^1^H-NMR (DMSO-*d*_6_) δ 10.89 (s, 1H, NH), 8.94 (d, *J* = 2.4 Hz, 1H, H_2′_), 8.39 (dd, *J* = 2.4, 8.2 Hz, 1H, H_6′_), 8.31 (s, 1H, H_7_), 8.26 (dd, *J* = 2.4, 4.5 Hz, 2H, H_4′_), 8.08 (d, *J* = 8.6 Hz, 1H, H_4_), 7.67 (d, *J* = 8.6 Hz, 1H, H_5_), 7.43 (dd, *J* = 4.5, 8.2 Hz, 1H, H_5′_), 3.38–3.24 (m, 1H, NCH), 1.10–1.01 (m, 4H, CH); ^13^C-NMR (DMSO-*d*_6_) δ 164.8, 160.5, 150.8, 146.1, 143.6, 142.9, 139.5, 137.2, 125.6, 125.5, 125.0, 124.3, 123.8, 115.4, 29.3, 5.9; ν_max_ 3659, 3256, 2973, 1662, 1521, 1426, 1280, 826, 799 cm^−1^; HRMS calcd for C_17_H_14_N_5_OS [M + H]^+^ 336.0919 found 336.0910.

*3-Cyclopropyl-6-[(benzo[d]thiazol-2-yl)amino]quinazolin-4(3H)-one* (**10a**): from **7a** (125 mg, 0.37 mmol) in presence of bromine (18.9 µL, 0.37 mmol, 1.0 equiv.), after 1 h at room temperature. After purification, **10a** was isolated as a colorless solid (37 mg, 30%), mp. 258–260 °C; ^1^H-NMR (DMSO-*d*_6_) δ 10.93 (s, 1H, NH), 8.72 (d, *J* = 2.3 Hz, 1H, H_5_), 8.18 (s, 1H, H_2_), 8.10 (dd, *J* = 2.3, 8.7 Hz, 1H, H_7_), 7.85 (d, *J* = 8.7 Hz, 1H, H_8_), 7.69–7.64 (m, 2H, H_5′_+H_6′_), 7.40–7.35 (m, 1H, H_4′_), 7.22–7.17 (m, 1H, H_7′_), 3.31–3.21 (m, 1H, NCH), 1.09–0.95 (m, 4H, CH); ^13^C-NMR (DMSO-*d*_6_) δ 161.3, 161.2, 151.9, 146.0, 142.5, 139.4, 130.1, 128.0, 126.1, 124.7, 122.7, 122.0, 121.3, 119.5, 112.4, 29.2, 6.0; ν_max_ 3401, 1657, 1623, 1599, 1537, 1490, 1315 cm^−1^; HRMS calcd for C_18_H_15_N_4_OS [M + H]^+^ 335.0967 found 335.0966.

*3-Cyclopropyl-6-((5,6-dimethoxybenzo[d]thiazol-2-yl)amino)quinazolin-4(3H)-one* (**10l**): from **7l** (125 mg, 0.32 mmol) and bromine (16.4 µL, 0.32 mmol, 1.0 equiv.), 30 min at room temperature. After purification, **10l** was isolated as a colorless solid (113 mg, 90%), mp. 200 °C; ^1^H-NMR (DMSO-*d*_6_) δ 10.73 (s, 1H, NH), 8.71 (d, *J* = 2.5 Hz, 1H, H_5_), 8.35 (s, 1H, H_2_), 8.07 (dd, *J* = 2.5, 8.8 Hz, 1H, H_7_), 7.68 (d, *J* = 8.8 Hz, 1H, H_8_), 7.48 (s, 1H, H_4′_), 7.28 (s, 1H, H_7′_), 3.85 (s, 3H, OCH_3_), 3.79 (s, 3H, OCH_3_), 3.33–3.28 (m, 1H, NCH), 1.08–1.01 (m, 4H, CH); ^13^C-NMR (DMSO-*d*_6_) δ 160.3, 160.1, 150.4, 148.6, 147.3, 146.1, 145.3, 140.3, 137.5, 125.0, 121.4, 120.7, 104.3, 103.3, 56.0, 55.7, 30.0, 6.0; ν_max_ 3380, 3240, 2999, 1693, 1552, 1482, 1219, 625, 527 cm^−1^; HRMS calcd for C_20_H_19_N_4_O_3_S [M + H]^+^ 395.1178 found 395.1173.

#### 3.2.3. General Procedure for the Synthesis of 8-Cyclopropyl-2-arylaminothiazolo[5,4-*f*]quinazolin-9(8*H*)-ones **8a**; **c**; **h**–**n** and 8-Cyclopropyl-2-alkylaminothiazolo[5,4-*f*]quinazolin-9(8*H*)-ones **14a**–**f** from 5-Bromo-3-cyclopropyl-6-isothiocyanatoquinazolin-4(3*H*)-one (**13**) via a One-Pot Regio-Controlled Copper(I) Mediated C-S Coupling of Intermediates **7**

To a solution of 5-bromo-3-cyclopropyl-6-isothiocyanatoquinazolin-4(3*H*)-one (**13**, 100 mg, 0.31 mmol, 1.0 equiv.) in DME (3 mL) was added the appropriate amine (0.33 mmol, 1.05 equiv.) at room temperature for 30 min. to 12 h. After completion, copper(I) iodide (5.7 mg, 0.03 mmol, 10 mol %) and cesium carbonate (208 mg, 0.64 mmol, 2.0 equiv.) were added and the resulting suspension was heated under microwave at 80 °C for 1 h. The resulting mixture was adsorbed on Celite^®^ and purified by flash chromatography using ethyl acetate/methylene chloride (0/100 to 50/50, *v/v*) as eluent to furnish the expected fused 2-arylaminothiazoloquinazolinones **8a**; **c**; **h**–**n** and **14a**; **f** or methanol/methylene chloride (0:100 to 5:95, *v/v*) as eluent to furnish the 2-alkylaminothiazoloquinazolinones **14b**–**e**.

*8-Cyclopropyl-2-(phenylamino)thiazolo[5,4-f]quinazolin-9(8H)-one* (**8a**): According to the general procedure, thiourea intermediate was obtained from **13** and aniline (30.7 mg) after 12 h at room temperature. After purification, **8a** was isolated as a colorless solid (82.9 mg, 80%), mp. 204–206 °C; ^1^H-NMR (DMSO-*d*_6_) δ 10.69 (s, 1H, NH), 8.30 (s, 1H, H_7_), 8.04 (d, *J* = 8.7 Hz, 1H, H_4_), 7.85–7.83 (m, 2H, H_2_ + H_6′_), 7.64 (d, *J* = 8.7 Hz, 1H, H_5_), 7.41–7.37 (m, 2H, Ph), 7.07–7.03 (m, 1H, Ph), 3.38–3.24 (m, 1H, NCH), 1.09–1.02 (m, 4H, CH); ^13^C-NMR (75 MHz, -*d*_6_) δ 165.1, 160.6, 151.2, 145.9, 143.3, 140.5, 129.0 (2C), 125.5, 125.2, 124.8, 122.2, 117.8, 115.4, 20.3, 6.0; ν_max_ 3257, 3078, 1674, 1516, 1317, 1243, 828, 751, 696 cm^−1^; HRMS calcd for C_18_H_15_N_4_OS [M + H]^+^ 335.0967 found 335.0951.

*8-Cyclopropyl-2-[(4-fluorophenyl)amino]thiazolo[5,4-f]quinazolin-9(8H)-one* (**8c**): According to the general procedure, thiourea intermediate was obtained from **13** and 4-fluoroaniline (56.8 mg) after 2 h at room temperature. After purification, **8c** was isolated as a white solid (104.9 mg, 96%), mp > 265 °C; ^1^H-NMR (300 MHz,DMSO-*d*_6_) δ 10.69 (s, 1H, NH), 8.29 (s, 1H, H_7_), 8.05 (d, *J* = 8.7 Hz, 1H, H_4_), 7.88–7.86 (m, 1H, Ph), 7.66 (d, *J* = 8.7 Hz, 1H, H_5_), 7.24 (m, 2H, Ph), 3.38–3.24 (m, 1H, NCH), 1.09–1.02 (m, 4H, CH); 19F NMR (282 MHz, DMSO-*d*_6_) δ −120.8; ^13^C-NMR (DMSO-*d*_6_) δ 165.1, 160.6, 157.4 (d, *J* = 237 Hz, C4′-F), 151.1, 145.9, 143.4, 137.0, 125.4, 125.2, 124.9, 119.5 (d, *J* = 7.50 Hz, 2C, C2′-F), 115.7, 115.4 (d, *J* = 22.5 Hz, 2C, C3′-F), 29.2, 5.9 (2C); ν_max_ 3291, 3111, 1643, 1560, 1500, 1327, 1307, 1254, 1110, 826 cm^−1^; HRMS calcd for C_18_H_14_N_4_OSF [M + H]^+^ 353.0872 found 353.0861.

*8-Cyclopropyl-2-(p-tolylamino)thiazolo[5,4-f]quinazolin-9(8H)-one* (**8h**): According to the general procedure, thiourea intermediate was obtained from **13** and *p*-toluidine (35.4 mg,) after 2 h at room temperature. After purification, **8h** was isolated as a colorless solid (96 mg, 89%), mp > 265 °C; ^1^H-NMR (DMSO-*d*_6_) δ 10.56 (s, 1H, NH), 8.28 (s, 1H, H_7_), 8.02 (d, *J* = 8.7 Hz, 1H, H_4_), 7.71 (d, *J* = 8.1 Hz, 2H, Ph), 7.63 (d, *J* = 8.7 Hz, 1H, H_5_), 7.20 (d, *J* = 8.1 Hz, 2H, Ph), 3.38–3.24 (m, 1H, NCH), 2.29 (s, 3H, CH_3_), 1.10–1.01 (m, 4H, CH); ^13^C-NMR (75 MHz,DMSO-*d*_6_) δ 165.2, 160.6, 151.3, 145.8, 143.3, 138.0, 131.1, 129.4, 125.4, 125.1, 124.8, 118.0, 115.4, 29.3, 20.4, 5.9; ν_max_ 3191, 3041, 1640, 1564, 1501, 1327, 1317, 1254, 1140, 840 cm^−1^; HRMS calcd for C_19_H_17_N_4_OS [M + H]^+^ 349.1118 found 349.1123.

*8-Cyclopropyl-2-[(4-methoxyphenyl]amino]thiazolo[5,4-f]quinazolin-9(8H)-one* (**8i**): According to the general procedure, thiourea intermediate was obtained from **13** and *p*-anisidine (40.6 mg) after 2 h at room temperature. After purification, **8i** was isolated as a colorless solid (99.4 mg, 88%), mp > 265 °C; ^1^H-NMR (DMSO-*d*_6_) δ 10.50 (s, 1H, NH), 8.26 (s, 1H, H_7_), 7.78 (d, *J* = 8.7 Hz, 1H, H_4_), 7.72 (d, *J* = 9.0 Hz, 2H, Ph), 7.62 (d, *J* = 8.7 Hz, 1H, H_5_), 6.98 (d, *J* = 9.0 Hz, 2H, Ph), 3.78 (s, 3H, OCH_3_), 3.38–3.26 (m, 1H, NCH), 1.08–1.01 (m, 4H, CH); ^13^C-NMR (DMSO-*d*_6_) δ 165.6, 160.6, 154.8, 151.4, 145.7, 143.1, 133.8, 126.5, 125.3, 124.9, 124.8, 119.8, 115.4, 114.2, 115.4, 55.2, 29.1, 5.9; ν_max_ 3267, 3195, 2994, 1679, 1565, 1505, 1295, 1228, 1029, 826, 522 cm^−1^; HRMS calcd for C_19_H_17_N_4_O_2_S [M + H]^+^ 365.1072 found 365.1066.

*8-Cyclopropyl-2-([4-(dimethylamino)phenyl]amino)thiazolo[5,4-f]quinazolin-9(8H)-one* (**8j**): According to the general procedure, thiourea intermediate was obtained from **13** and 4-(dimethylamino)aniline (44.9 mg) after 1 h at room temperature. After purification, **8j** was isolated as a pale yellow solid (101.7 mg, 87%), mp > 265 °C; ^1^H-NMR (DMSO-*d*_6_) δ 10.27 (s, 1H, NH), 8.24 (s, 1H, H_7_), 7.94 (d, *J* = 8.7 Hz, 1H, H_4_), 7.61–7.56 (m, 3H, Ph + H_5_), 7.78 (d, *J* = 9.1 Hz, 1H, Ph), 3.38–3.24 (m, 1H, NCH), 2.88 (s, 6H, NCH_3_), 1.10–1.01 (m, 4H, CH); ^13^C-NMR (DMSO-*d*_6_) δ 166.2, 160.6, 151.7, 146.9, 145.5, 142.9, 130.4, 125.3, 124.7, 120.3, 115.4, 113.1, 40.3, 29.2, 5.9; ν_max_ 3184, 2992, 1644, 1574, 1501, 1427, 1347, 1244, 840 cm^−1^; HRMS calcd for C_20_H_20_N_5_OS [M + H]^+^ 378.1389 found 378.1376.

*8-Cyclopropyl-2-[(2,4-dimethoxyphenyl)amino]thiazolo[5,4-f]quinazolin-9(8H)-one* (**8k**): According to the general procedure, thiourea intermediate was obtained from **13** and 2,4-dimethoxyaniline (50.5 mg) after 30 min. at room temperature. After purification, **8k** was isolated as a brown solid (105.1 mg, 86%), mp. 240–242 °C; ^1^H-NMR (DMSO-*d*_6_) δ 9.75 (s, 1H, NH), 8.24 (s, 1H, H_7_), 8.03 (d, *J* = 8.7 Hz, 1H, H_6′_), 7.92 (d, *J* = 8.6 Hz, 1H, H_4_), 7.59 (d, 1H, *J* = 8.6 Hz, H_5_), 6.69 (d, *J* = 2.4 Hz, 1H, H_3′_), 6.60 (dd, *J* = 2.4, 8.7 Hz, 1H, H_5′_), 3.85 (s, 3H, OCH_3_), 3.80 (s, 3H, OCH_3_), 3.38–3.24 (m, 1H, NCH), 1.10–1.01 (m, 4H, CH); ^13^C-NMR (DMSO-*d*_6_) δ 167.9, 160.6, 151.8, 151.4, 145.5, 142.9, 125.9, 124.6, 124.5, 123.4, 122.3, 115.4, 104.4, 99.1, 55.7, 55.3, 29.2, 5.9; ν_max_ 3402, 3061, 2932, 2826, 1669, 1563, 1532, 1451, 1178, 1023, 822, 514 cm^−1^; HRMS calcd for C_20_H_19_N_4_O_3_S [M + H]^+^ 395.1178 found 395.1178.

*8-Cyclopropyl-2-[(3,4-dimethoxyphenyl)amino]thiazolo[5,4-f]quinazolin-9(8H)-one* (**8l**): According to the general procedure, thiourea intermediate was obtained from **13** and 4-aminoveratrole (50.5 mg) after 30 min. at room temperature. After purification, **8l** was isolated as a colorless solid (110 mg, 90%), mp > 265 °C; ^1^H-NMR (DMSO-*d*_6_) δ 10.47 (s, 1H, NH), 8.27 (s, 1H, H_7_), 7.99 (d, *J* = 8.6 Hz, 1H, H_4_), 7.63 (d, *J* = 8.6 Hz, 1H, H_5_), 7.46 (d, *J* = 2.4 Hz, 1H, H_2′_), 7.34 (d, *J* = 2.4, 8.8 Hz, 1H, H_6′_), 6.98 (d, *J* = 8.8 Hz, 1H, H_5′_), 3.81 (s, 3H, OCH_3_), 3.75 (s, 3H, OCH_3_), 3.38–3.24 (m, 1H, NCH), 1.08–1.01 (m, 4H, CH); ^13^C-NMR (DMSO-*d*_6_) δ 165.5, 160.6, 151.4, 148.9, 145.7, 144.4, 143.2, 134.2, 125.3, 125.0, 124.8, 115.4, 112.5, 110.1, 103.8, 55.8, 55.4, 29.2, 5.9; ν_max_ 3273, 3201, 2937, 2832, 1659, 1564, 1516, 1455, 1226, 1025, 826, 537 cm^−1^; HRMS calcd for C_20_H_19_N_4_O_3_S [M + H]^+^ 395.1178 found 395.1166.

*4-[(8-Cyclopropyl-9-oxo-8,9-dihydrothiazolo[5,4-f]quinazolin-2-yl)amino]benzenesulfonamide* (**8m**): According to the general procedure, thiourea intermediate was obtained from **13** and 4-aminobenzenesulfonamide (56.8 mg) after 12 h at room temperature. After purification, **8l** was isolated as a beige solid (128.1 mg, 77%), mp > 265 °C; ^1^H-NMR (DMSO-*d*_6_) δ 11.10 (s, 1H, NH), 8.30 (s, 1H, H_7_), 8.10 (d, *J* = 8.6 Hz, 1H, H_4_), 8.00 (d, J = 8.8, 2H, Ph), 7.83 (d, *J* = 8.8 Hz, Ph), 7.67 (d, *J* = 8.6 Hz, 1H, H_5_), 7.28 (br, 2H, NH_2_), 3.39–3.28 (m, 1H, NCH), 1.10–1.01 (m, 4H, CH); ^13^C-NMR (DMSO-*d*_6_) δ 164.5, 160.5, 150.7, 146.2, 143.7, 143.2, 136.9, 127.0, 125.6, 125.0, 117.2, 115.4, 29.3, 6.0; ν_max_ 3486, 3296, 3067, 1655, 1591, 1556, 1518, 1153, 832, 534 cm^−1^; HRMS calcd for C_18_H_16_N_5_O_3_S_2_ [M + H]^+^ 414.0695 found 414.0692.

*2-(Benzylamino)-8-cyclopropylthiazolo[5,4-f]quinazolin-9(8H)-one* (**14a**): According to the general procedure, thiourea intermediate was obtained from **13** and benzylamine (35.4 mg) after 30 min. at room temperature. After purification, **14a** was isolated as a colorless solid (91.8 mg, 85%), mp. 262–264 °C; ^1^H-NMR (DMSO-*d*_6_) δ 8.73 (t, *J* =5.7 Hz, 1H, NH), 8.21 (s, 1H, H_7_), 7.84 (d, *J* = 8.7 Hz, 1H, H_4_), 7.55 (d, *J* = 8.7 Hz, 1H, H_5_), 7.40–7.27 (m, 5H Ph), 4.64 (d, *J* =5.7 Hz, 2H, CH_2_), 3.33–3.28 (m, 1H, NCH), 1.07–0.99 (m, 4H, CH); ^13^C-NMR (DMSO-*d*_6_) δ 169.8, 160.5, 151.6, 145.2, 142.4, 138.7, 128.4 (2C), 127.4 (2C), 127.1, 125.7, 124.5, 124.2, 115.4, 47.2, 29.1, 5.9; ν_max_ 3223, 3067, 2907, 1658, 1589, 1455, 1311, 826, 703, 686 cm^−1^; HRMS calcd for C_19_H_17_N_4_OS [M + H]^+^ 349.1117 found 349.1123.

*8-Cyclopropyl-2-([2-(dimethylamino)ethyl]amino)thiazolo[5,4-f]quinazolin-9(8H)-one* (**14b**): According to the general procedure, thiourea intermediate was obtained from **13** and *N,N*-dimethylethylenediamine (29.1 mg) after 30 min. at room temperature. After purification, **14b** was isolated as a colorless solid (98.0 mg, 96%), mp. 210–212 °C; ^1^H-NMR (DMSO-d_6_/CDCl_3_) δ 8.12 (s, 1H, H_7_), 7.90 (d, *J* = 8.6 Hz, 1H, H_4_), 7.63 (d, 1H, *J* = 8.6 Hz, H_5_), 7.37 (s, 1H, NH), 3.70 (m, 2H, CH_2_NH), 3.35–3.30 (m, 1H, NCH), 2.89–2.85 (m, 2H, NCH_2_), 2.50 (s, 6H, NCH_3_), 1.28 (m, 2H, CH), 1.04 (m, 2H, CH); ^13^C-NMR (DMSO-d_6_/CDCl_3_) δ 170.1, 160.8, 151.6, 143.9, 142.2, 126.0, 124.2, 124.1, 115.3, 56.9, 44.3, 41.1, 25.7, 6.0; ν_max_ 3201, 2942, 2776, 1659, 1592, 1571, 1456, 1340, 1314, 1164, 825 cm^−1^; HRMS calcd for C_16_H_20_N_5_OS [M + H]^+^ 330.1389 found 330.1392.

*8-Cyclopropyl-2-([2-(piperidin-1-yl)ethyl]amino)thiazolo[5,4-f]quinazolin-9(8H)-one* (**14c**): According to the general procedure, thiourea intermediate was obtained from **13** and 2-piperiridinylethylamine (43.0 mg) after 30 min. at room temperature. After purification, **14c** was isolated as a colorless solid (104 mg, 91%), mp. 218–220 °C; ^1^H-NMR (DMSO-*d*_6_) δ 8.25–8.20 (m, 2H, H_7_ + NH), 7.82 (d, *J* = 8.4 Hz, 1H, H_4_), 7.54 (d, 1H, *J* = 8.4 Hz, H_5_), 3.56–3.54 (m, 2H, NCH_2_), 3.41–3.31 (m, 2H, CH_2_NH), 3.30–3.24 (m, 1H, NCH), 2.65–2.59 (m, 2H, CH_2_N), 1.55–1.54 (m, 4H, CH), 1.41–1.39 (m, 4H, CH), 1.10–1.01 (m, 4H,CH); ^13^C-NMR (DMSO-*d*_6_) δ 169.6, 160.5, 151.7, 145.2, 142.3, 125.6, 124.4, 124.0, 115.4, 64.8, 54.1, 53.8, 40.1, 29.1, 15.1, 5.9; ν_max_ 3279, 2960, 2860, 1660, 1566, 1529, 1453, 1297, 1113, 833 cm^−1^; HRMS calcd for C_18_H_22_N_5_O_2_S [M + H]^+^ 372.1494 found 372.1487.

*8-Cyclopropyl-2-[(2-morpholinoethyl)amino]thiazolo[5,4-f]quinazolin-9(8H)-one* (**14d**): According to the general procedure, thiourea intermediate was obtained from **13** and 2-morpholinoethylamine (43.0 mg) after 30 min. at room temperature. After purification, **14d** was isolated as a colorless solid (102 mg, 89%), mp. 222–224 °C; ^1^H-NMR (DMSO-*d*_6_) δ 8.27 (s, 1H, H_7_), 8.25 (br, 1H, NH), 7.89 (d, *J* = 8.4 Hz, 1H, H_4_), 7.61 (d, 1H, *J* = 8.4 Hz, H_5_), 3.67–3.63 (m, 4H, OCH_2_), 3.60–3.57 (m, 2H, CH_2_NH), 3.30–3.24 (m, 1H, NCH), 2.57–2.53 (m, 2H, CH_2_N), 2.44–2.42 (m, 4H, NCH_2_), 1.09–1.00 (m, 4H, CH); ^13^C-NMR (DMSO-*d*_6_) δ 169.6, 160.6, 151.8, 145.1, 142.3, 125.6, 124.4, 124.0, 115.4, 66.1, 57.0, 53.3, 41.1, 29.1, 5.9; ν_max_ 3279, 2960, 1665, 1566, 1529, 1454, 1111, 835 cm^−1^.

*8-Cyclopropyl-2-(piperidin-1-yl)thiazolo[5,4-f]quinazolin-9(8H)-one* (**14e**): According to the general procedure, thiourea intermediate was obtained from **13** and piperidine (26.3 mg) after 30 min. at room temperature. After purification, **14e** was isolated as a colorless solid (90.1 mg, 89%), mp. 244–246 °C; ^1^H-NMR (CDCl_3_) δ 8.05 (s, 1H, H_7_), 7.88 (d, *J* = 8.4 Hz, 1H, H_4_), 7.63 (d, 1H, *J* = 8.4 Hz, H_5_), 3.68 (m, 4H, NCH_2_), 3.30–3.25 (m, 1H, NCH), 1.71 (m, 6H, CH_2_), 1.21 (m, 2H, CH), 0.97 (m, 2H, CH); ^13^C-NMR (CDCl_3_) δ 172.3, 161.6, 152.8, 144.0, 142.0, 126.1, 125.1, 124.9, 49.5, 29.3, 25.3, 24.2, 6.5; ν_max_ 2949, 2860, 1659, 1522, 1432, 1254, 1120, 824 cm^−1^; HRMS calcd for C_17_H_19_N_4_OS [M + H]^+^ 327.1280 found 327.1283.

*8-Cyclopropyl-2-(piperidin-1-yl)thiazolo[5,4-f]quinazolin-9(8H)-one* (**14f**): According to the general procedure, thiourea intermediate was obtained from **13** and morpholine (28.7 mg) after 30 min. at room temperature. After purification, **14f** was isolated as a colorless solid (93.6 mg, 92%), mp. 230–232 °C; ^1^H-NMR (DMSO-*d*_6_) δ 8.26 (s, 1H, H_7_), 7.92 (d, *J* = 8.7 Hz, 1H, H_4_), 7.62 (d, *J* = 8.7 Hz, 1H, H_5_), 3.79–3.75 (m, 4H, OCH_2_), 3.65–3.60 (m, 4H, NCH_2_), 3.33–3.28 (m, 1H, NCH), 1.08–1.00 (m, 4H, CH); ^13^C-NMR (DMSO-*d*_6_) δ 171.7, 160.5, 151.4, 145.6, 142.7, 125.4, 125.0, 124.8, 115.5, 65.5, 47.9, 29.2, 6.0; ν_max_ 3061, 2971, 2860, 1655, 1561, 1456, 1432, 1340, 1112, 823 cm^−1^; HRMS calcd for C_16_H_17_N_4_O_2_S [M + H]^+^ 329.1072 found 329.1060.

#### 3.2.4. General Procedure for the Synthesis of *N*-Alkyl-8-cyclopropyl-9-oxo-8,9-dihydrothiazolo[5,4-*f*]quinazoline-2-carboximidamides **17a**–**f** from **16**

In a sealed tube, a solution of carbonitrile derivative **16** (75 mg, 0.28 mmol) and the appropriate amine (2.8 mmol, 10 equiv.) in THF (1 mL) was heated under microwaves at 100 °C for 45 min under argon atmosphere. The solvent was removed under *vacuum* and the crude residue was adsorbed on Celite^®^ and purified by flash chromatography on silica gel with methanol/methylene chloride (0/100 to 2/98, *v/v*) as eluent to furnish the desired amidine compound.

*N-Benzyl-8-cyclopropyl-9-oxo-8,9-dihydrothiazolo[5,4-f]quinazoline-2-carboximidamide* (**17a**)*:* beige powder (105 mg, 93%), mp. 148–150 °C; ^1^H-NMR (DMSO-*d*_6_) δ 8.48 (s, 1H, H_7_), 8.43 (d, *J* = 9.0 Hz, 1H, H_4_), 7.84 (d, *J* = 9.0 Hz, 1H, H_5_), 7.49–7.23 (m, 5H, Ph), 6.99 (br s, 2H, NH_2_), 4.48 (s, 2H, CH_2_Ph), 3.39–3.33 (m, 1H, NCH), 1.14–1.01 (m, 4H, CH); ^13^C-NMR (CDCl_3_) δ 161.0, 152.1, 146.9, 146.8, 129.4, 129.0, 128.6, 127.5, 127.1, 126.1, 29.7, 6.6; ν_max_ 3456, 3328, 3187, 1699, 1653, 1604, 1586, 1448, 1342, 1311, 1091, 826, 738 cm^−1^; HRMS calcd for C_20_H_18_N_5_OS [M + H]^+^ 376.1232 found 376.1240.

*(Z)-8-Cyclopropyl-N′-[2-(dimethylamino)ethyl]-9-oxo-8,9-dihydrothiazolo[5,4-f]quinazoline-2-carboximidamide* (**17b**)*:* colorless solid (99 mg, 99%), mp. 168–170 °C; ^1^H-NMR (DMSO-*d*_6_) δ 8.46 (s, 1H, H_7_), 8.40 (d, *J* = 8.7 Hz, 1H, H_4_), 7.82 (d, *J* = 8.7 Hz, 1H, H_5_), 6.75 (br s, 2H, NH_2_), 3.38–3.33 (m, 1H, NCH), 3.31 (m, 2H, NCH_2_), 2.56 (m, 2H, NCH_2_), 2.23 (s, 6H, NCH_3_), 1.11–1.04 (m, 4H, CH); ν_max_ 3398, 3329, 3203, 2829, 1653, 1589, 1507, 1453, 1350, 1314, 829 cm^−1^; HRMS calcd for C_17_H_21_N_6_OS [M + H]^+^ 357.1498 found 357.1500.

*(Z)-8-Cyclopropyl-N′-(2-morpholinoethyl)-9-oxo-8,9-dihydrothiazolo[5,4-f]quinazoline-2-carboximidamide* (**17c**)*:* colorless solid (63 mg, 57%), mp. 154–156 °C; ^1^H-NMR (CDCl_3_) δ 8.32 (d, *J* = 8. 7 Hz, 1H, H_4_), 8.22 (s, 1H, H_7_), 7.80 (d, *J* = 8.7 Hz, 1H, H_5_), 3.76–3.74 (m, 4H, OCH_2_), 3.48 (t, *J* = 6.0 Hz, H1′), 3.37–3.32 (m, 1H, NCH), 2.76 (t, *J* = 6.0 Hz, H2′), 2.63–2.60 (m, 4H, NCH_2_), 1.30–1.23 (m, 2H, CH), 1.22–1.03 (m, 2H, CH), ^13^C-NMR (CDCl_3_) δ 169.2, 161.0, 153.0, 152.2, 146.9, 133.4, 129.5, 126.3, 116.4, 67.0, 58.3, 53.9, 42.6, 29.7, 6.7; ν_max_ 3480, 3374, 2966, 1642, 1316, 1110, 832, 521 cm^−1^; HRMS calcd for C_19_H_23_N_6_O_2_S [M + H]^+^ 399.1603 found 399.1604.

*(Z)-8-Cyclopropyl-9-oxo-N′-[2-(piperidin-1-yl)ethyl]-8,9-dihydrothiazolo[5,4-f]quinazoline-2-carboximidamide* (**17d**)*:* colorless solid (96 mg, 87%), mp. 180–182 °C; ^1^H-NMR (CDCl_3_) δ 8.33 (d, *J* = 8. 7 Hz, 1H, H_4_), 8.22 (s, 1H, H_7_), 7.80 (d, *J* = 8.7 Hz, 1H, H_5_), 3.52–3.48 (m, 2H, H1′), 3.37–3.33 (m, 1H, NCH), 2.70–2.66 (m, 1H, H2′), 2.52–2.50 (m, 4H, NCH), 1.65–1.59 (m, 4H, CH), 1.49–1.30 (m, 4H, CH), 1.30–1.26 (m, 2H, CH), 1.22–1.03 (m, 2H, CH), ^13^C-NMR (CDCl_3_) δ 169.8, 161.0, 152.3, 146.8, 133.5, 129.4, 126.1, 116.5, 54.9, 29.7, 26.1, 24.4, 6.7; ν_max_ 3463, 3325, 2933, 1650, 1588, 1345, 1040, 825, 568 cm^−1^; HRMS calcd for C_20_H_25_N_6_OS [M + H]^+^ 397.1811 found 397.1811.

*8-Cyclopropyl-2-[imino(morpholino)methyl]thiazolo[5,4-f]quinazolin-9(8H)-one* (**17e**): colorless solid (53.7 mg, 54%), mp. 184–186 °C ; ^1^H-NMR (DMSO-*d*_6_) δ 8.48 (s, 1H, H_7_), 8.45 (d, *J* = 9.0 Hz, 1H, H_4_), 8.83 (d, *J* = 9.0 Hz, 1H, H_5_), 3.71–3.68 (m, 4H, OCH_2_), 3.48–3.45 (m, 4H, NCH_2_), 3.39–3.33 (m, 1H, NCH), 1.12–1.07 (m, 4H, CH); ^13^C-NMR (DMSO-*d*_6_) δ 162.0, 160.8, 159.5, 151.6, 148.7, 147.1, 131.4, 129.7, 126.7, 116.0, 66.3, 46.9, 30.0, 6.4; ν_max_ 3279, 2955, 2854, 1660, 1586, 1496, 1436, 1347, 1109, 830 cm^−1^; HRMS calcd for C_17_H_25_N_5_O_2_S. [M + H]^+^ 356.1188 found 356.1190.

*8-Cyclopropyl-2-[imino(piperidin-1-yl)methyl]thiazolo[5,4-f]quinazolin-9(8H)-one* (**17f**): colorless solid (54.0 mg, 59%), mp. 204–206 °C ; ^1^H-NMR (DMSO-*d*_6_) δ 8.50 (s, 1H, H_7_), 8.48 (d, *J* = 8.7 Hz, 1H, H_4_), 7.18 (br s, 1H, NH), 7.87 (d, *J* = 8.7 Hz, 1H, H_5_), 3.43 (m, 4H, NCH_2_), 3.39–3.33 (m, 1H, NCH), 1.70–1.56 (m, 6H, CH_2_), 1.11–1.05 (m, 4H, CH); ν_max_ 3162, 2936, 2848, 1678, 1587, 1345, 829 cm^−1^; HRMS calcd for C_17_H_25_N_5_O_2_S. [M + H]^+^ 354.1389 found 354.1389.

### 3.3. In Vitro Kinase Preparation and Assays 

#### 3.3.1. Buffers

Buffer A: MgCl_2_ (10 mM), 1 mM ethylene glycol-bis(2-aminoethylether)-*N,N,N′,N′*-tetraacetic acid (EGTA), 1 mM dithiothreitol (DTT), 25 mM Tris-HCl pH 7.5, 50 μg heparin/mL.

Buffer B: β-Glycerophosphate (60 mM), 30 mM p-nitrophenylphosphate, 25 mM 3-(*N*-morpholino)propanesulfonic acid (Mops) (pH 7.2), 5 mM EGTA, 15 mM MgCl_2_, 1 mM DTT, 0.1mM sodium vanadate.

#### 3.3.2. Kinase Preparations and Assays

Kinase activities were assayed in triplicates in buffer A or B, for 30 min. at 30 °C, at a final adenosine triphosphate (ATP) concentration of 15 μM. Blank values were substracted and activities expressed in % of the maximal activity, *i.e.*, in the absence of inhibitors. Controls were performed with appropriate dilutions of dimethylsulfoxide (DMSO). IC_50_ values were calculated from dose-response curves established by Sigma-Plots. The GSK-3, CK1, DYRK1A and CLK1 peptide substrates were obtained from Proteogenix (Oberhausbergen, France) [43,44].

*CDK5/p25.* (Human, recombinant) was prepared as previously described [45]. Its kinase activity was assayed in buffer A, with 1 mg of histone H1/mL, in the presence of 15 μM [γ-^33^P] ATP (3000 Ci/mmol; 10 mCi/mL) in a final volume of 30 μL. After 30 min incubation at 30 °C, 25 μL aliquots of supernatant were spotted onto sheets of Whatman P81 phosphocellulose paper, and 20 s later, the filters were washed eight times (for at least 5 min each time) in a solution of 10 mL phosphoric acid/L of water. The wet filters were counted in the presence of 1 mL ACS (Amersham) scintillation fluid.

*GSK-3α/β.* (Porcine brain, native) was assayed, as described for CDK5/p25 but in buffer A and using a GSK-3 specific substrate (GS-1: YRRAAVPPSPSLSRHSSPHQpSEDEEE) (pS stands for phosphorylated serine) [46].

*CK1δ/ε.* (Porcine brain, native) was assayed as described for CDK5/p25 but using the CK1-specific peptide substrate RRKHAAIGpSAYSITA [47].

*DYRK1A.* (Rat, recombinant, expressed in *E. coli* as a glutathione transferase (GST) fusion protein) was purified by affinity chromatography on glutathione-agarose and assayed, as described for CDK5/p25 using Woodtide (KKISGRLSPIMTEQ) (1.5 µg/assay) as a substrate.

*CLK1.* (Human, recombinant, expressed in *E. coli* as GST fusion protein) was assayed in buffer A (+0.15 mg BSA/mL) with RS peptide (GRSRSRSRSRSR) (1 μg/assay).

## 4. Conclusions

A library of thirty eight novel thiazolo[5,4-*f*]quinazoline derivatives (series **8**, **10**, **14** and **17**) has been prepared, using microwave-assisted technology. An efficient multistep synthesis of 6-amino-3-cyclopropylquinazolin-4(3*H*)-one (**3**) was developed and optimized to the multigram scale, affording the synthesis of large quantities of a versatile and efficient precursor for the various target molecules in this study. The synthetic routes to these variously 8-aminoaryl thiazolo[5,4-*f*]quinazolin-9(8*H*)-ones, incited us to re- explore some aspects of the Hügershoff reaction involving a first bromination on thiocarbonyl group of a thiourea, followed by intramolecular electrophilic aromatic substitution. Whilst the second route was envisioned via a CuI catalyzed ligand-free intramolecular C-S bond formation allowing the target 2-substituted benzothiazoles from thiourea precursors. The inhibitory potency of the final products against five kinases involved in AD was evaluated. Our study demonstrates that molecules of the **8**, **10** and **14** series described in this paper are not pertinent for the development of kinases inhibitors. The most active compounds are carboximidamides analogues of the target compounds of this study. They showed submicromolar IC_50_ values for CLK1, DYRK1A and GSK-3α/β over the other tested enzymes, suggesting that only chemical functions derived from a carbonitrile or a carbonyl carbon may generate affinity and specificity for a set of specific kinases.

## Figures and Tables

**Table 1:** Chemical structures and yields obtained for the synthesis of series **7a**–**l**.

-R ^a^	Compound	Method	Time (h)	Yield ^b^ (%)
Ph	**7a**	A/B	1/2	84/80
4-Cl-C_6_H_4_	**7b**	A	1	91
4-F-C_6_H_4_	**7c**	B	2	90
4-CF_3_-C_6_H_4_	**7d**	B^c^	1	84
4-NO_2_-C_6_H_4_	**7e**	A	0.5	74
2,4-diCl-C_6_H_3_	**7f**	B	1	89
3-Py	**7g**	B^c^	2	89
4-Me-C_6_H_4_	**7h**	A	2	89
4-OMe-C_6_H_4_	**7i**	A	2	87
4-NMe_2_-C_6_H_4_	**7j**	B	10 min	87
2,4-diOMe-C_6_H_3_	**7k**	B	5 min	97
3,4-diOMe-C_6_H_3_	**7l**	B	5 min	88

^a^ This list of anilines was established, based on the Topliss schemes [42], in order to get the most potent compounds as early as possible; ^b^ Isolated yield; ^c^ heated for 1 h at 40 °C under microwaves.

**Table 2:** Chemical structures and yields obtained for the synthesis of series **8a**–**g** via the Hügershoff reaction.

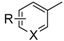	Compound	Time (h)	Yield ^a^ (%)
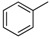	**8a**	0.5	10 ^b,c^
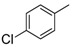	**8b**	1	- ^d^
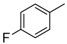	**8c**	1	45 ^b^
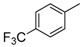	**8d**	1	79 ^b^
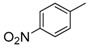	**8e**	4	86 ^b^
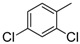	**8f**	1.5	90
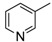	**8g**	4	80

^a^ Isolated yield; ^b^ Traces of compounds of the **9** series were detected but not purified; ^c^ In this case only compound **10a** was isolated in a 30% yield; ^d^ Not isolated.

**Table 3:** Experimental condition optimization for the intramolecular metal-catalyzed cyclization of **12**.

Entry	CuI (mol %)	Ligand ^a^	Cs_2_CO_3_ (Equiv)	Conversion ^b^ (%) (Isolated Yield)
1	10	20	2	100
2	10	-	2	100 (80)
3	5	-	2	80 ^b^
4	10	-	-	65 ^b^
5	-	-	2	- ^c^

^a^ 1,10-phenanthroline; ^b^ Reaction conversion was determined by ^1^H NMR analysis; ^c^ Traces.

**Table 4:** Chemical structures and yields obtained for the sequential MCR-synthesis of **8a**, **8c** and **8h**–**m** via Cu catalysed intramolecular cyclization.

-R^1^	-R^2^	Compound	Time ^a^ (h)	Yield ^b^ (%)
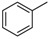	H	**8a**	1	80
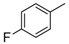	H	**8c**	2	96
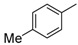	H	**8h**	1	89
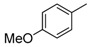	H	**8i**	0.5	88
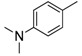	H	**8j**	0.5	87
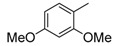	H	**8k**	0.5	86
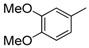	H	**8l**	0.5	90
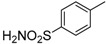	H	**8m**	12	77

^a^ Time of the conversion of **13** into the corresponding thioureas (step 1); ^b^ Isolated yield.

**Table 5:** Chemical structures and yields obtained via the sequential MCR synthesis of **14a**–**14f**.

-R^1^	-R^2^	Compound	Time ^a^ (h)	Yield ^b^ (%)
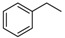	H	**14a**	0.5	80
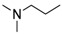	H	**14b**	0.5	96
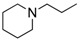	H	**14c**	0.5	89
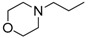	H	**14d**	0.5	88
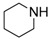		**14e**	0.5	87
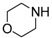		**14f**	0.5	86

^a^ Time of the conversion of **13** into the corresponding thioureas (step 1); ^b^ Isolated yield.

**Table 6:** Chemical structures and yields obtained for the synthesis of 1**7a**–**f**.

-R^1^	-R^2^	Compound	Time ^a^ (min)	Yield ^b^ (%)
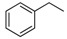	H	**17a**	45	82
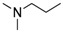	H	**17b**	45	99
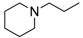	H	**17c**	45	87
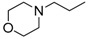	H	**17d**	45	57
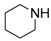		**17e**	90	60
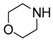		**17f**	180	54

^a^ Time of the conversion of **16** into the corresponding carboximidamides; ^b^ Isolated yield.

**Table 7:** Kinase inhibitory activity ^a,b,c^ of the thiazolo[5,4-*f*]quinazoline series (**7a**–**l**, **8a**–**m**, **10a** and **10l**, **14a**–**f** and **17a**–**f**).

Compounds	CDK5/p25	CK1δ/ε	CLK1	DYRK1A	GSK-3α/β
**7a–l**	>10	>10	>10	>10	≥10
**8a**	>10	>10	**3.4**	>10	≥10
**8c–f**	>10	>10	>10	>10	≥10
**8g**	>10	>10	**1.7**	>10	≥10
**8h–i**	>10	>10	>10	>10	≥10
**8j**	>10	>10	**1.3**	**2.0**	**7.3**
**8k–m**	>10	>10	>10	>10	≥10
**10a**	>10	>10	**5.2**	**4.8**	≥10
**10l**	>10	>10	>10	>10	≥10
**14a**	>10	>10	**3.4**	>10	>10
**14b**	>10	>10	>10	>10	≥10
**14c**	>10	>10	>10	>10	≥10
**14d**	>10	>10	**8.1**	**8.2**	≥10
**14e**	>10	>10	**5.3**	>10	≥10
**14f**	>10	>10	>10	>10	≥10
**17a**	>10	>10	1.0	**0.67**	**0.4**
**17b**	>10	>10	**0.29**	4.4	1.1
**17c**	>10	>10	**2.3**	**3.2**	**2.8**
**17d**	>10	>10	**2.1**	**2.9**	**3.9**
**17e**	>10	1.9	**0.38**	**0.14**	**0.23**
**17f**	>10	>10	**0.61**	**0.82**	**0.49**
**Harmine**	>10	1.5	**0.026**	**0.029**	>10

^a^ IC_50_ values are reported in μM. The most significant results are presented in bold; ^b^ Kinases activities were assayed in triplicate; ^c^ Typically, the standard deviation of single data points was below 10%.

## Supplementary Materials

Supplementary materials can be accessed at: http://www.mdpi.com/1420-3049/21/6/794/s1.

## Abbreviations

The following abbreviations are used in this manuscript:

ATP	Adenosine triphosphate
DBU	1,8-diazabicyclo[5.4.0]undec-7-ene
CMGC group	Group of kinases including Cyclin-dependent kinases (CDKs), Mitogen-activated protein kinases (MAP kinases), Glycogen synthase kinases (GSK) and Cyclin dependent kinases (CDK-like kinases)
DMF	*N,N-*Dimethylformamide
DMFDMA	*N,N-*Dimethylformamide dimethyl acetal
MCR	Multi-component reaction
MTDL	Multi-target-directed ligand
NBS	*N*-Bromosuccinimide
