# Supplementary material for: Synthesis of Bioactive 2-(Arylamino)thiazolo[5,4-f]-quinazolin-9-ones via the Hügershoff Reaction or Cu- Catalyzed Intramolecular C-S Bond Formation
Source: Molecules. 2016 Jun 18;21(6):794. doi: 10.3390/molecules21060794 (PMC6272913; doi:10.3390/molecules21060794)

Supplementary Materials: Synthesis of Bioactive 2-(Arylamino)thiazolo[5,4-*f*]quinazolin-9-ones via Hügershoff Reaction or Cu Catalyzed Intramolecular C-S Bond Formation

Damien Hédou, Carole Dubouilh-Benard, Nadège Loaëc, Laurent Meijer, Corinne Fruit and Thierry Besson

^{1}H and ^{13}C-NMR Spectra of Compounds 2–6, 7a–l, 8a–m, 10a and 10l, 11–13, 14a-f, 15, 16 and 17a–d

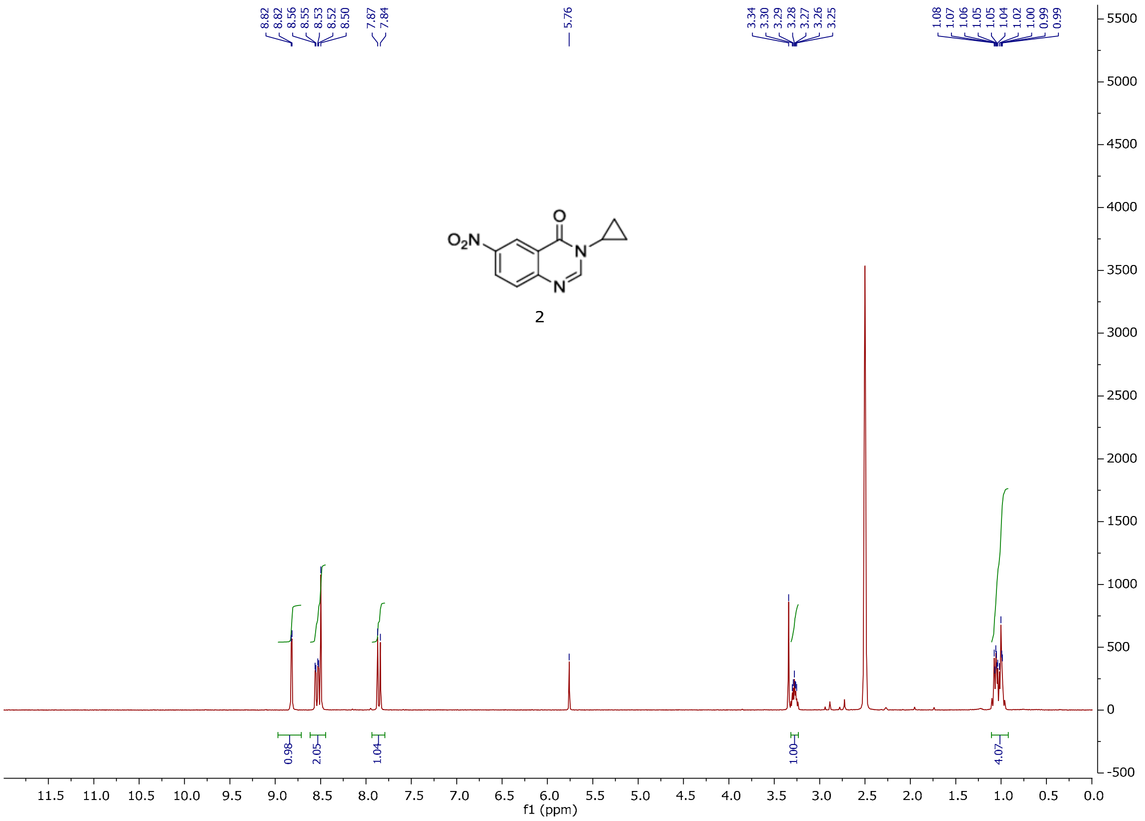

Figure S1. ^{1}H-NMR Compound **2**.

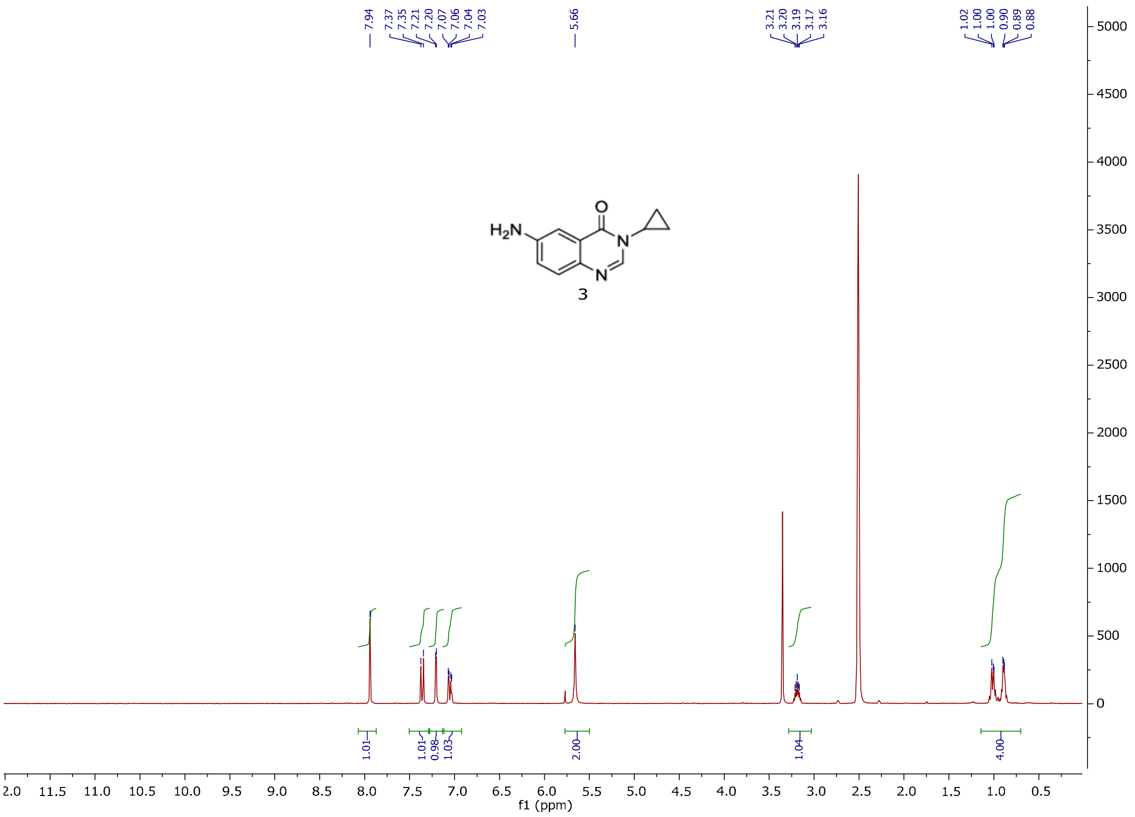

Figure S2. ^{1}H-NMR and ^{13}C-NMR Compound **3**.

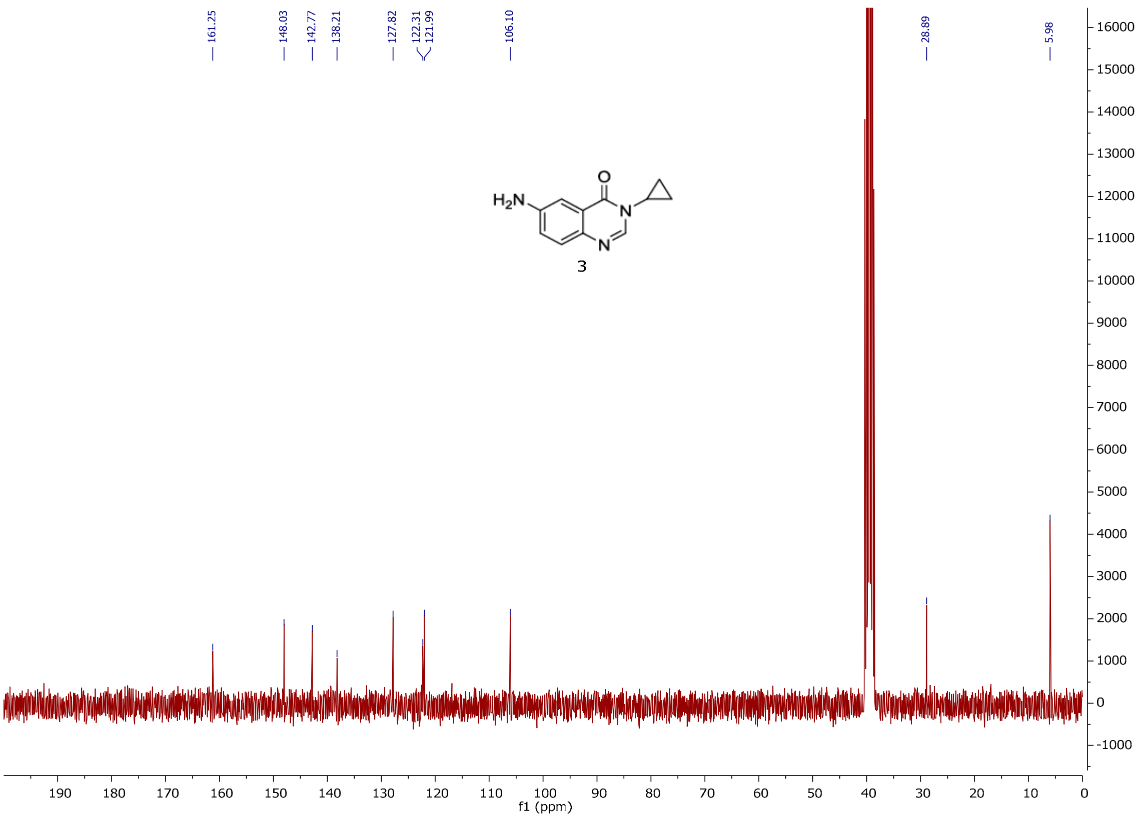

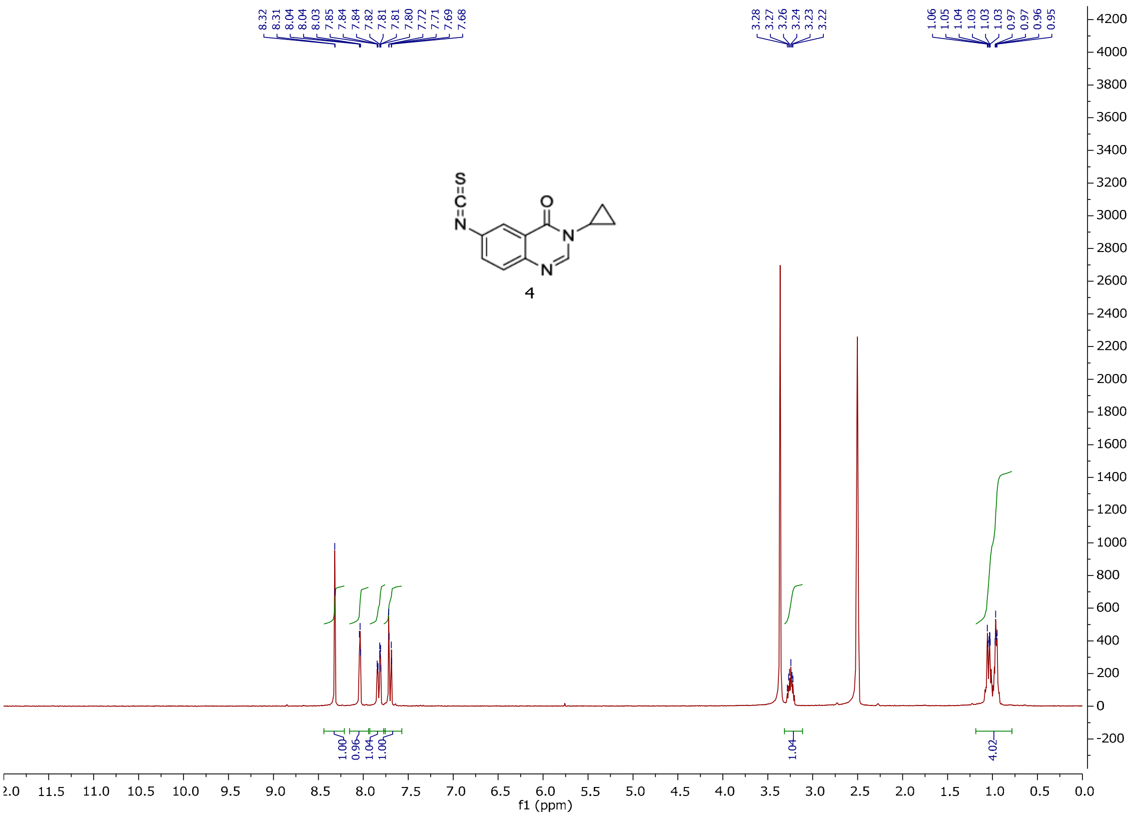

Figure S3. ^{1}H-NMR and ^{13}C-NMR Compound **4**.

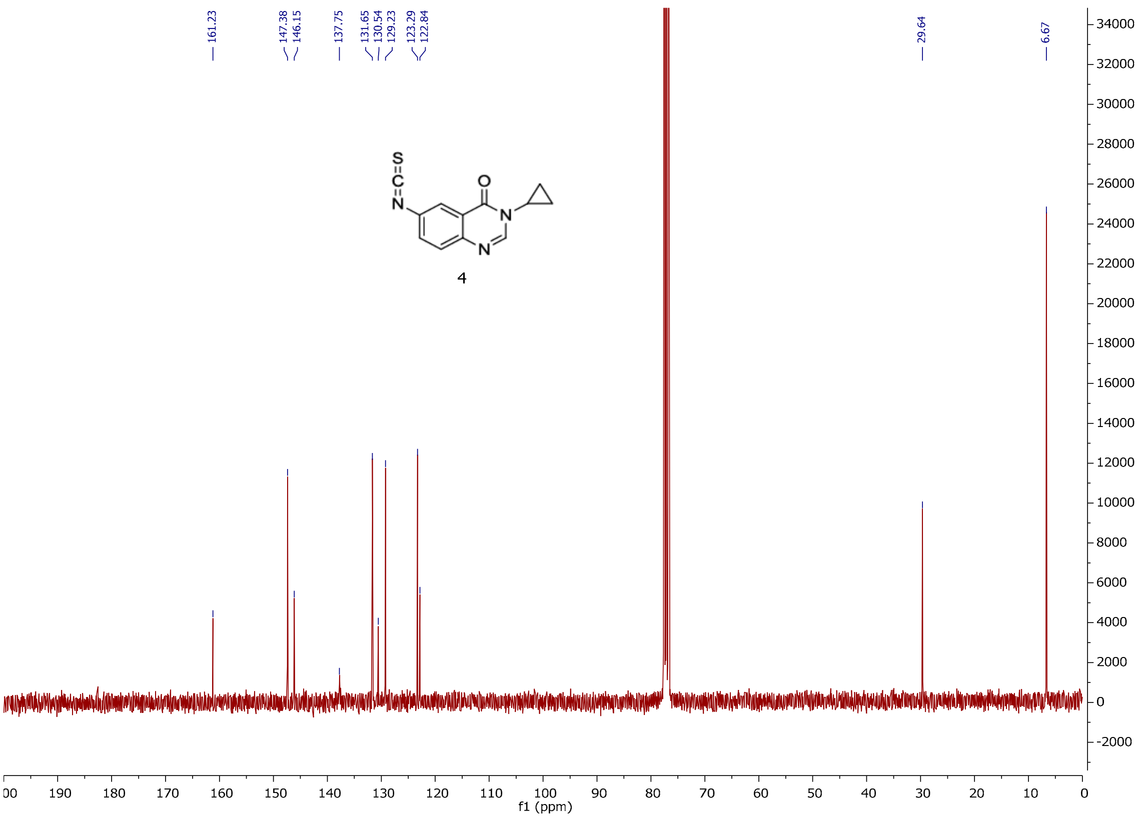

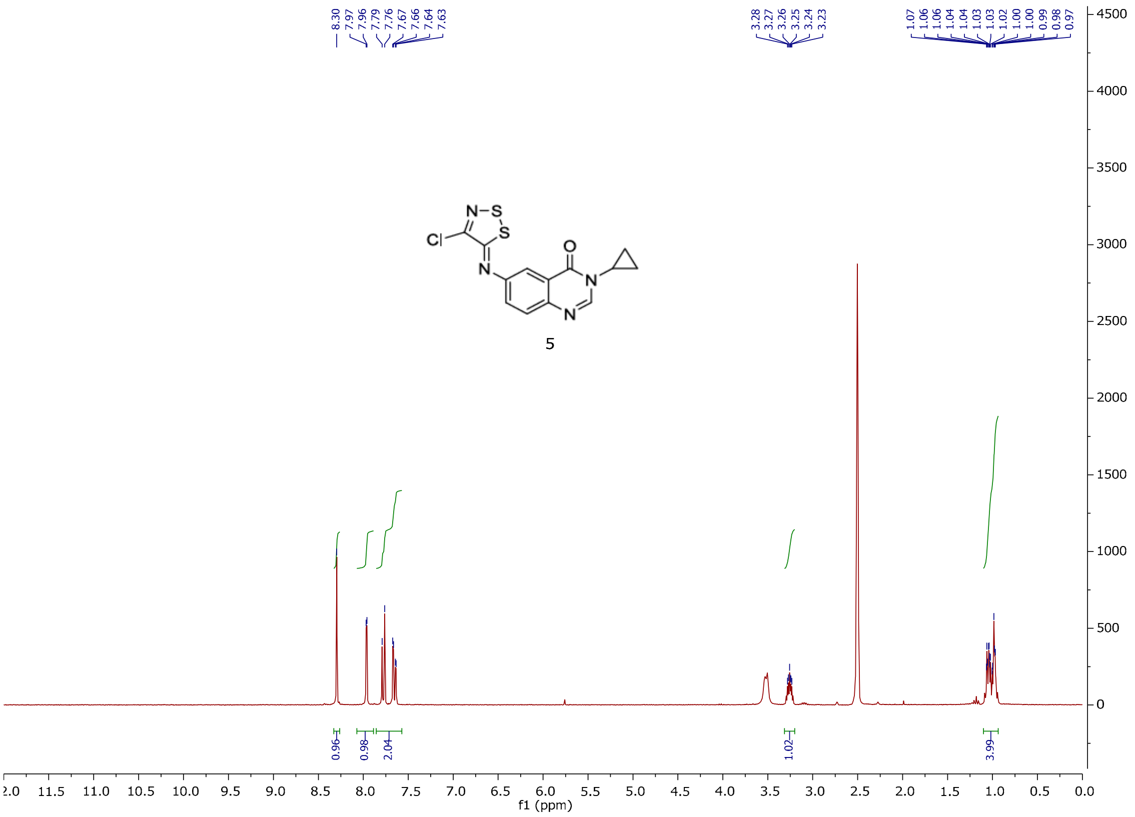

Figure S4. ^{1}H-NMR and ^{13}C-NMR Compound **5**.

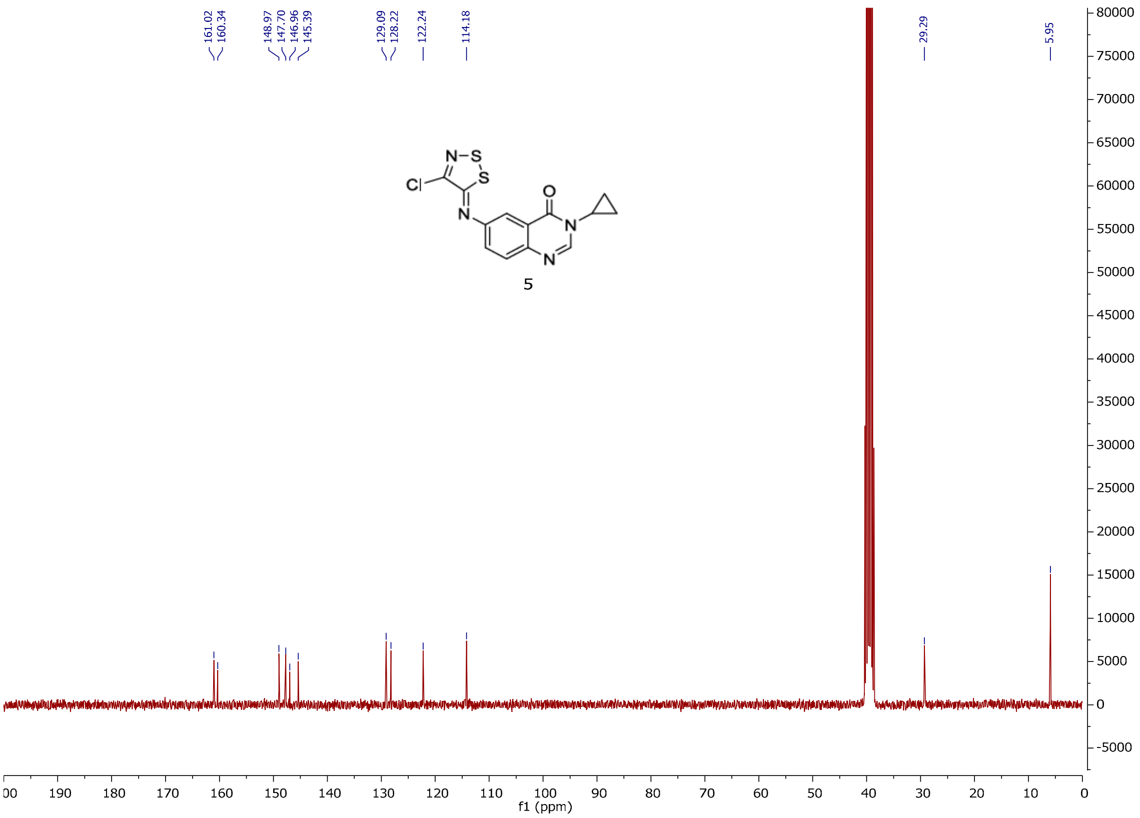

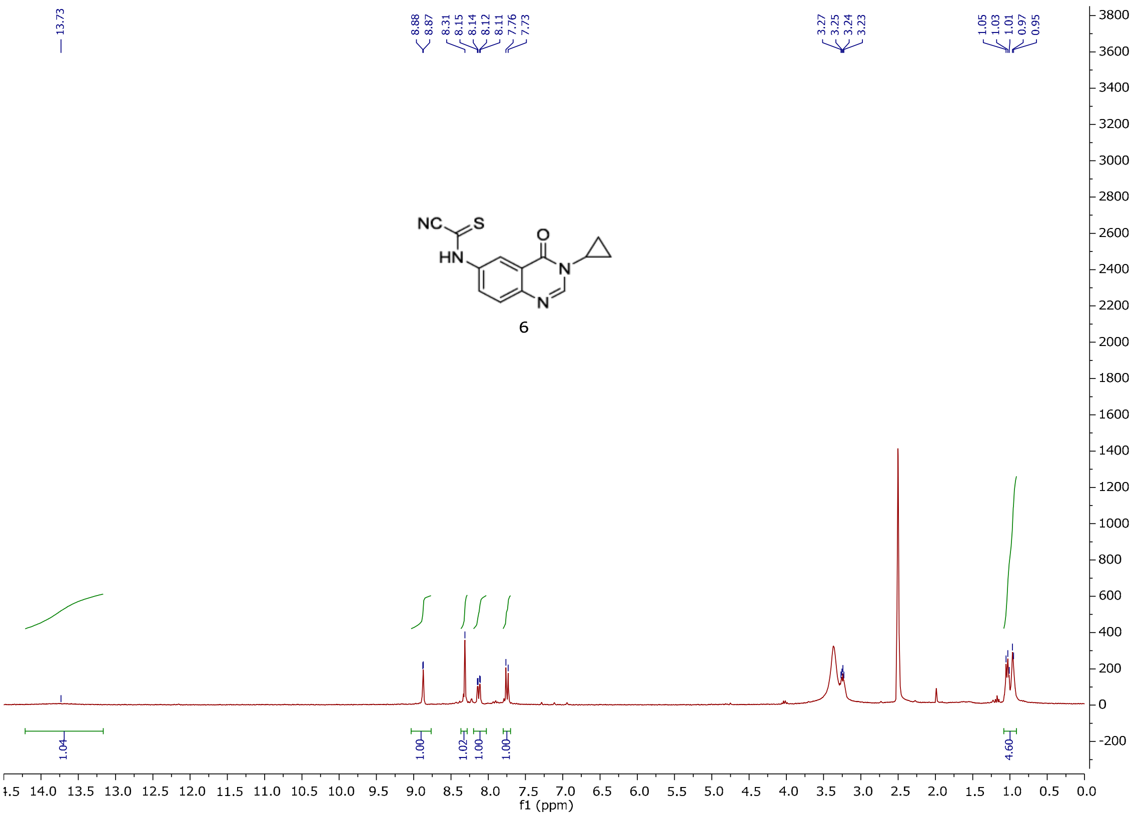

Figure S5. ^{1}H-NMR and ^{13}C-NMR Compound **6**

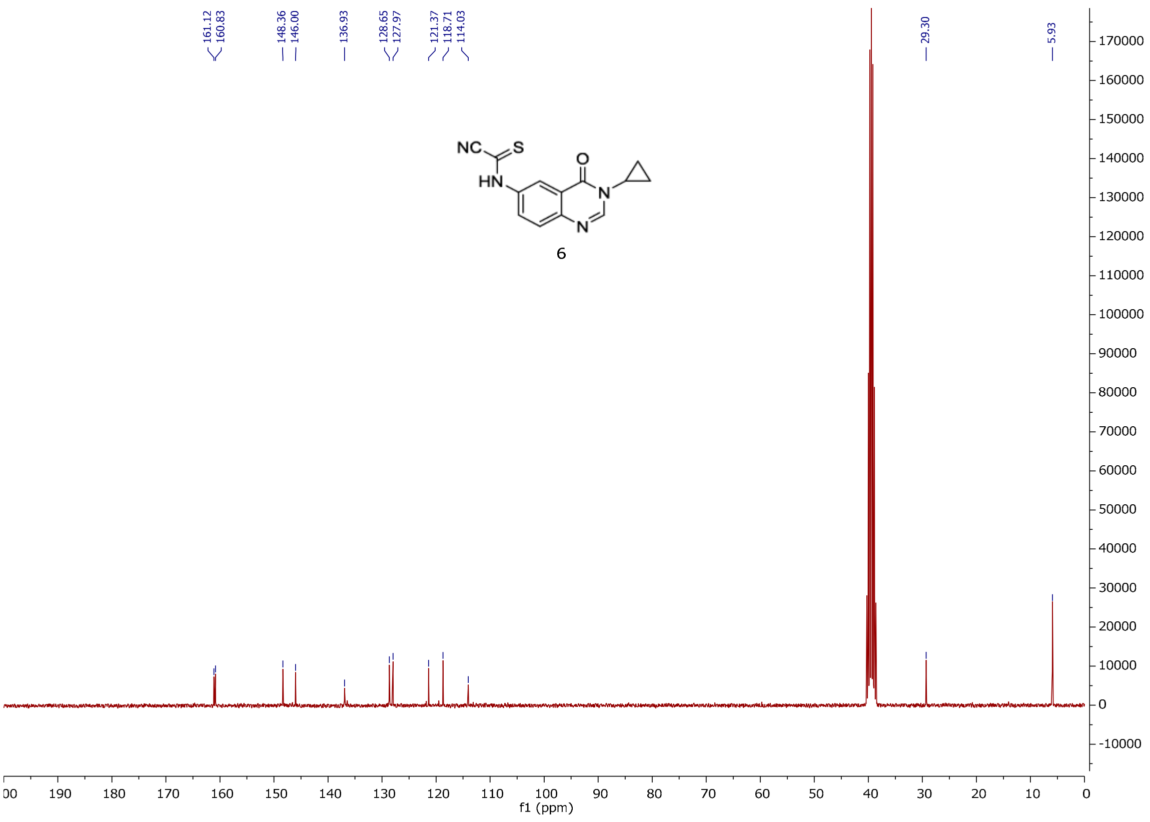

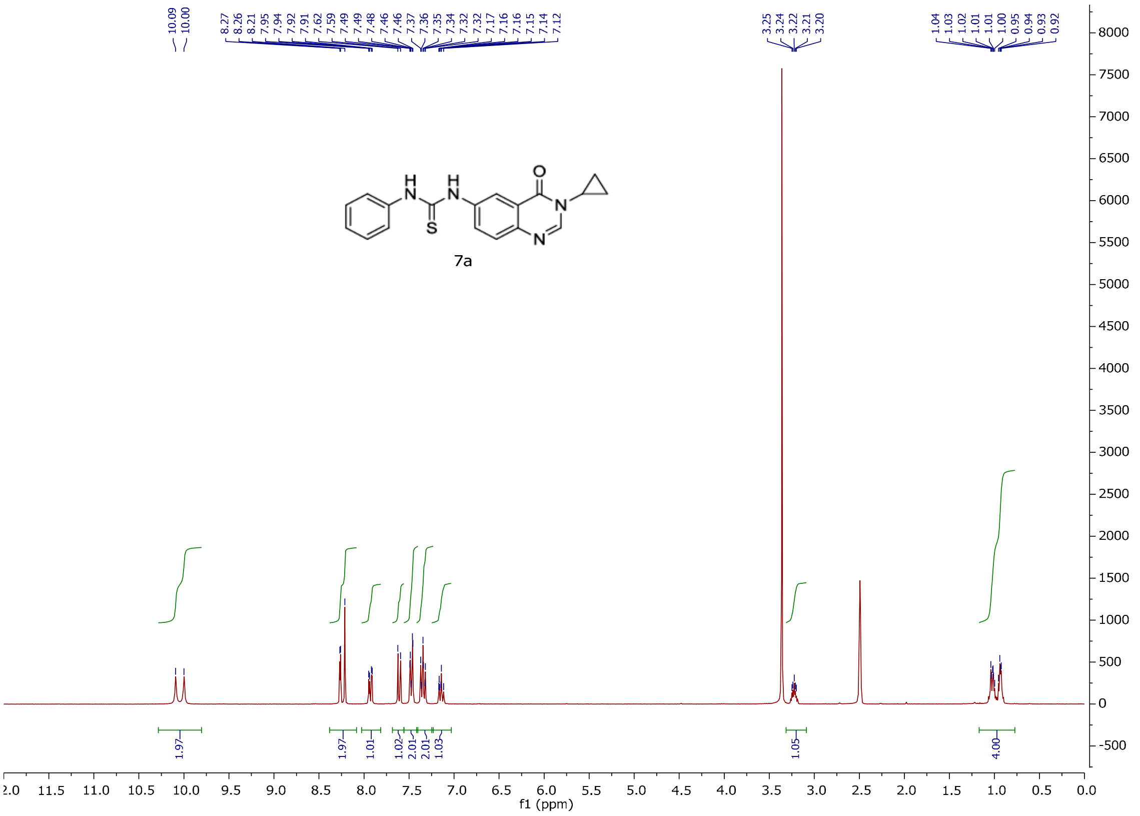

Figure S6. ^{1}H-NMR and ^{13}C-NMR Compound **7a**

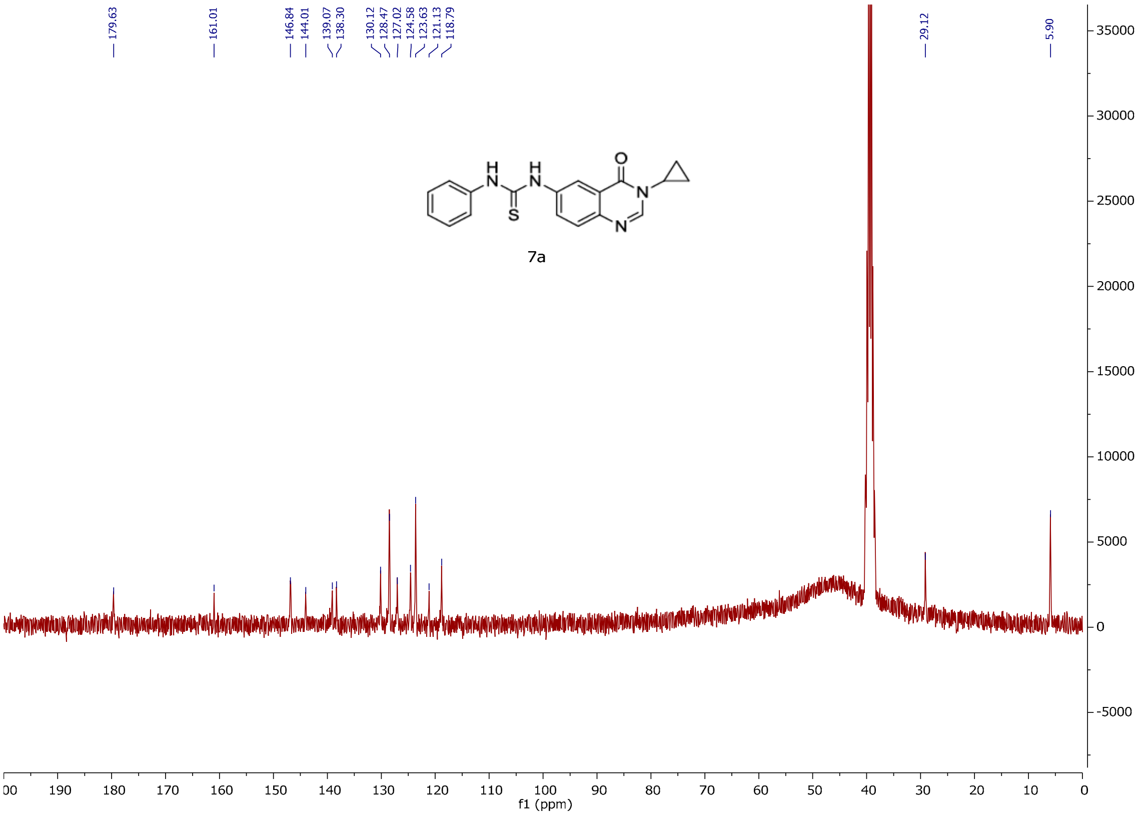

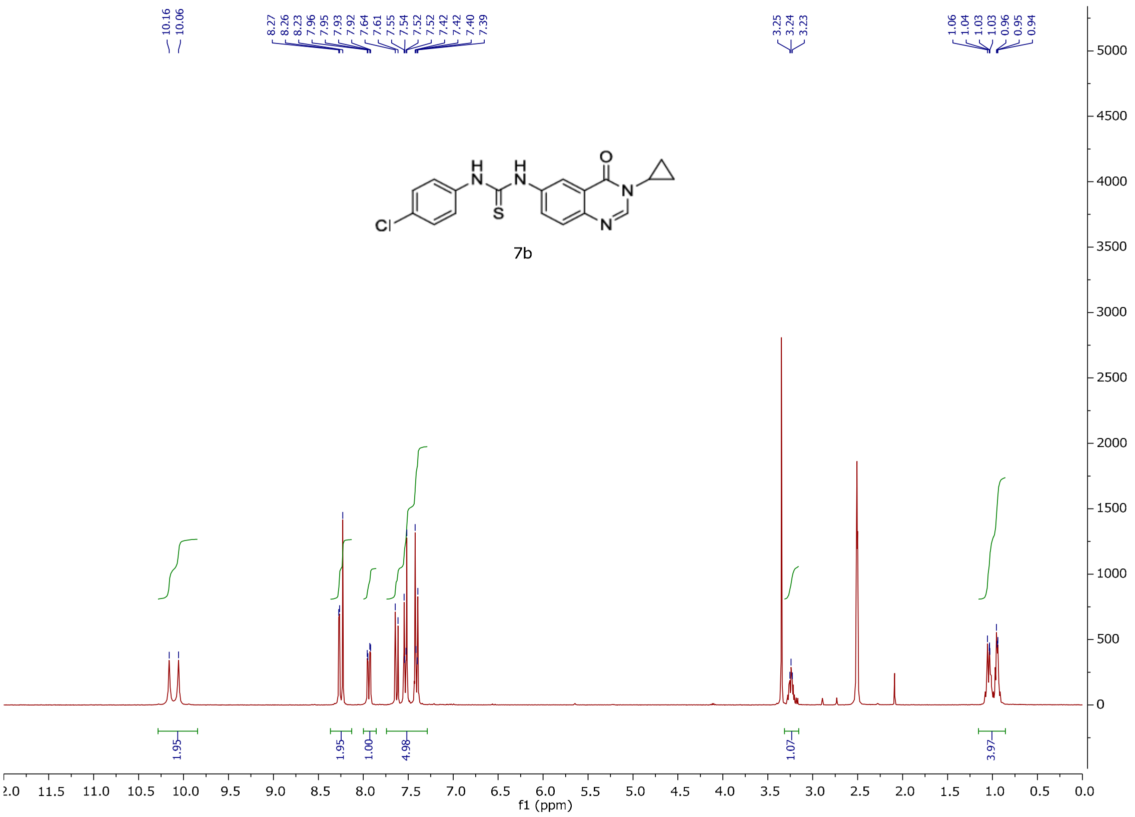

Figure S7. ^{1}H-NMR and ^{13}C-NMR Compound **7b**

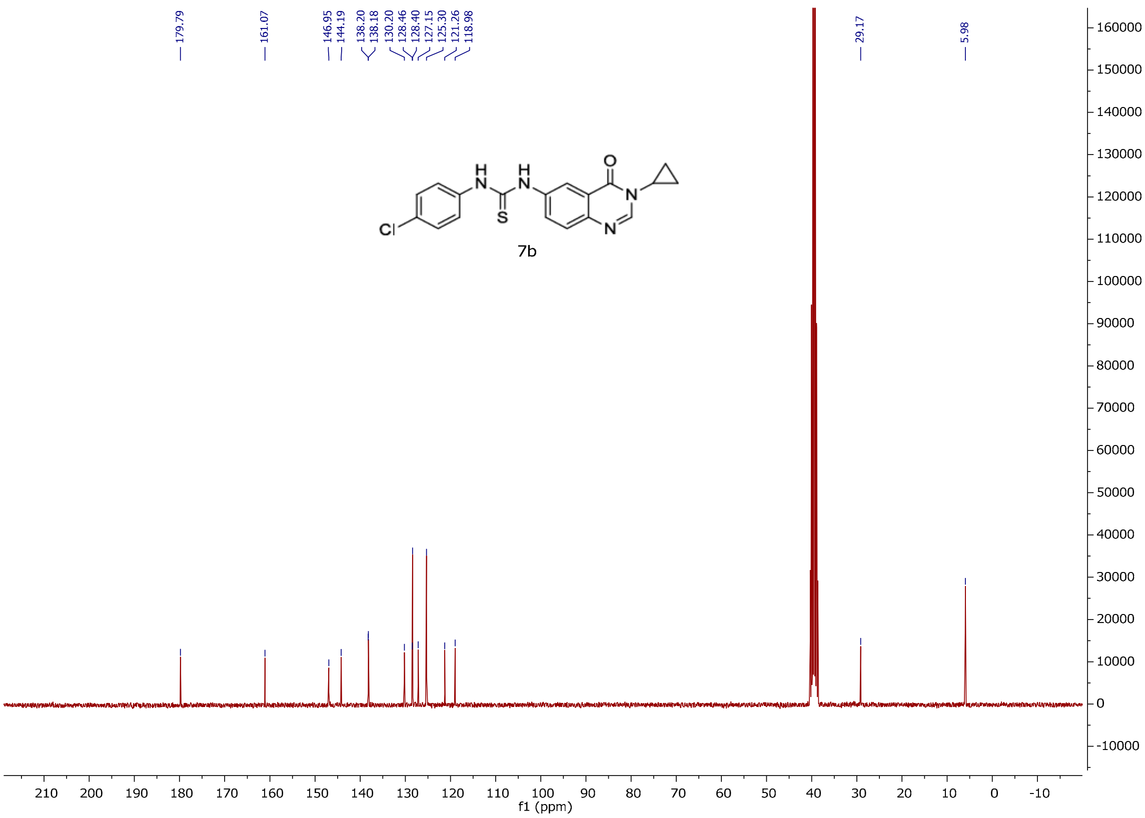

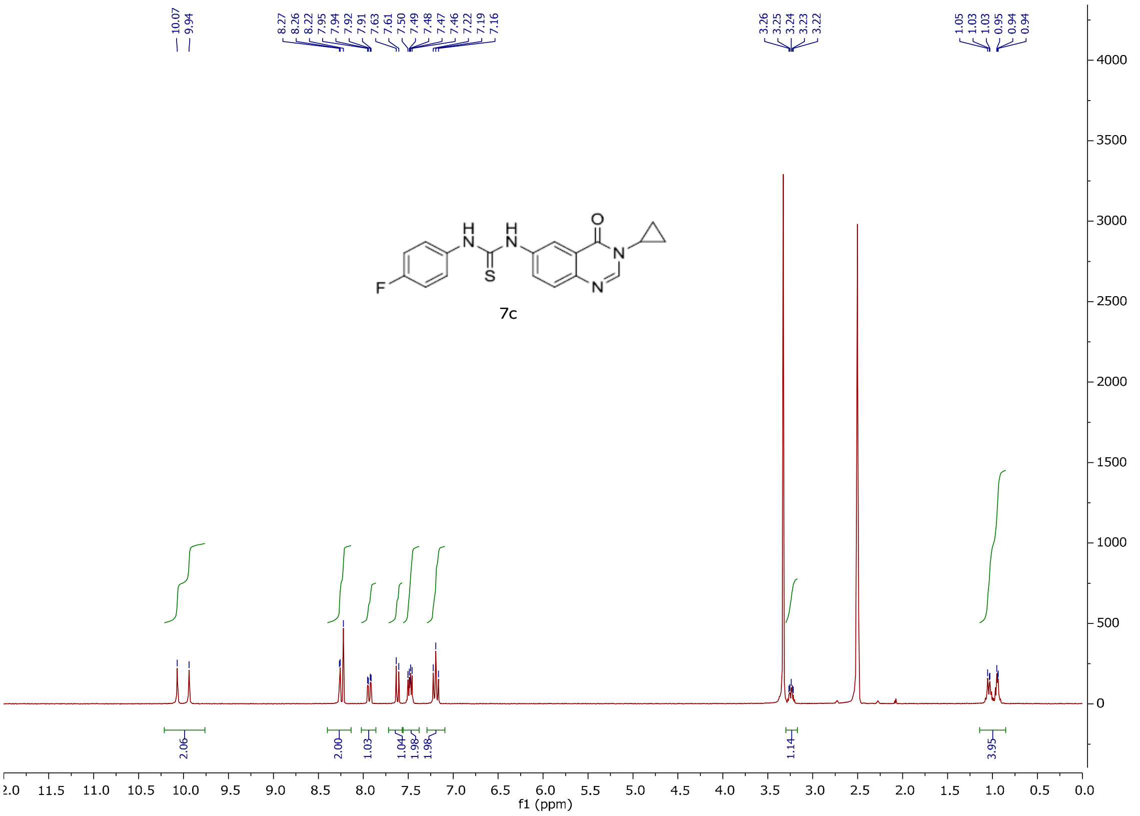

Figure S8. ^{1}H-NMR and ^{13}C-NMR Compound **7c**

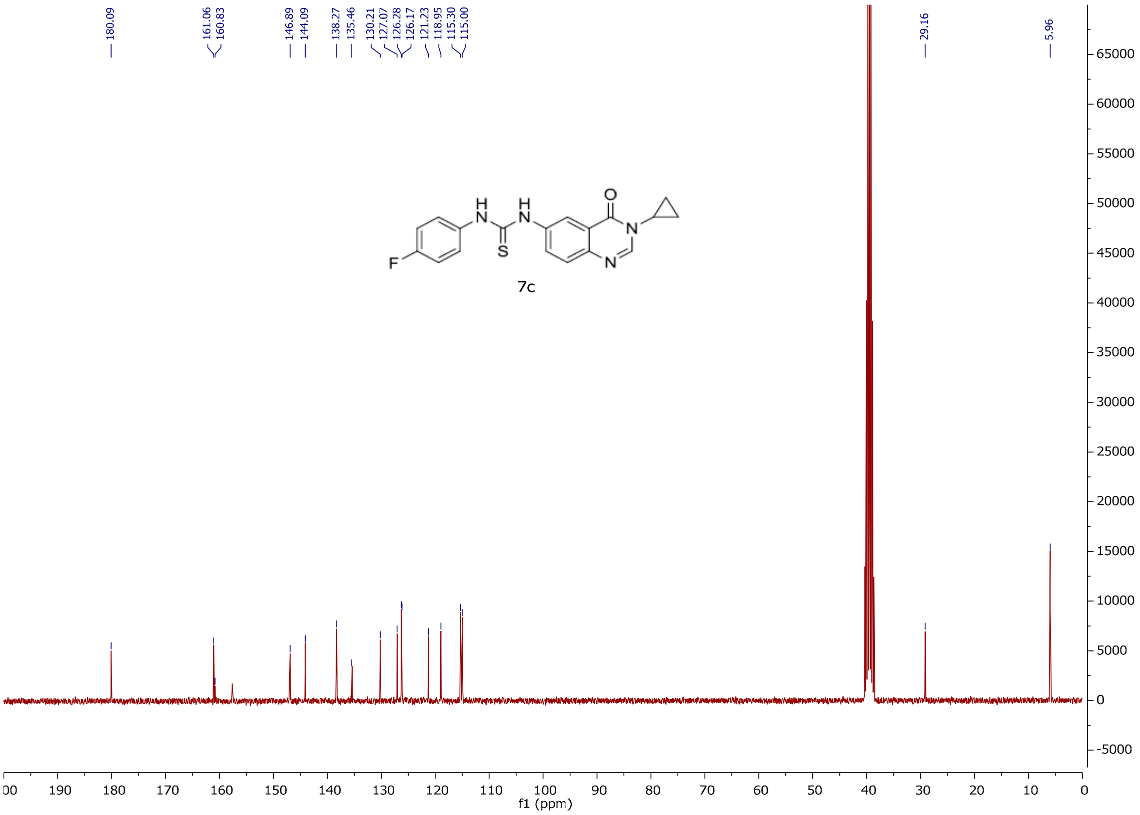

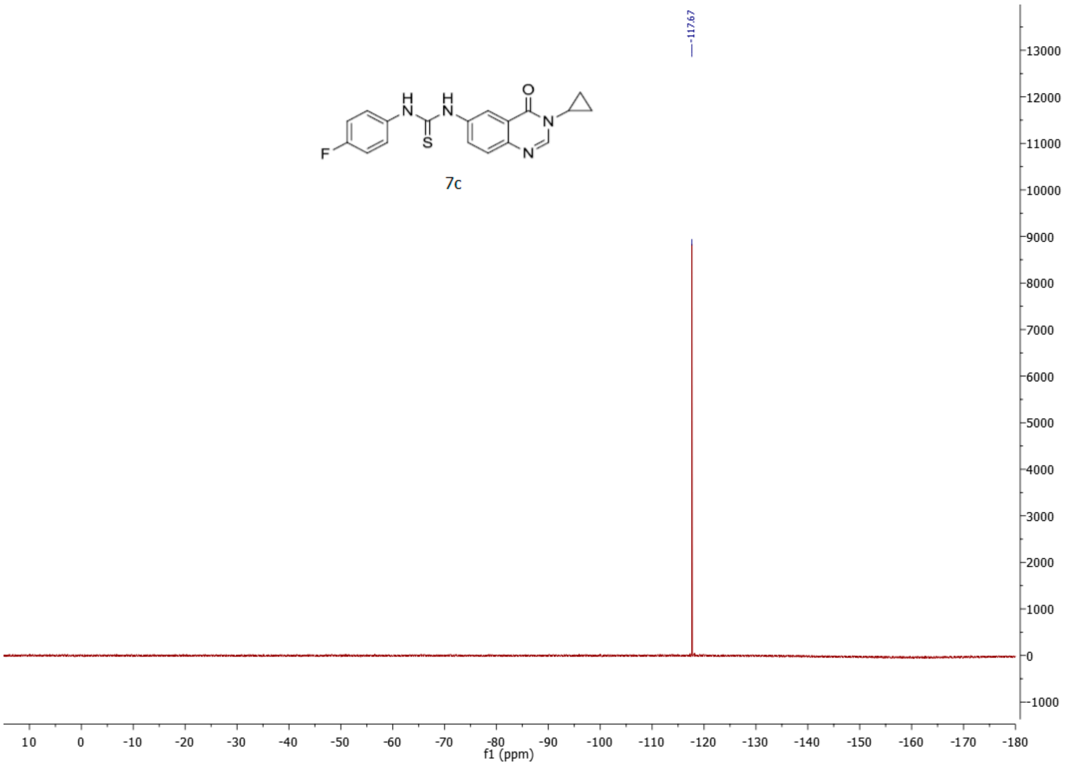

Figure S9. ^{19}F-NMR Compound **7c**

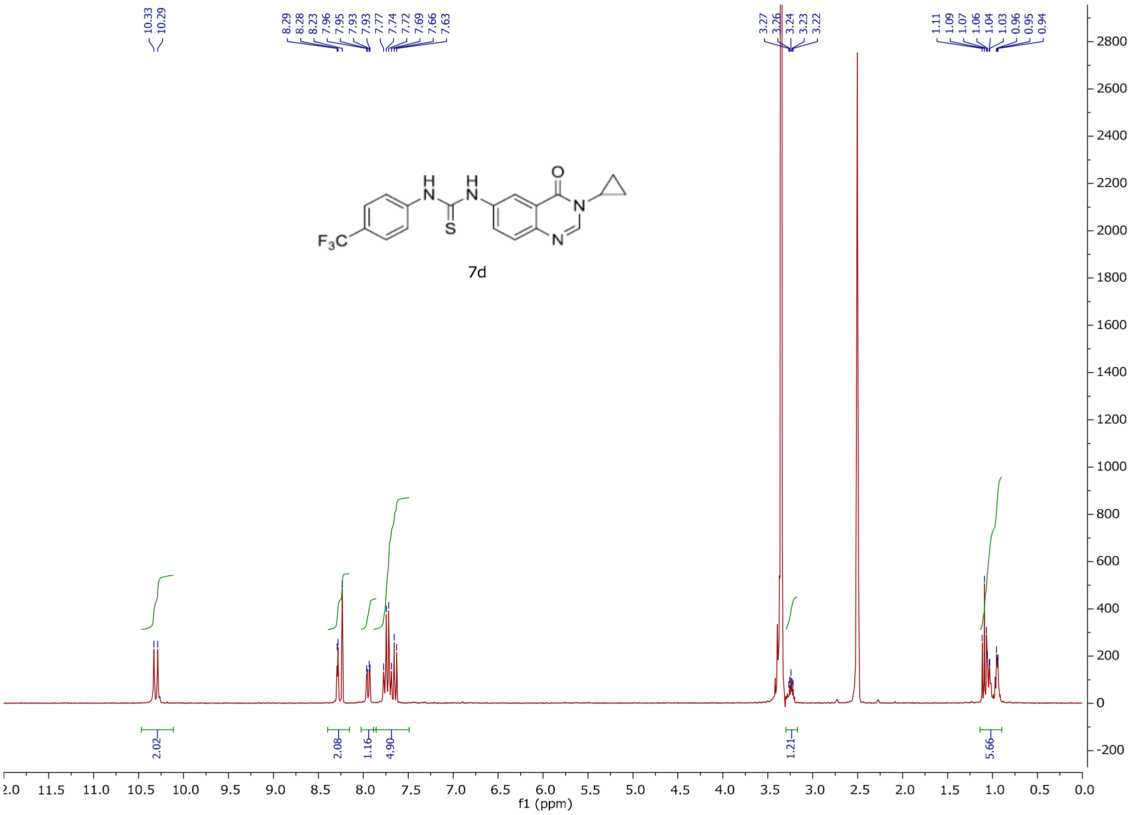

Figure S10. ^{1}H-NMR and ^{13}C-NMR Compound **7d**

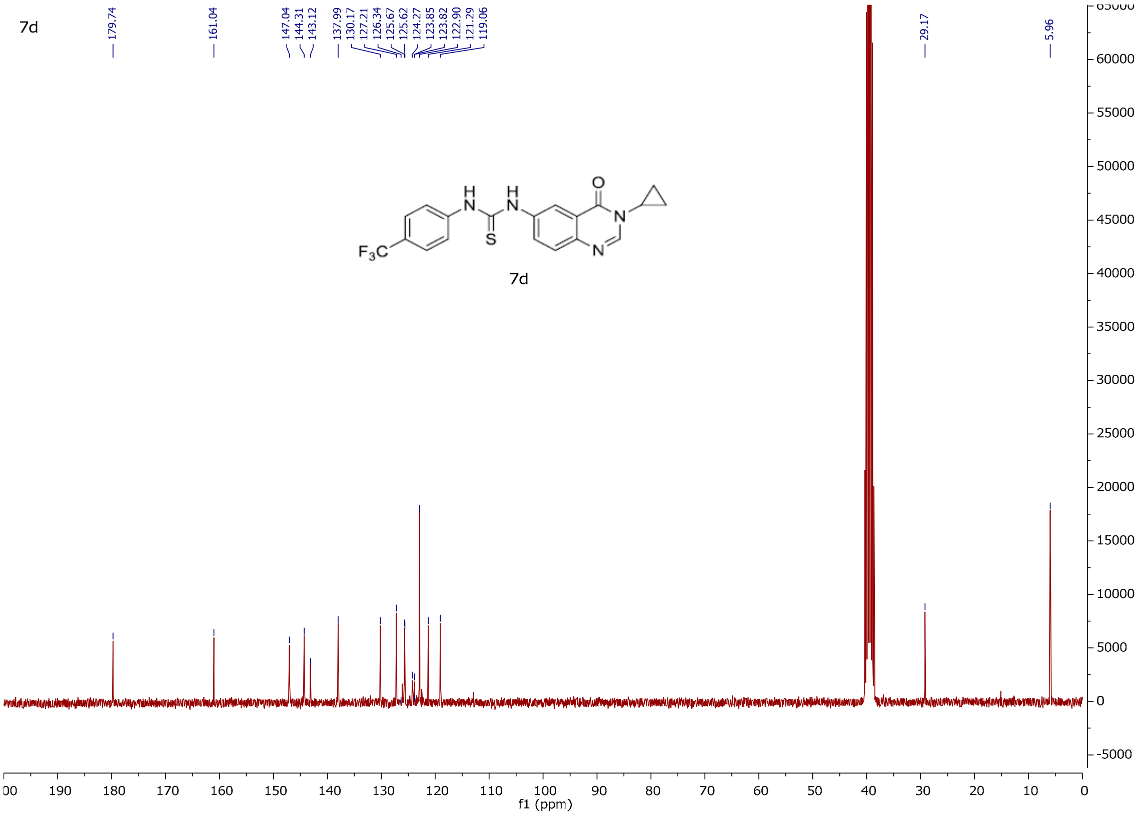

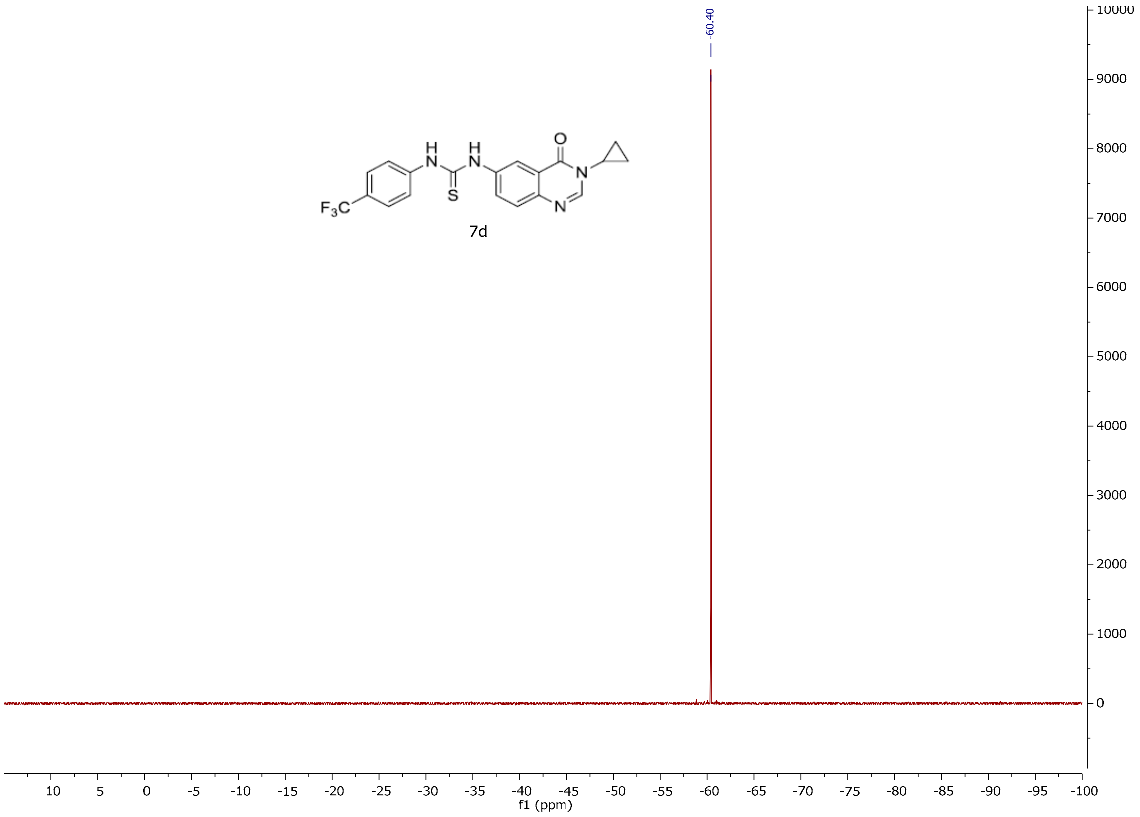

Figure S11. ^{19}F-NMR Compound **7d**

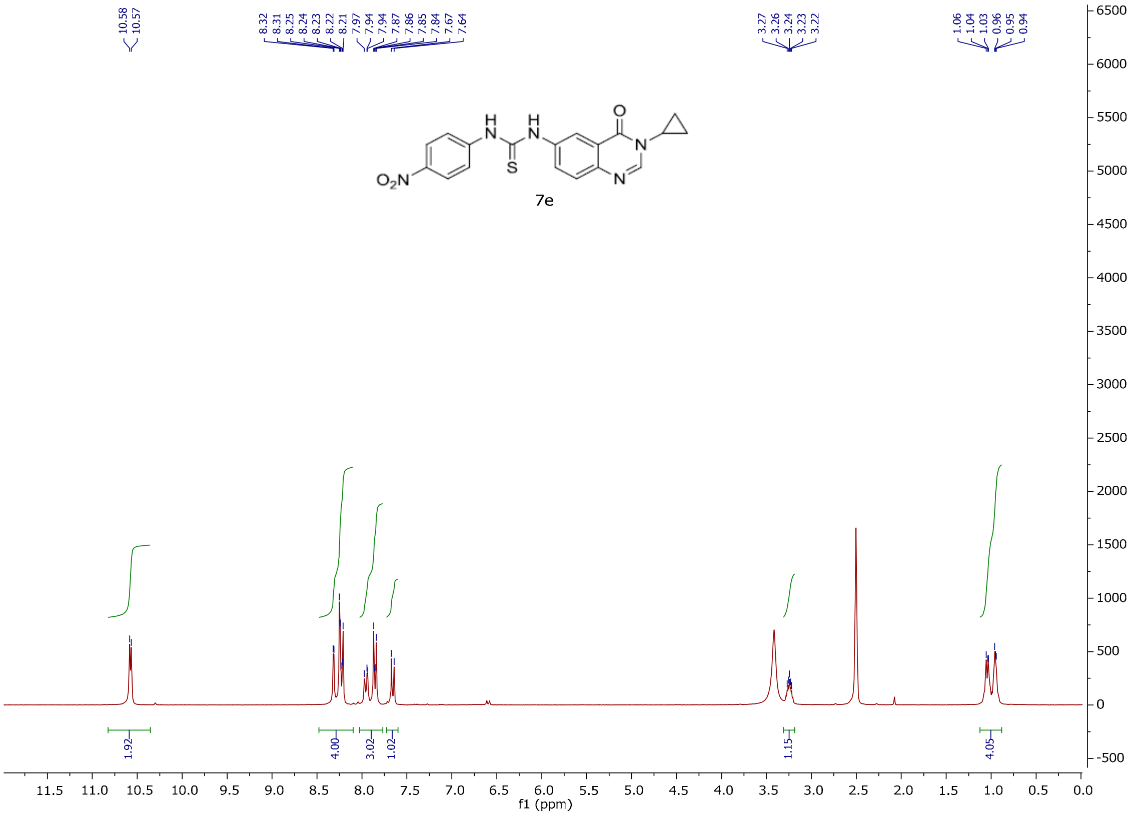

Figure S12. ^{1}H-NMR and ^{13}C-NMR Compound **7e**

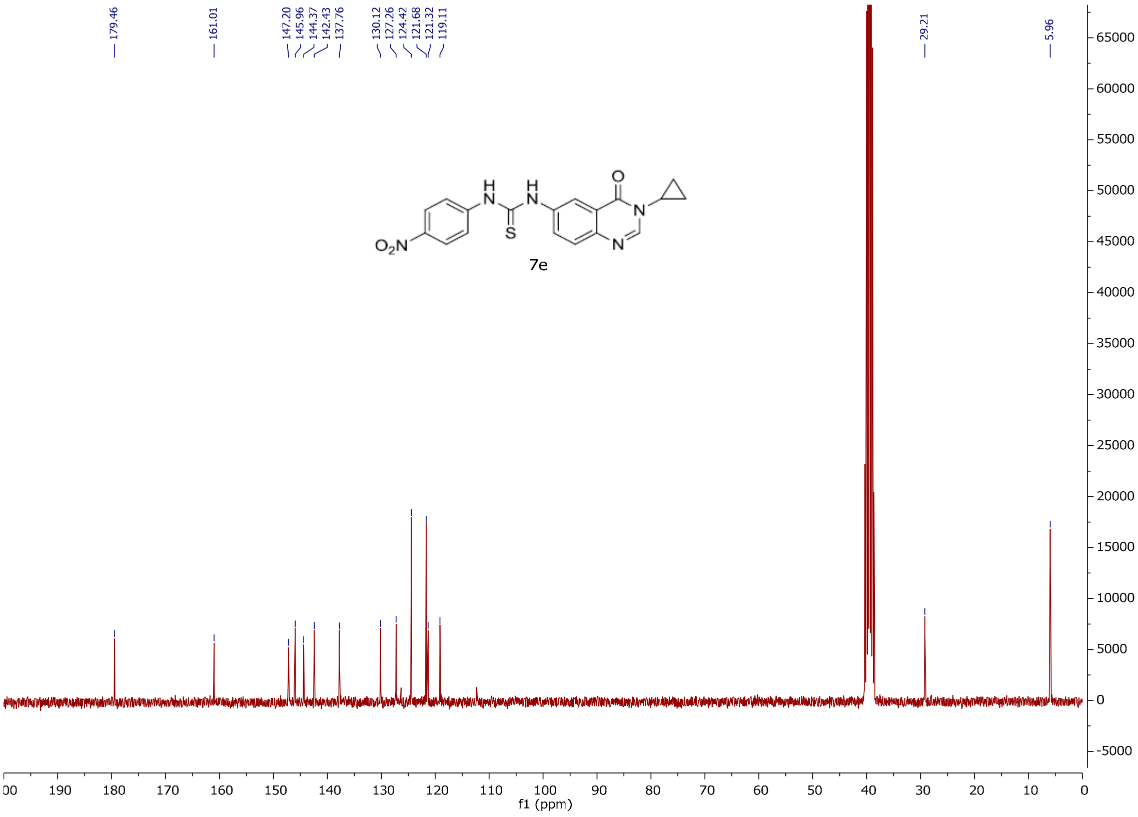

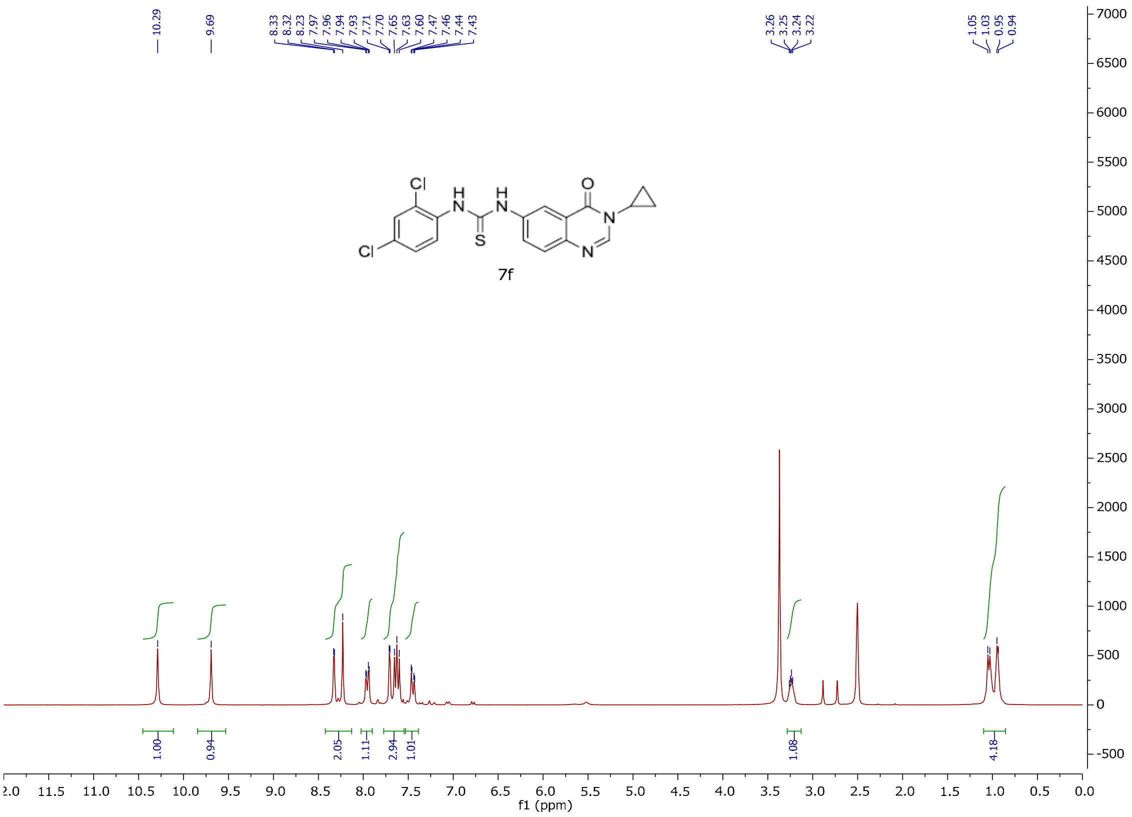

Figure S13. ^{1}H-NMR and ^{13}C-NMR Compound **7f**

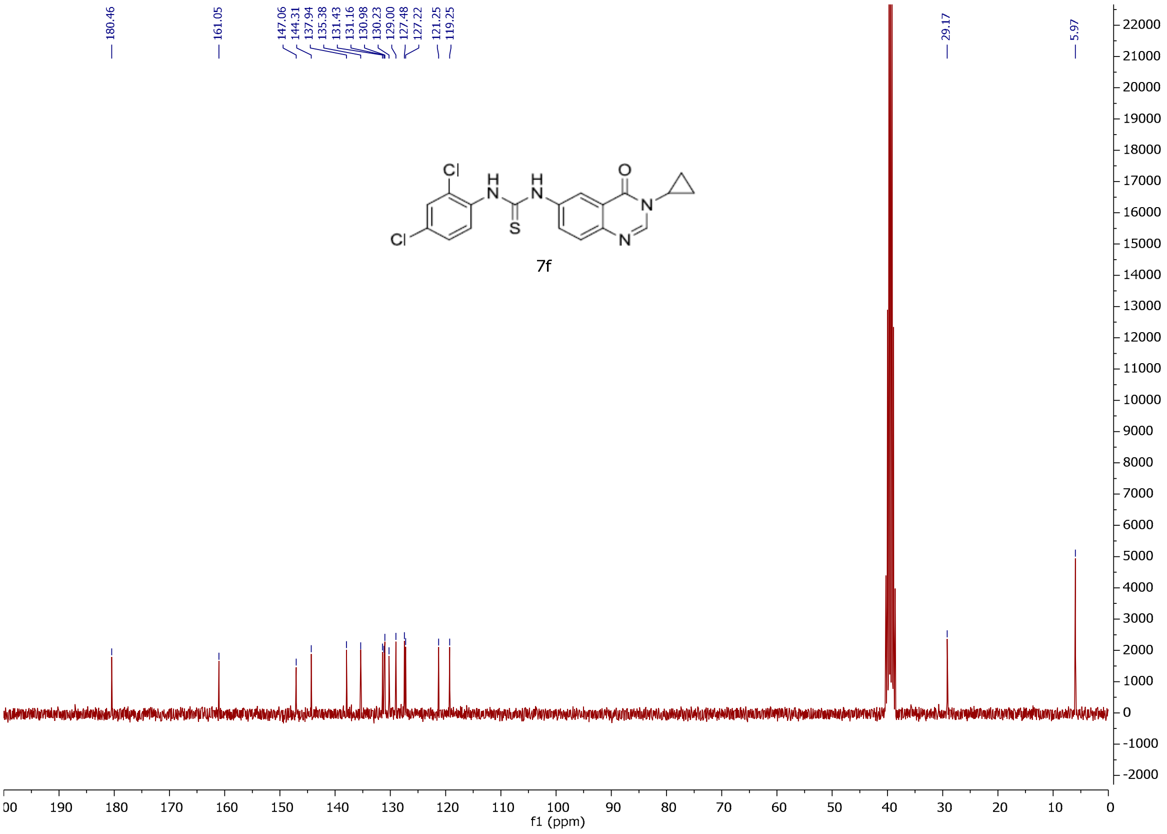

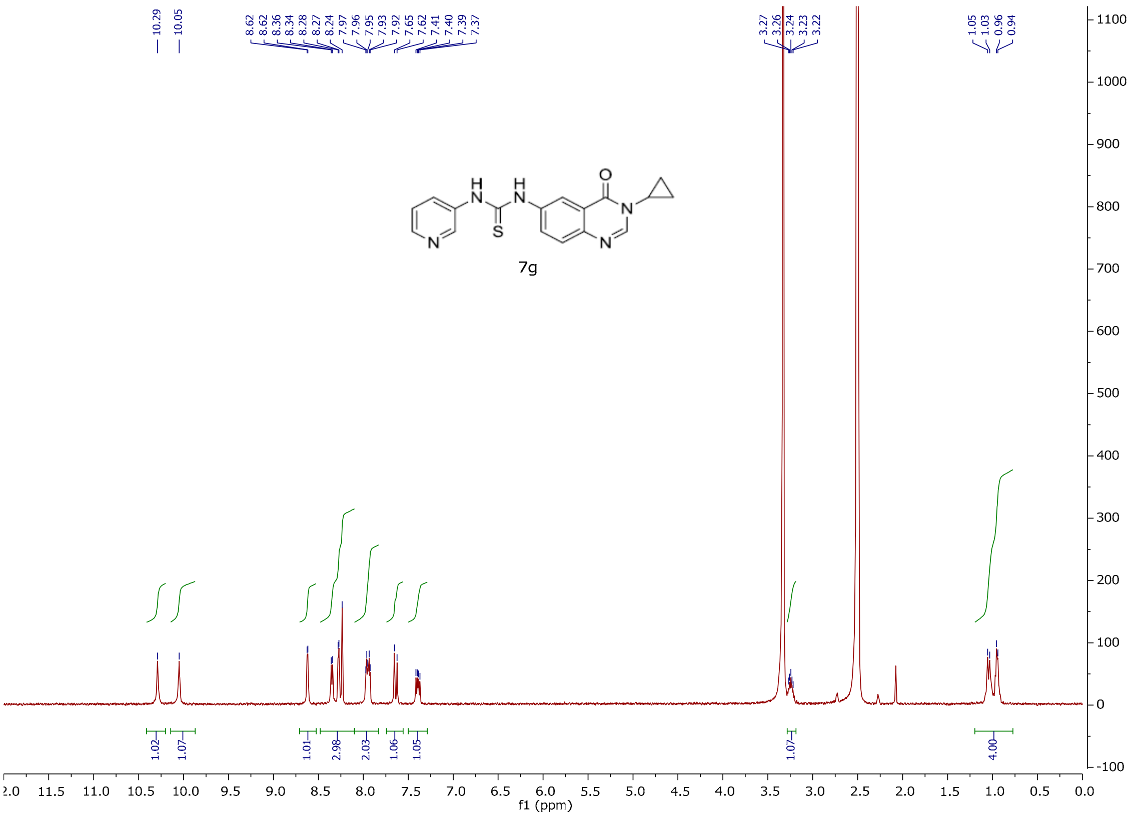

Figure S14. ^{1}H-NMR and ^{13}C-NMR Compound **7g**

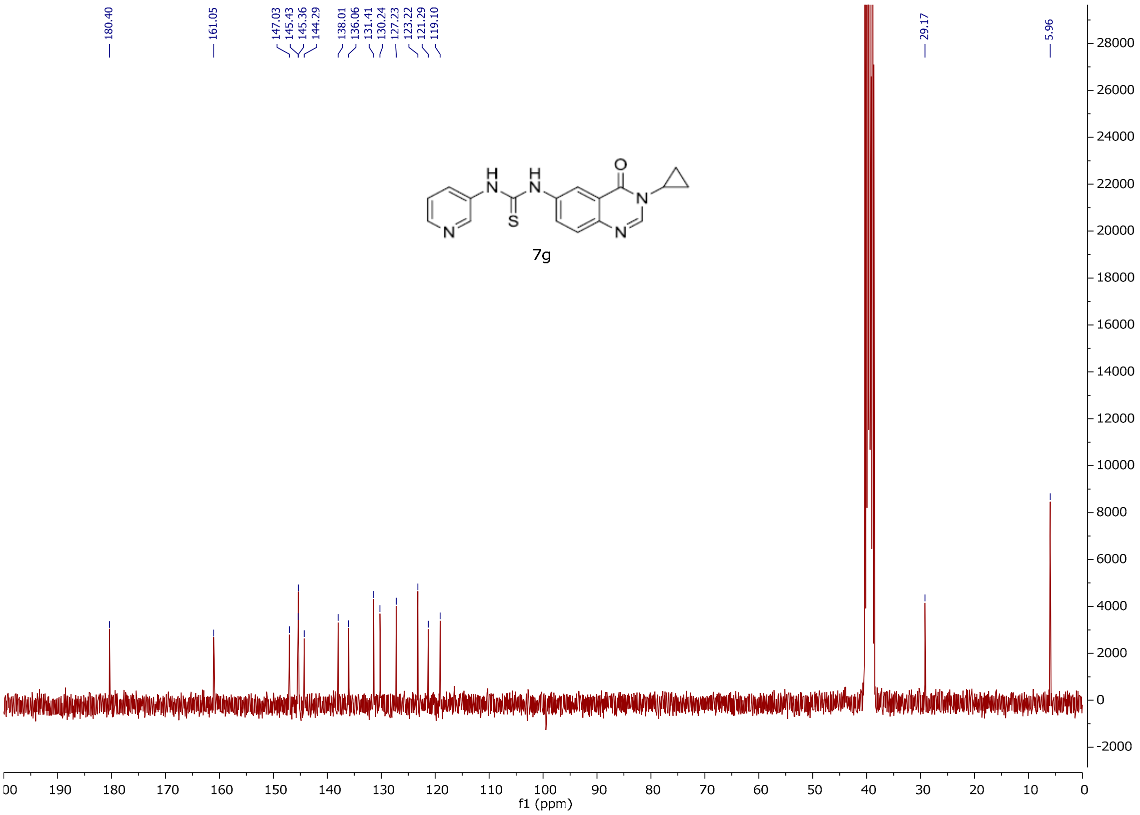

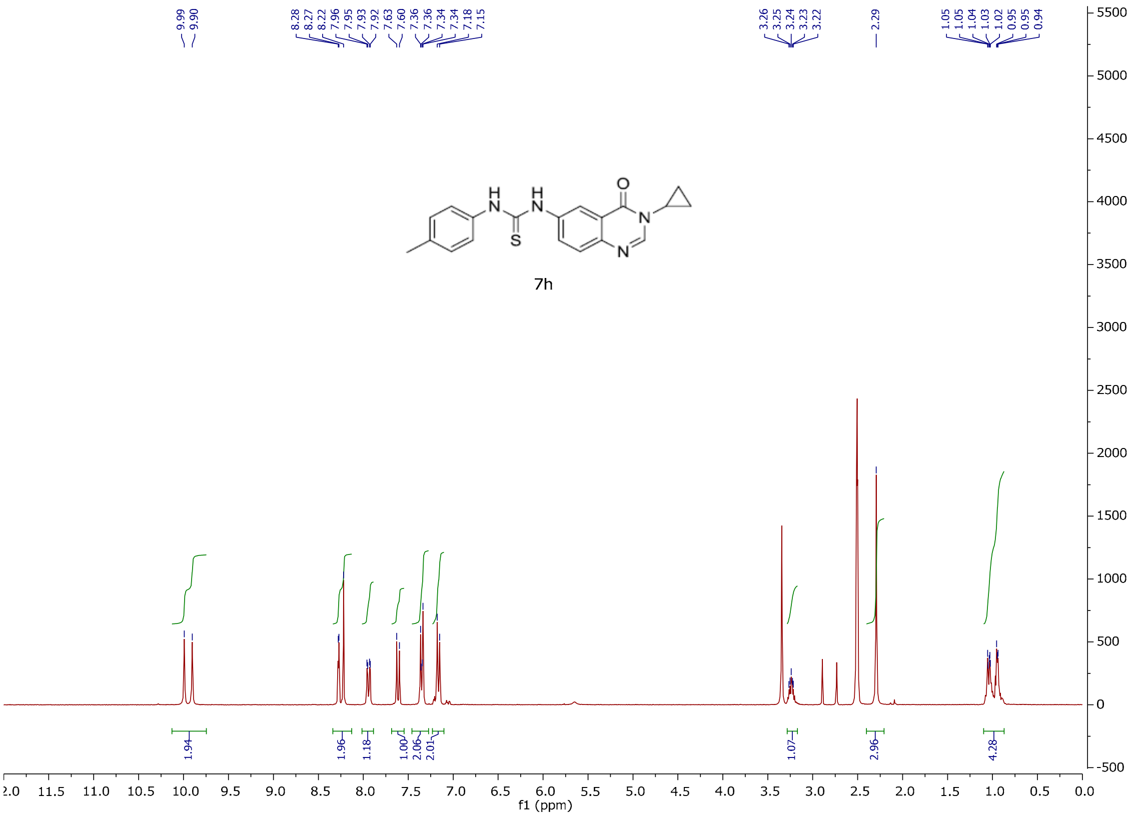

Figure S15. ^{1}H-NMR and ^{13}C-NMR Compound **7h**

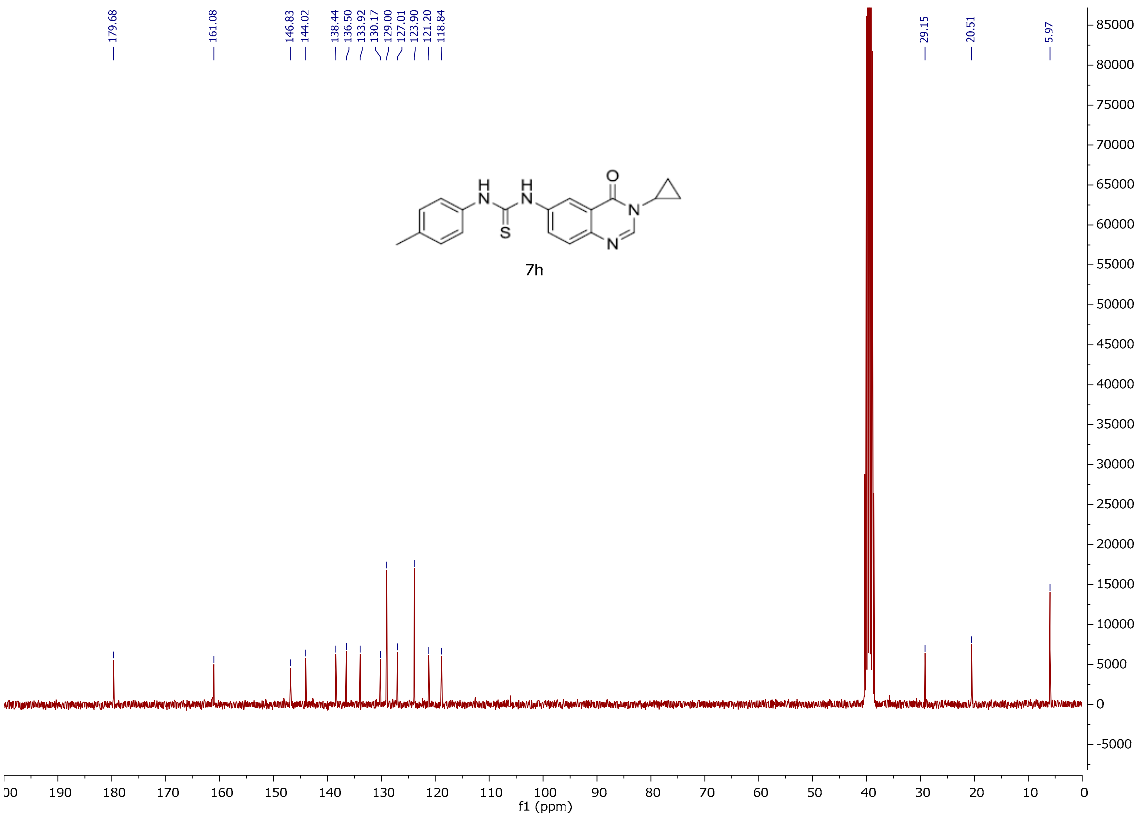

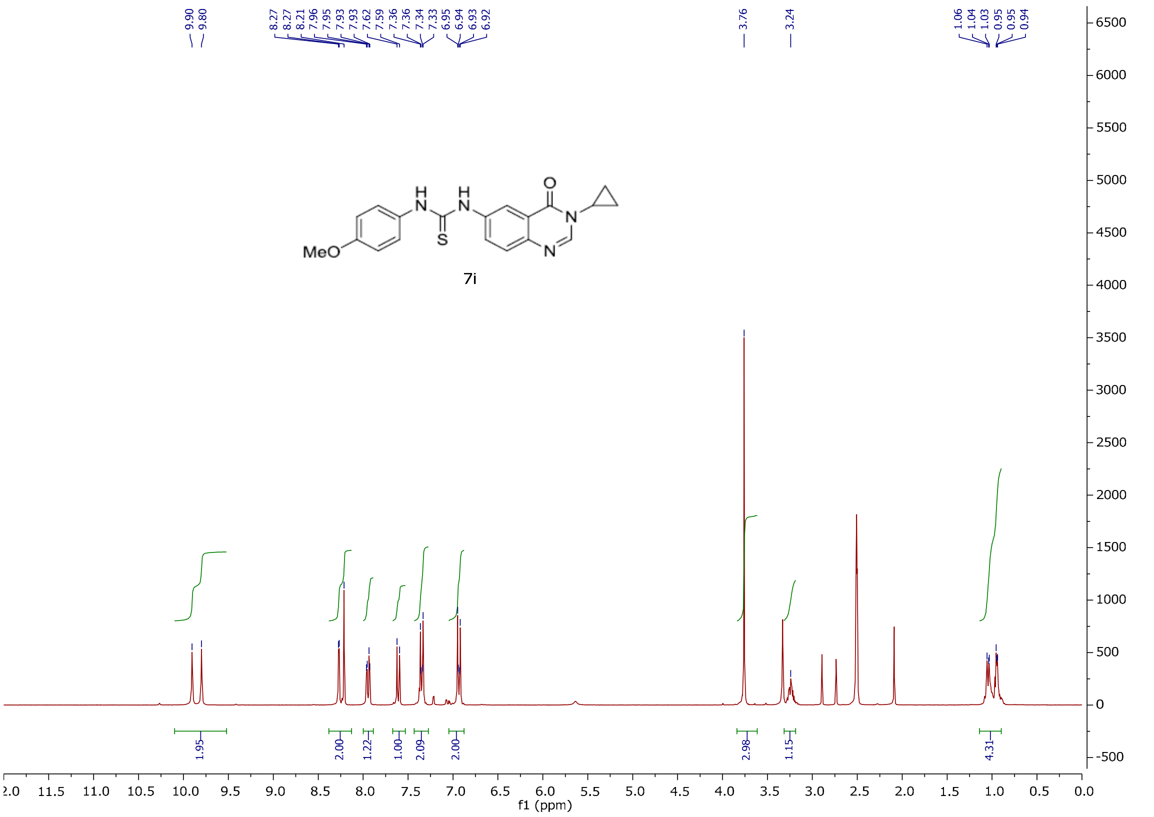

Figure S16. ^{1}H-NMR and ^{13}C-NMR Compound **7i**

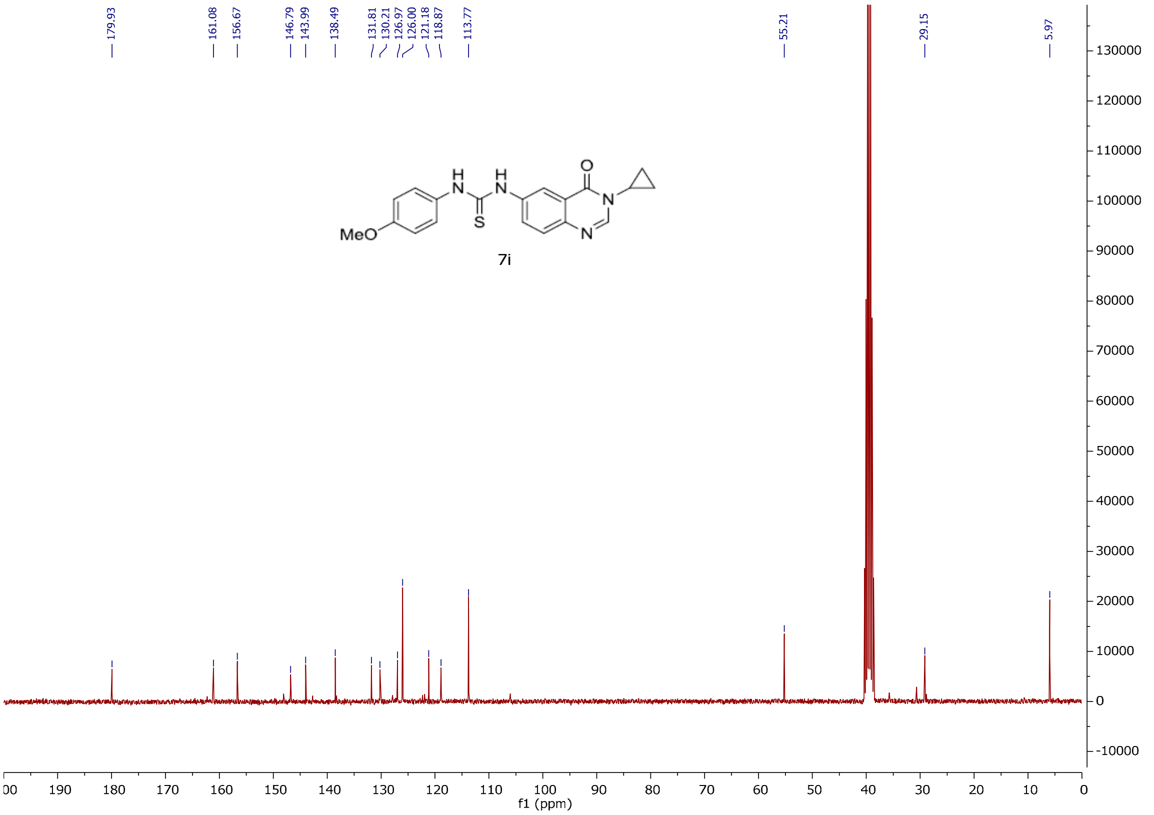

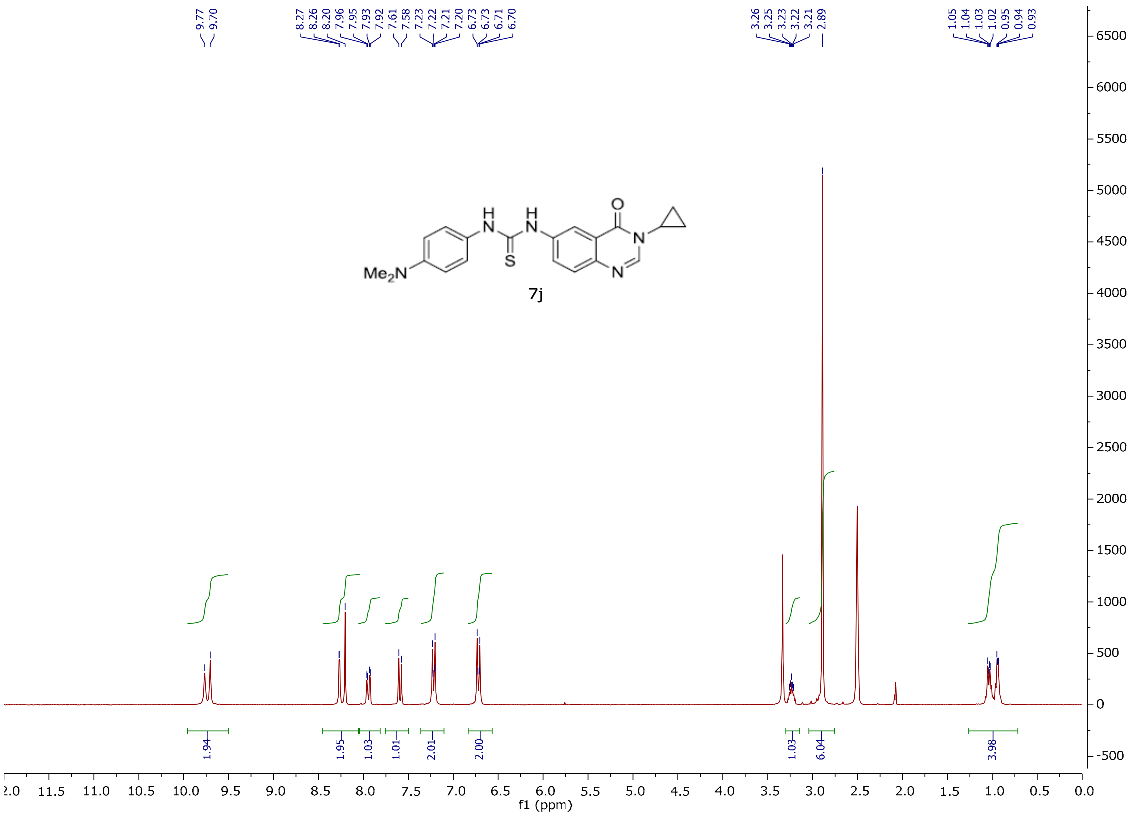

Figure S17. ^{1}H-NMR and ^{13}C-NMR Compound **7j**

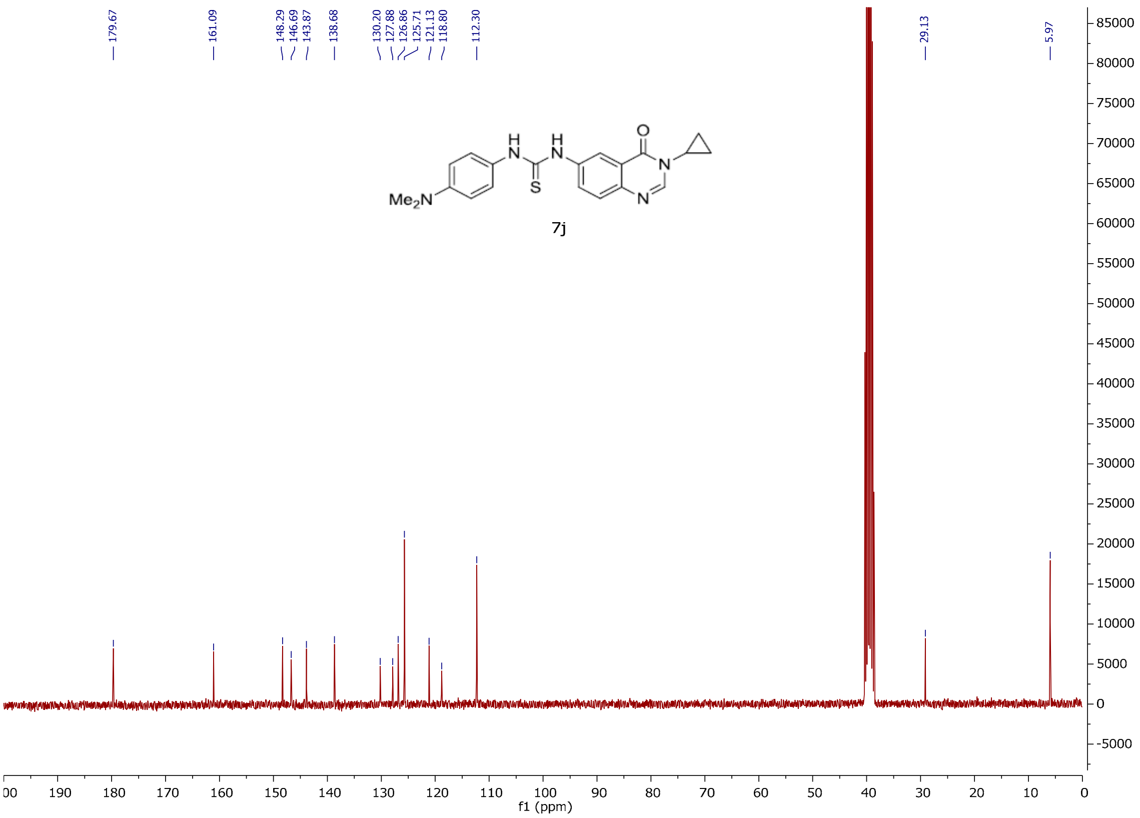

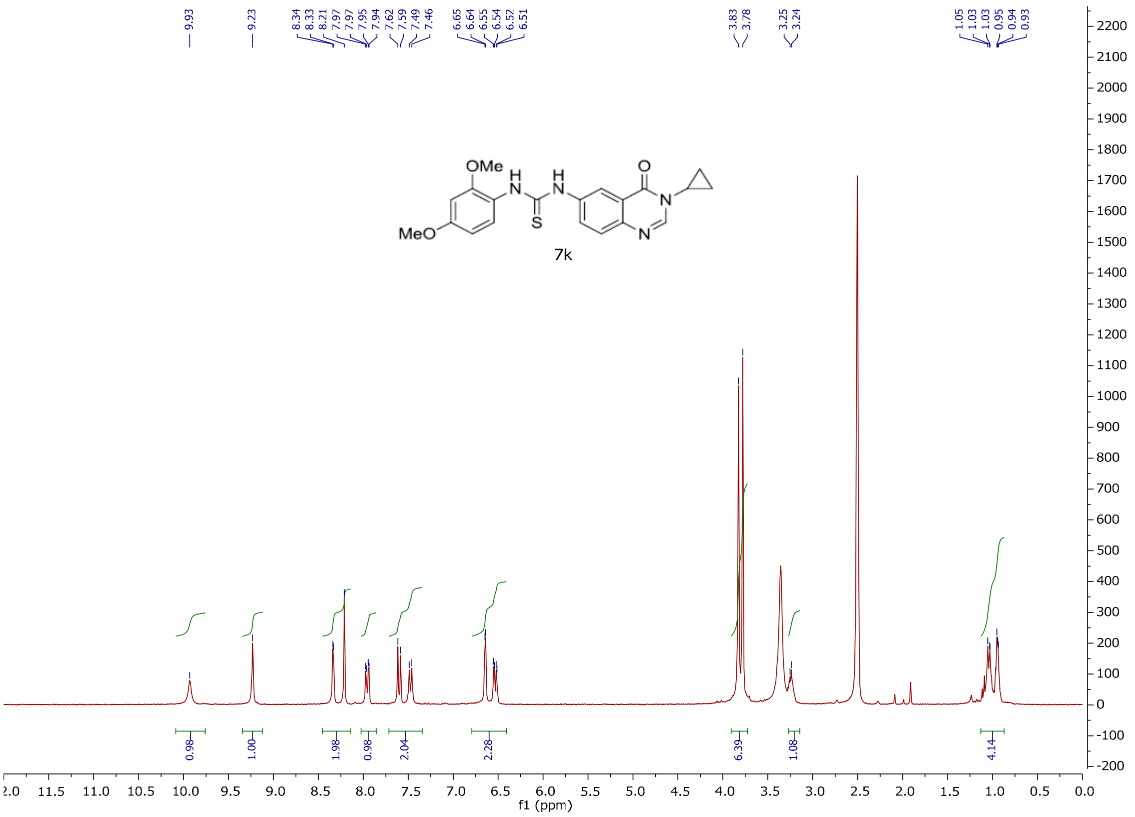

Figure S18. ^{1}H-NMR and ^{13}C-NMR Compound **7k**

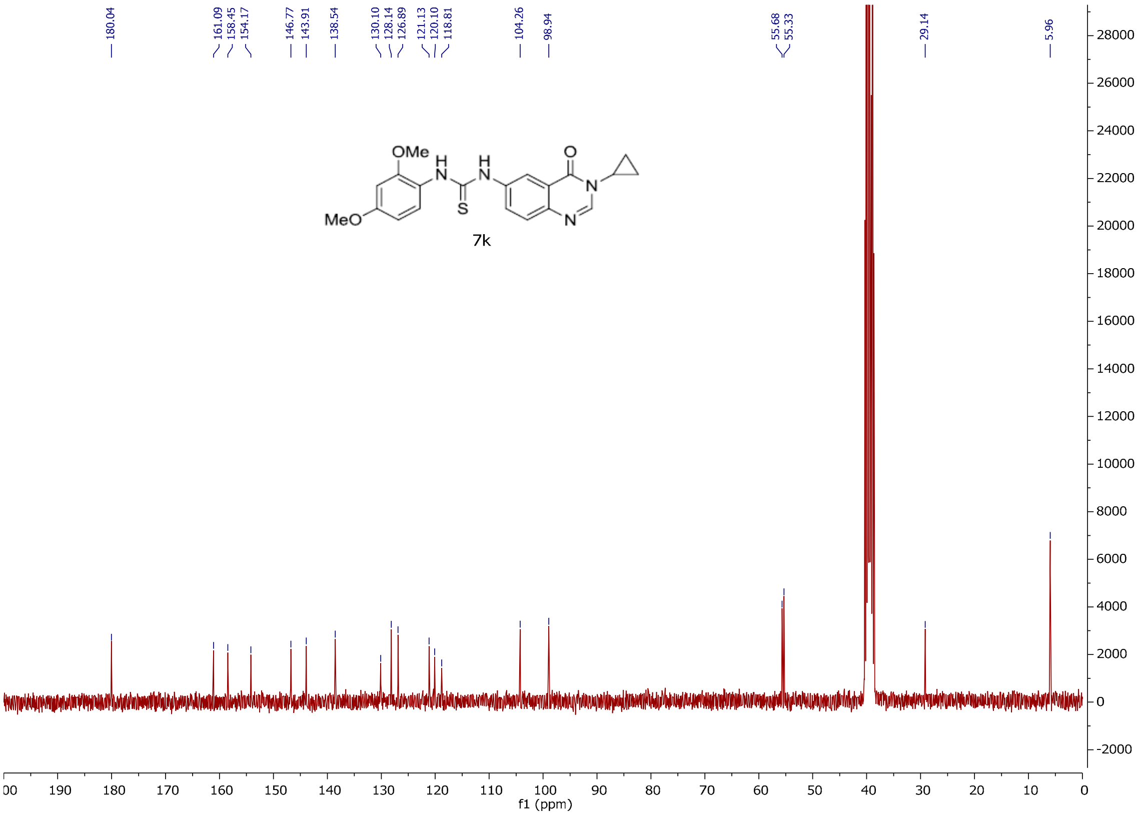

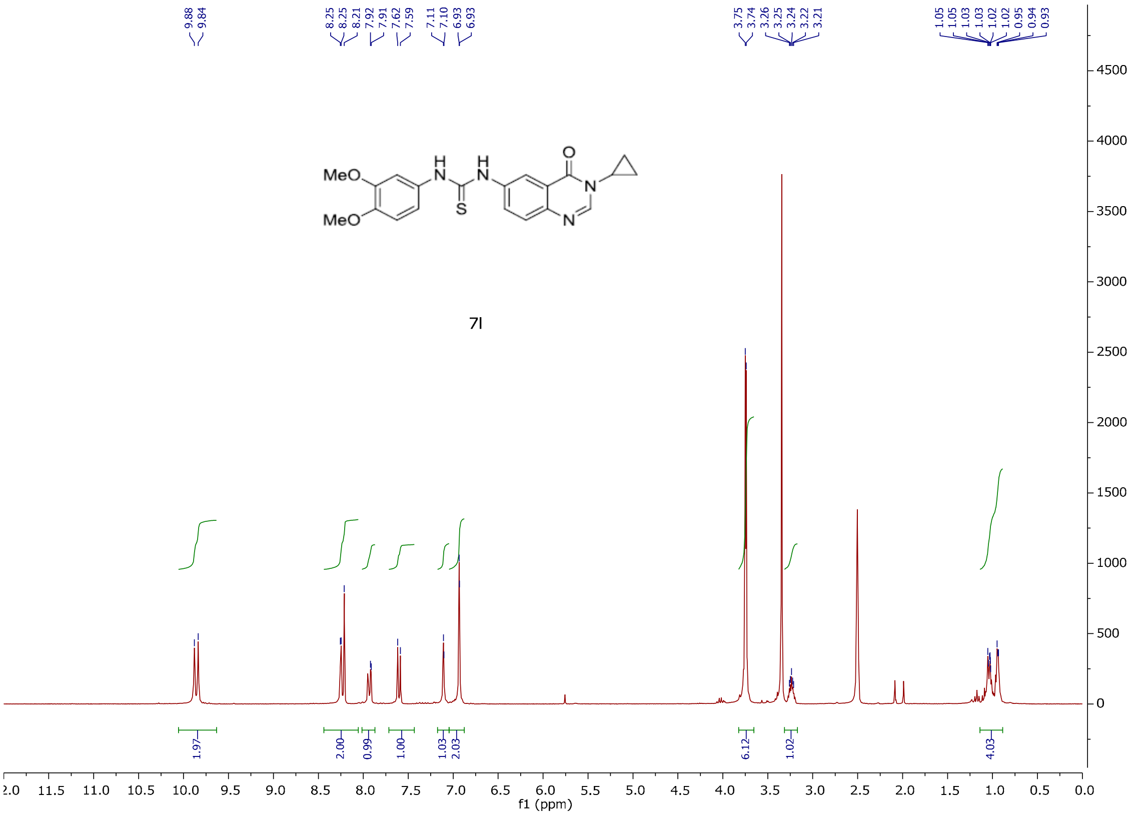

Figure S19. ^{1}H-NMR and ^{13}C-NMR Compound **7l**

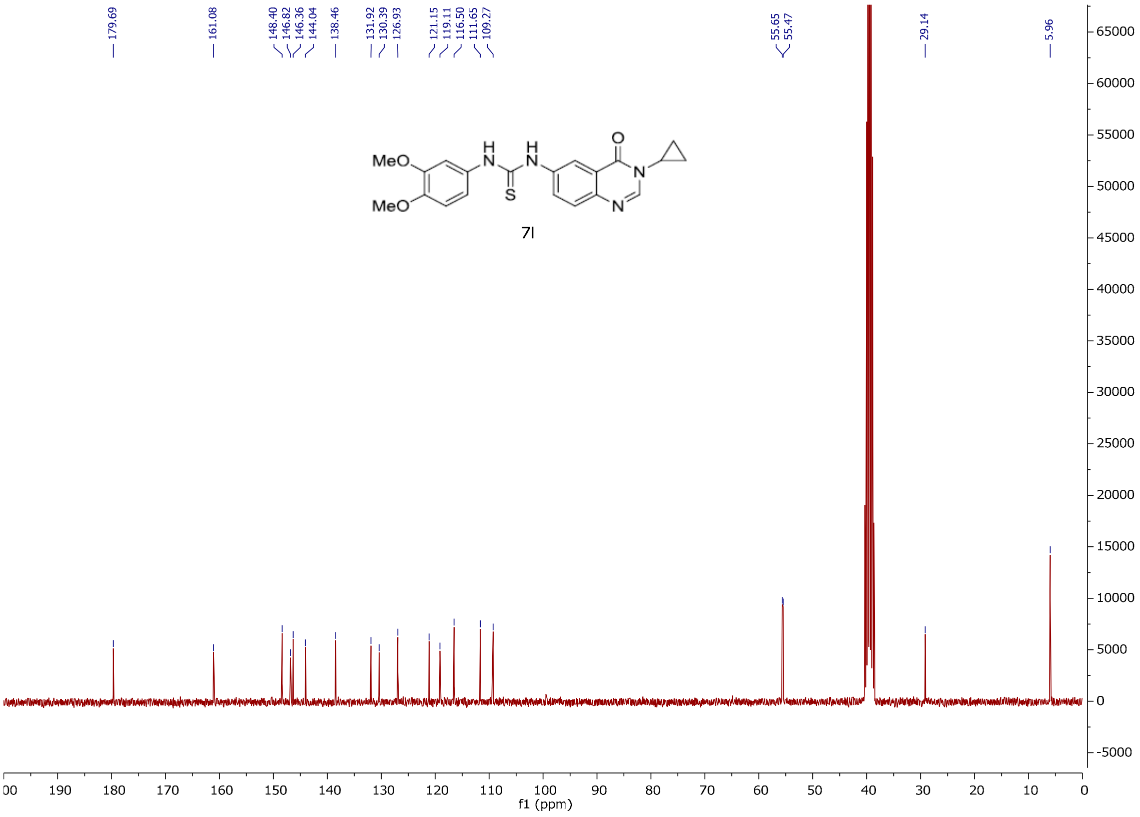

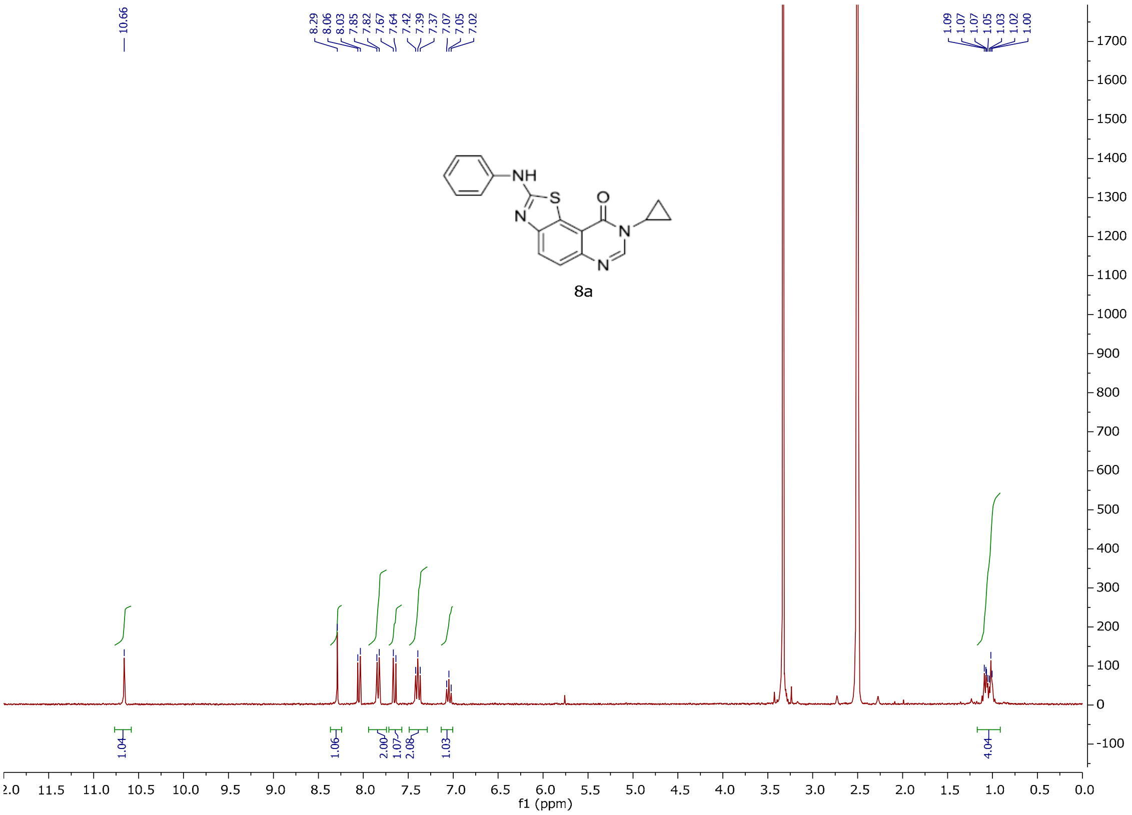

Figure S20. ^{1}H-NMR Compound **8a**

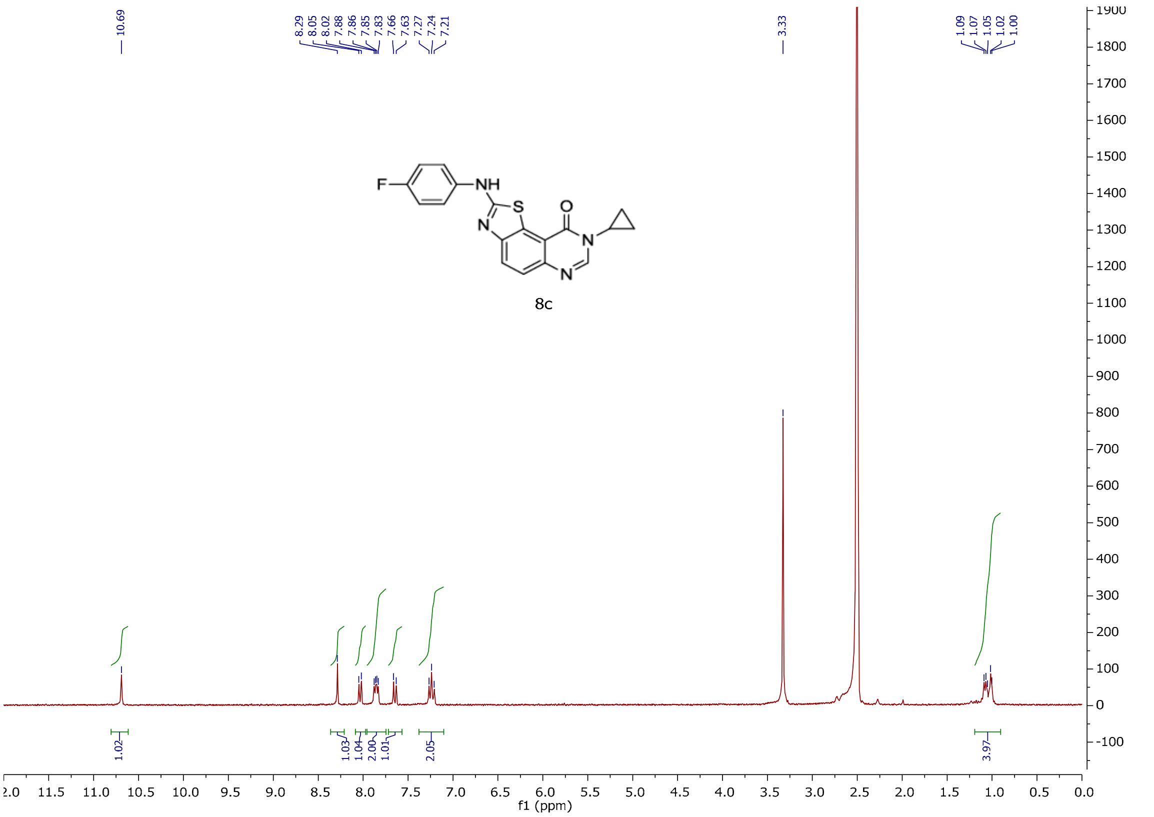

Figure S21. ^{1}H-NMR and ^{13}C-NMR Compound **8c**

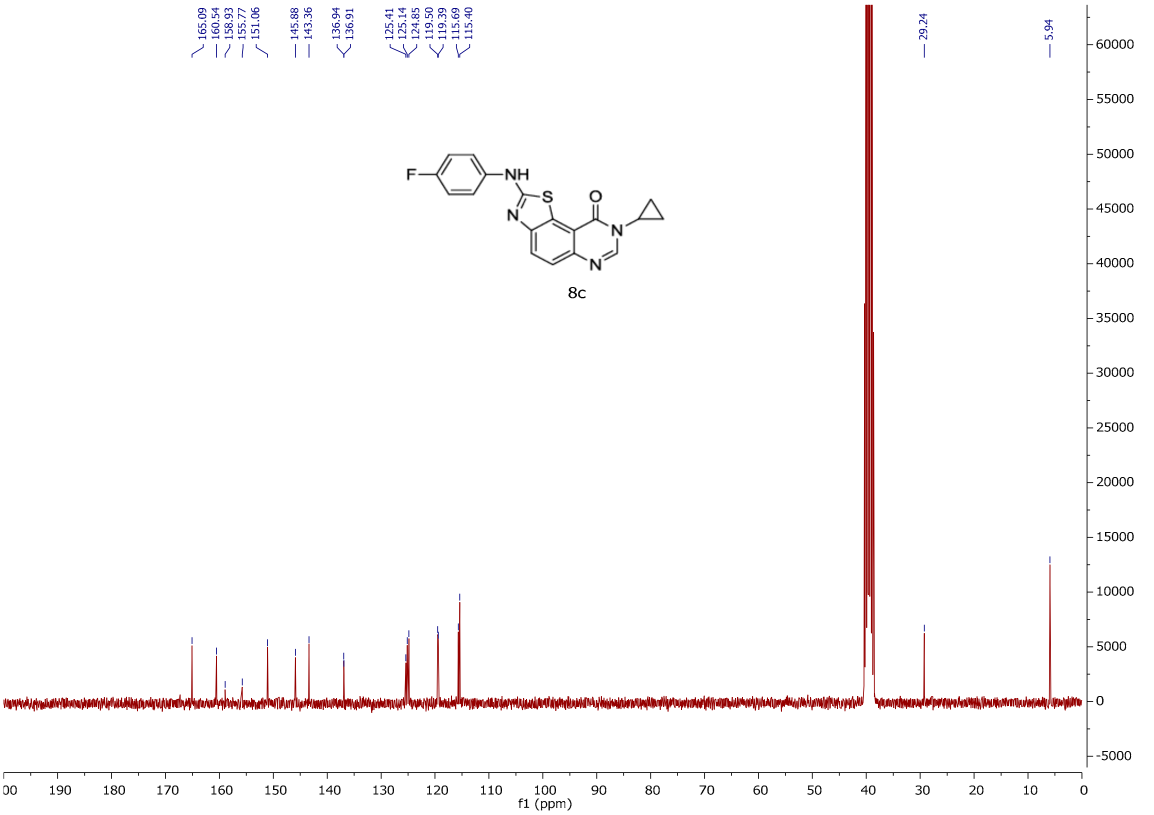

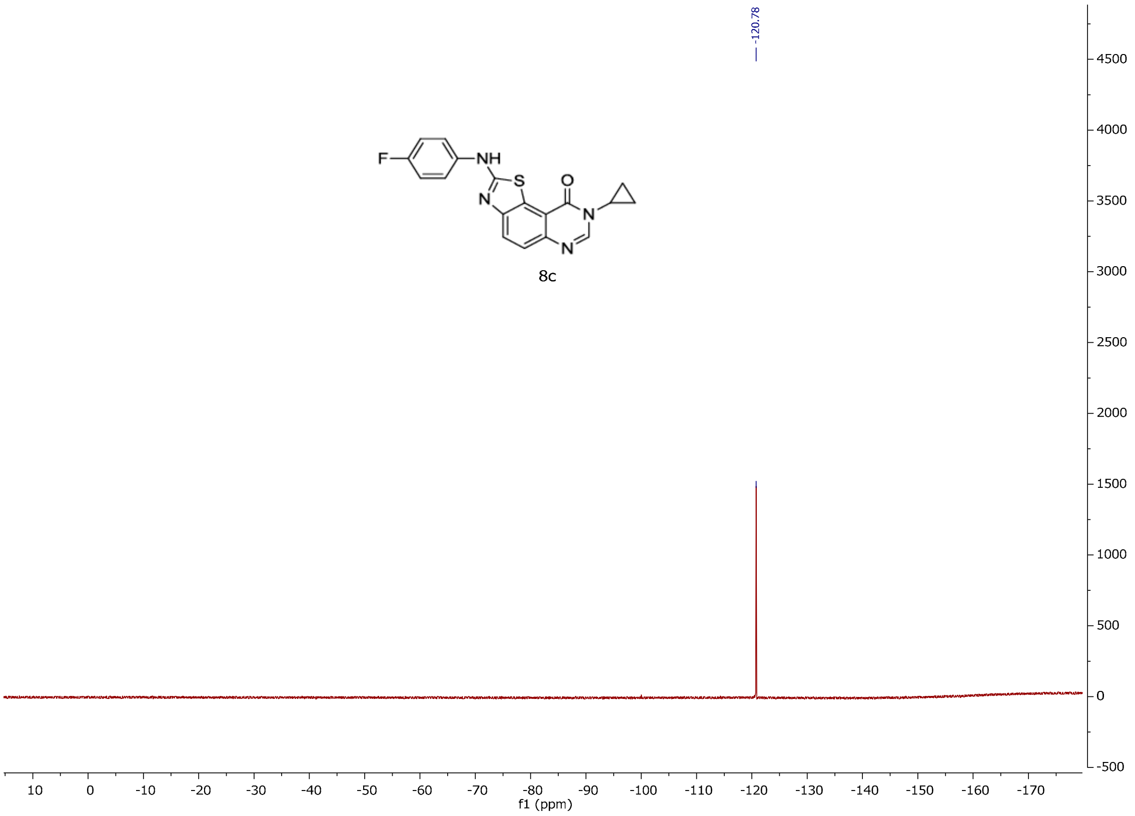

Figure S22. ^{19}F-NMR Compound **8c**

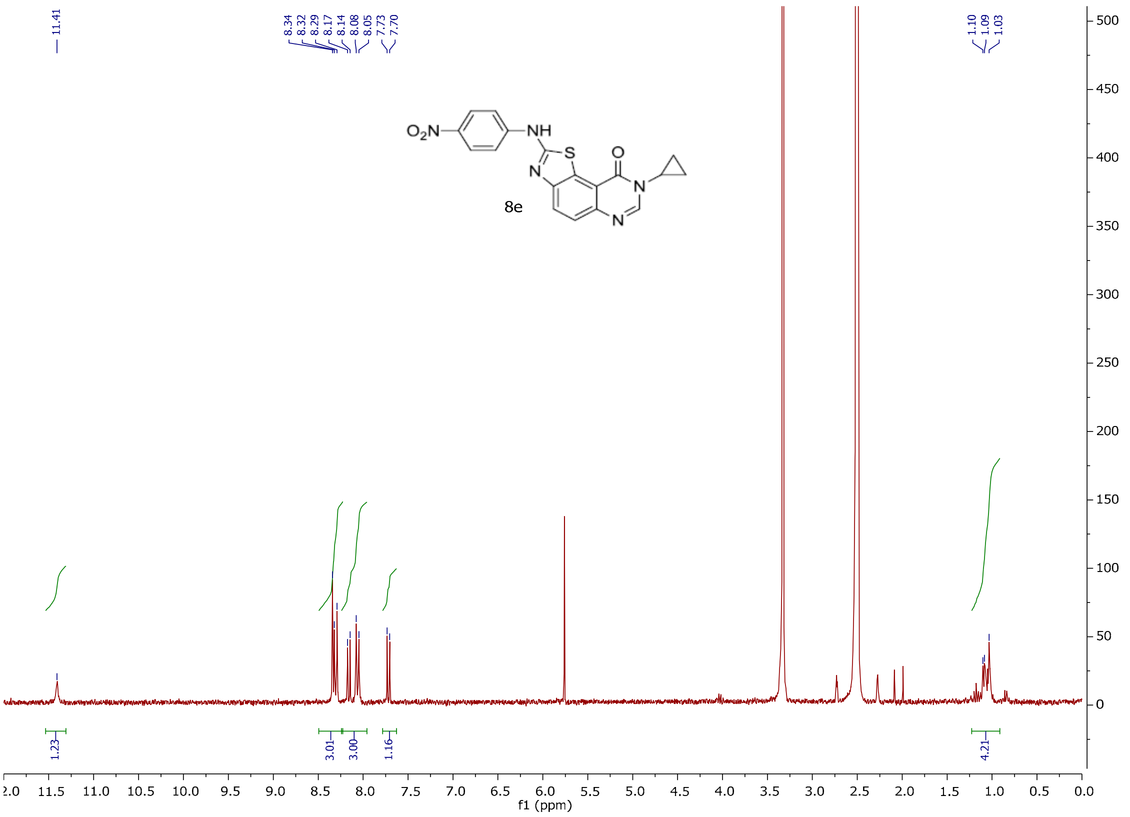

Figure S23. ^{1}H-NMR Compound **82**

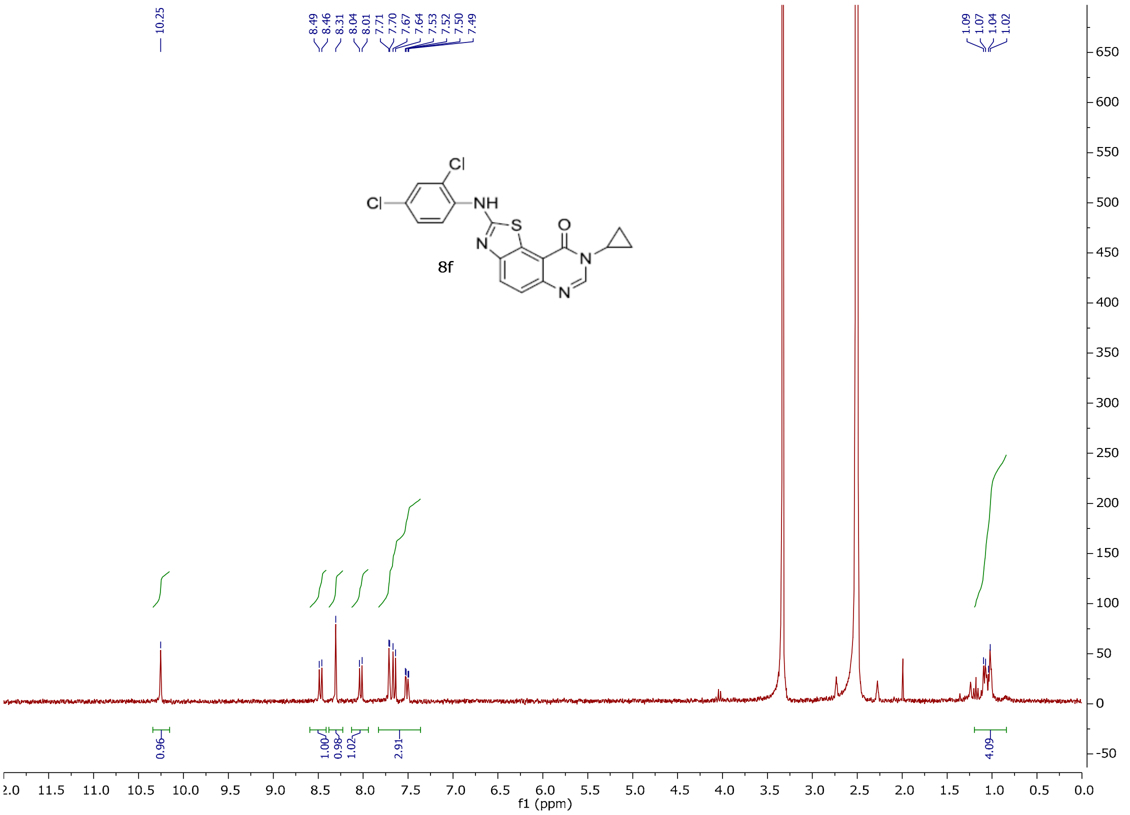

Figure S24. ^{1}H-NMR and ^{13}C-NMR Compound **8f**

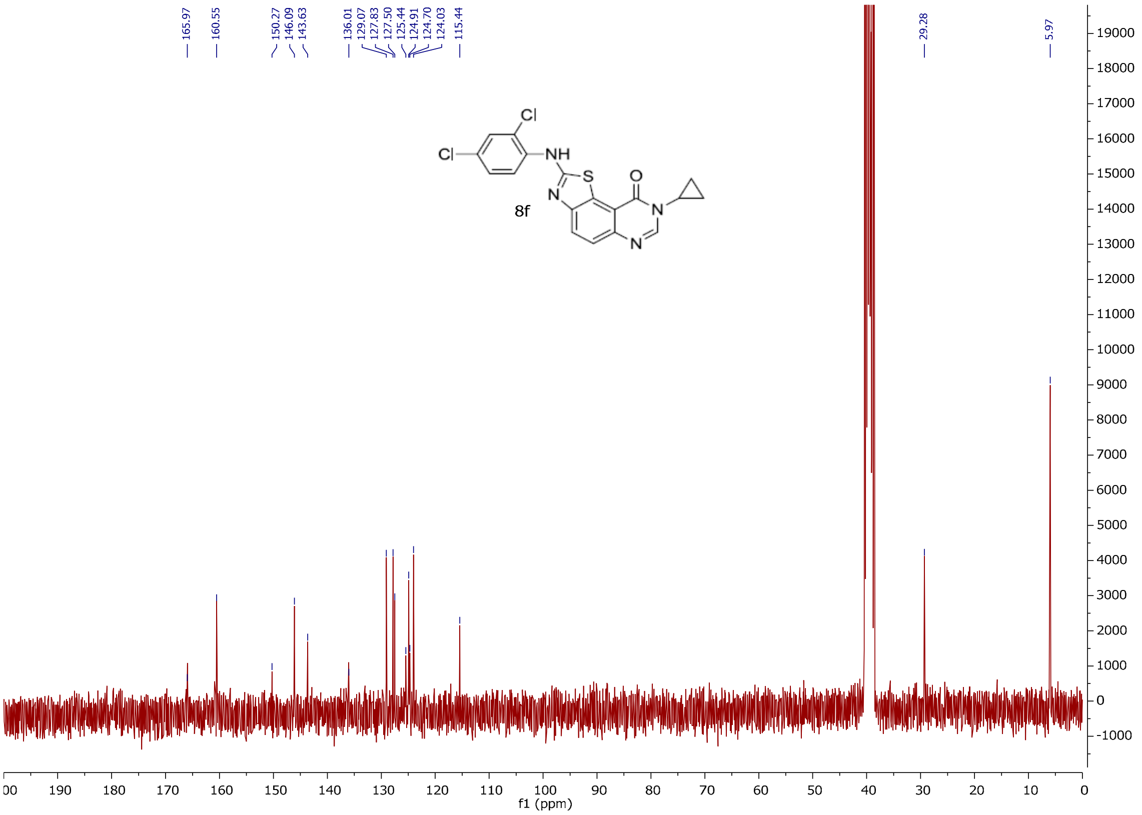

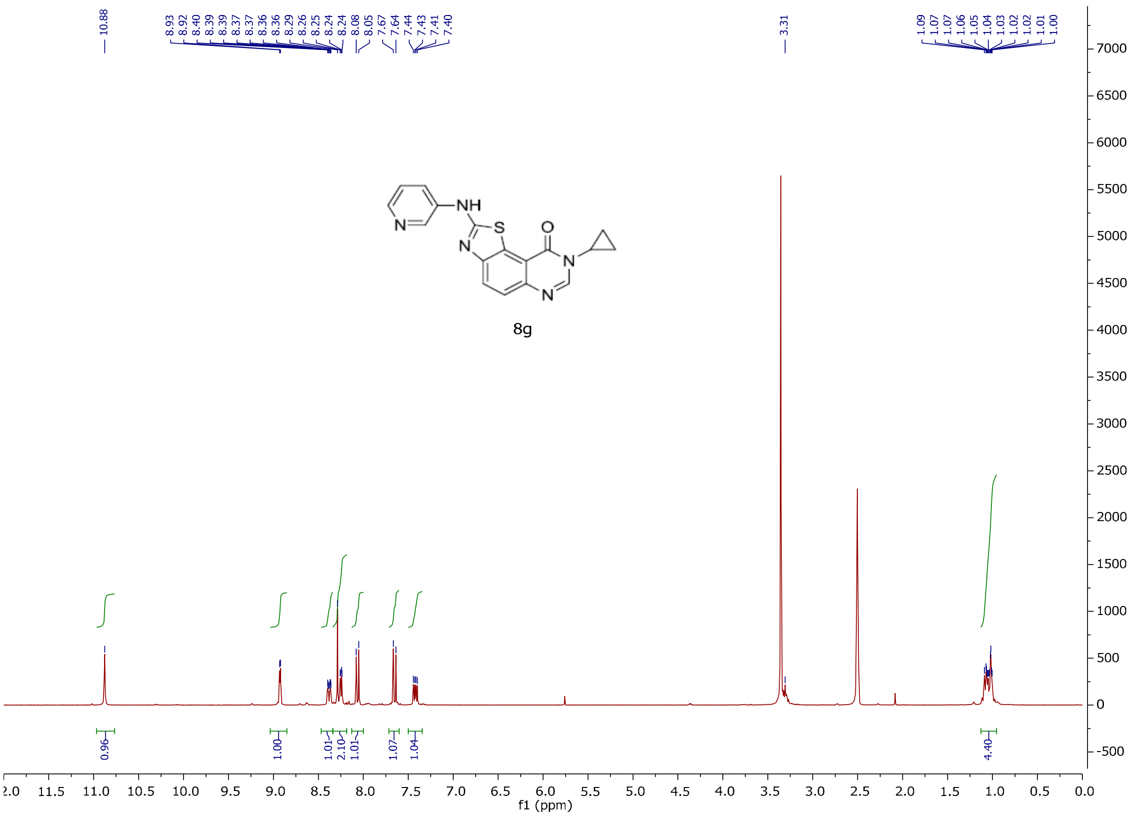

Figure S25. ¹H-NMR and ¹³C-NMR Compound **8g**

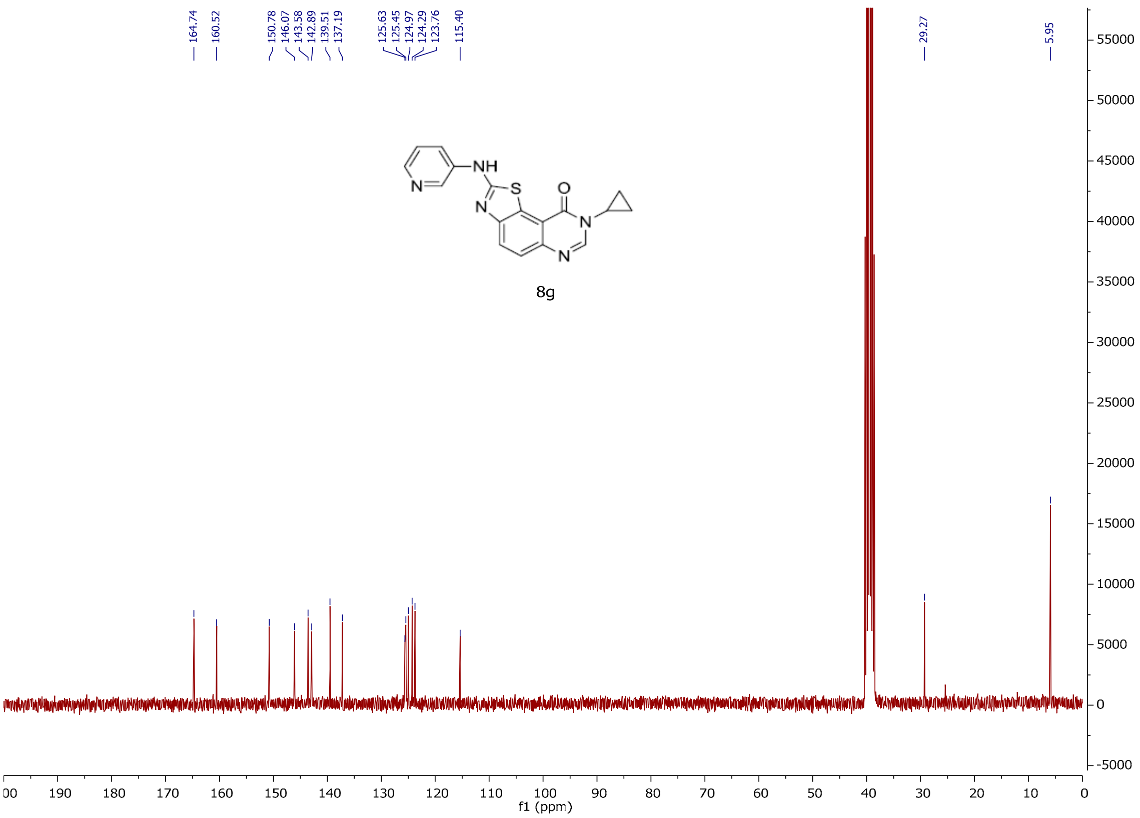

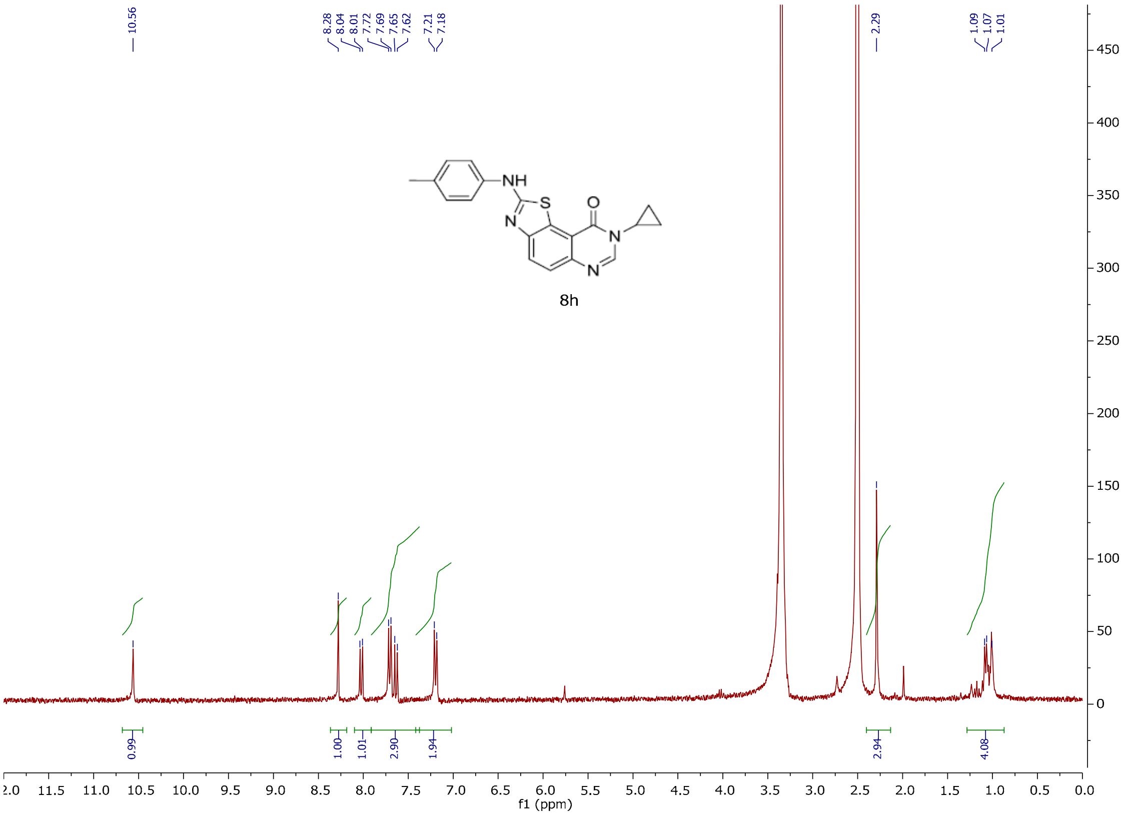

Figure S26. ^{1}H-NMR and ^{13}C-NMR Compound **8h**

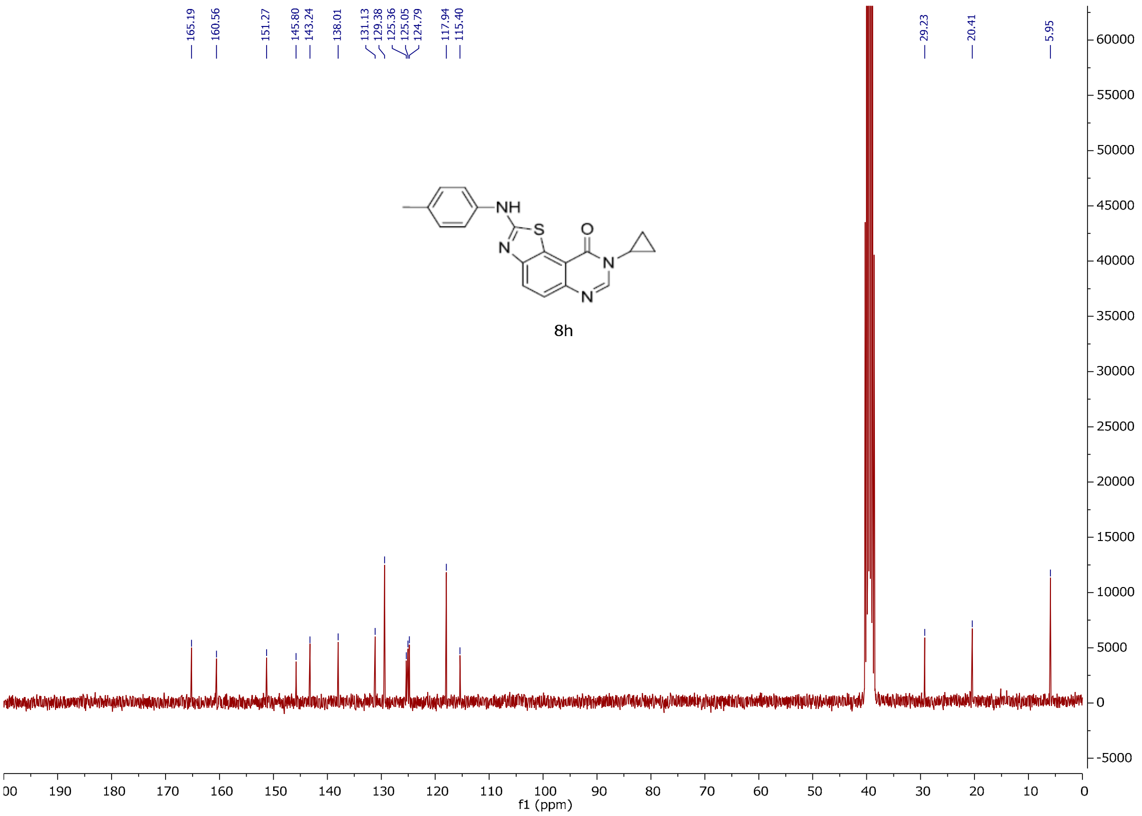

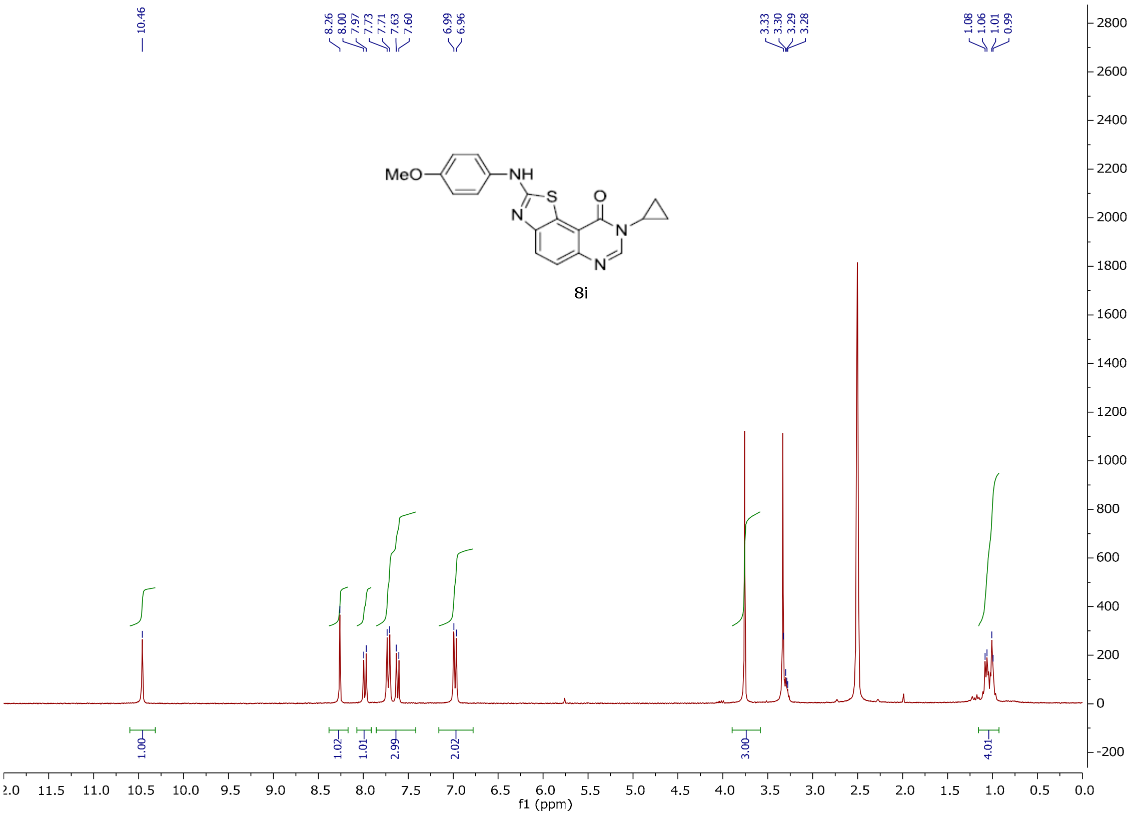

Figure S27. ^{1}H-NMR and ^{13}C-NMR Compound **8i**

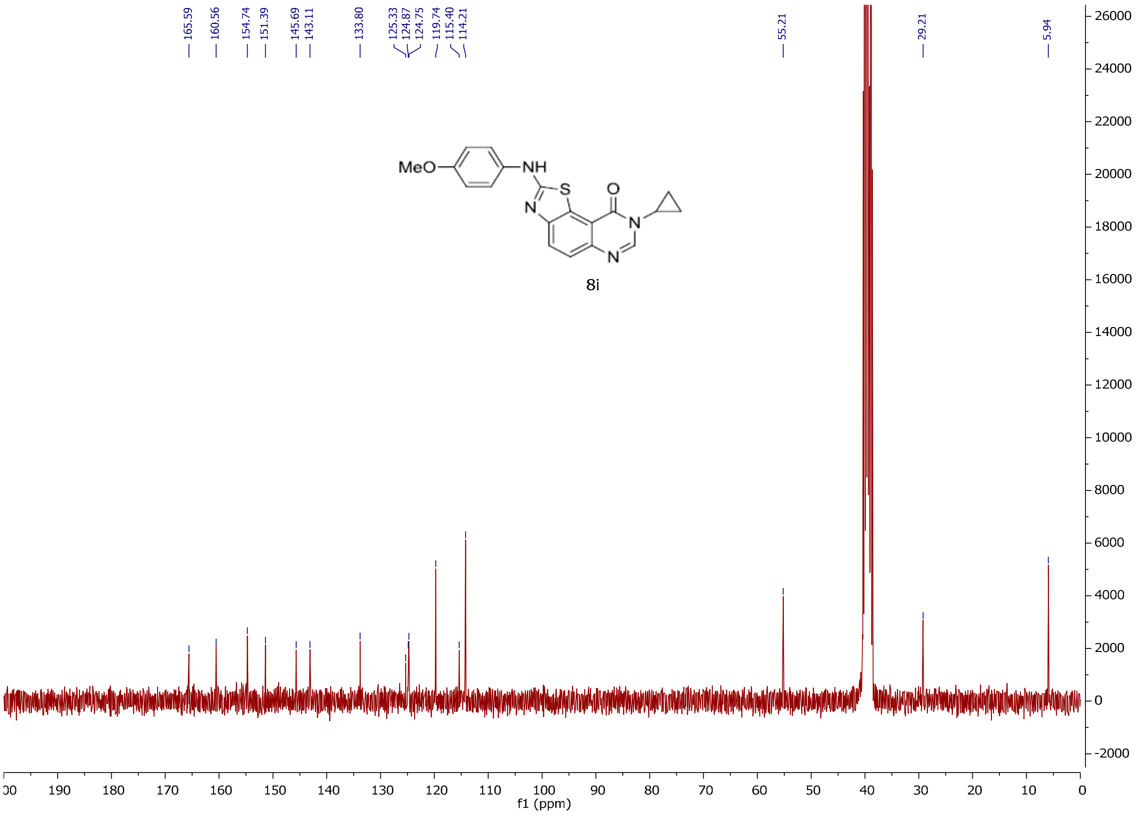

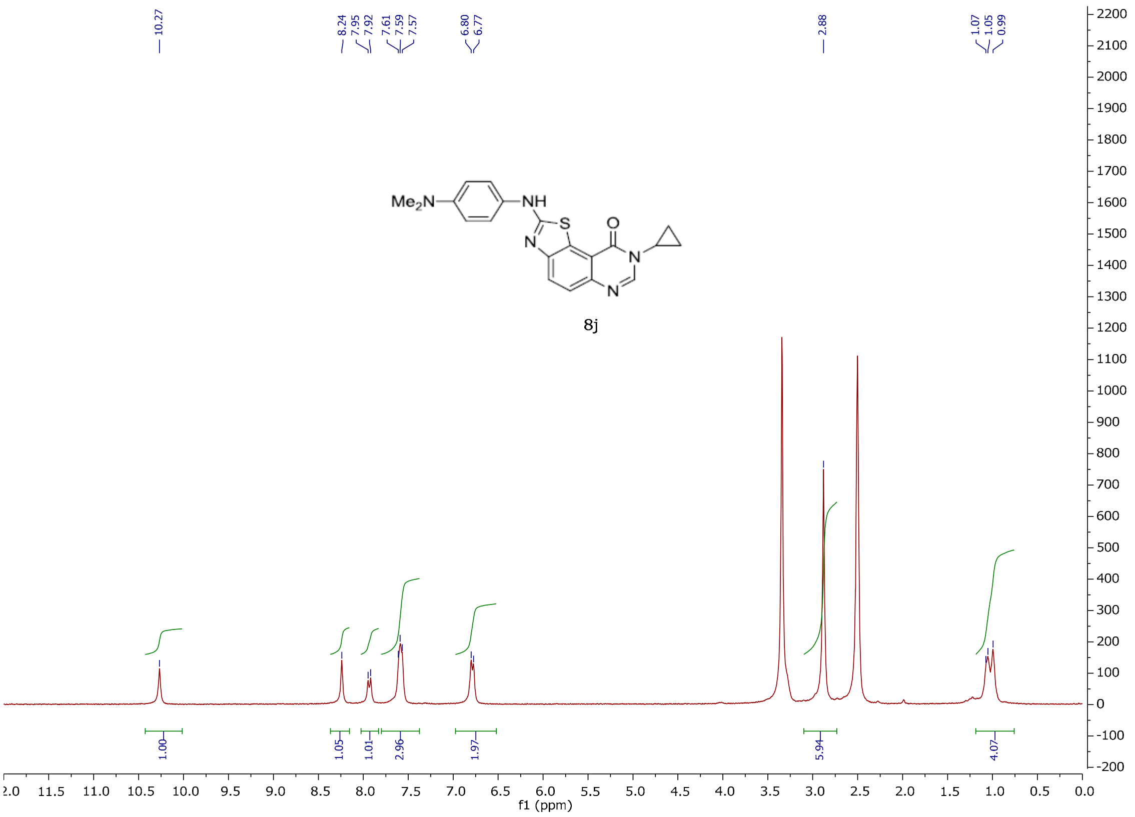

Figure S28. ^{1}H-NMR and ^{13}C-NMR Compound **8j**

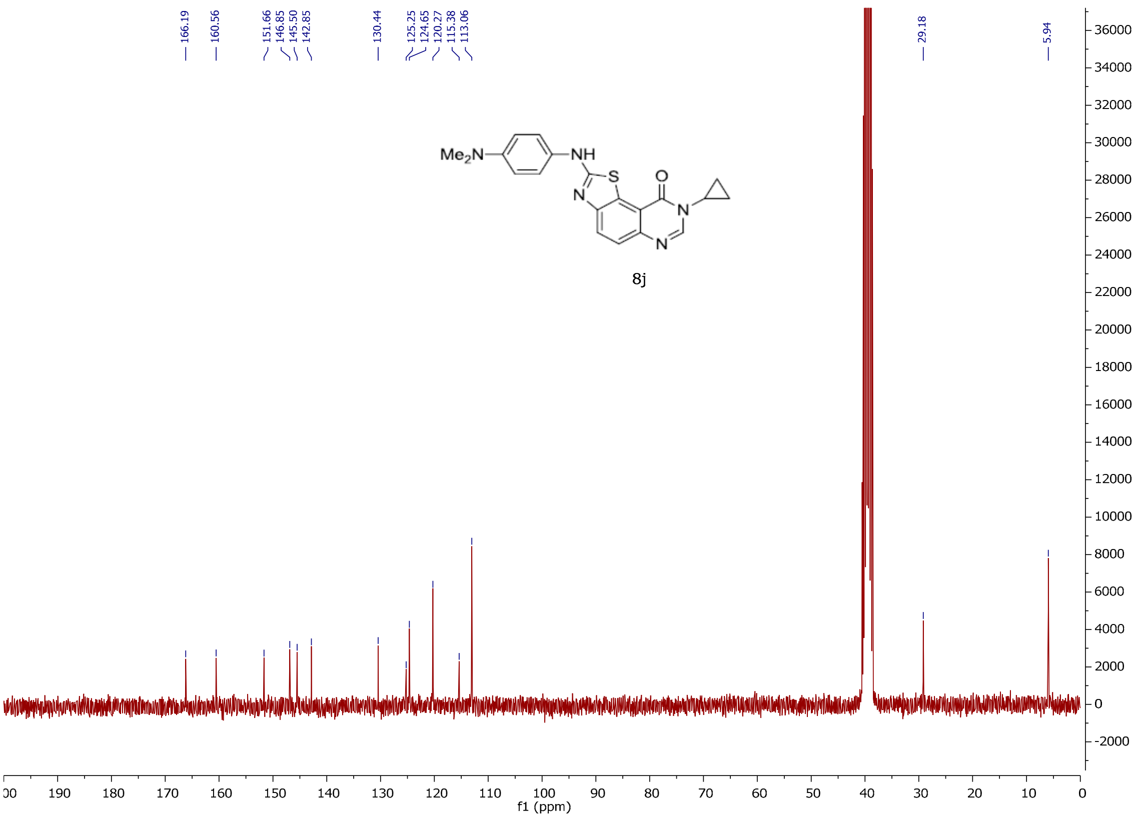

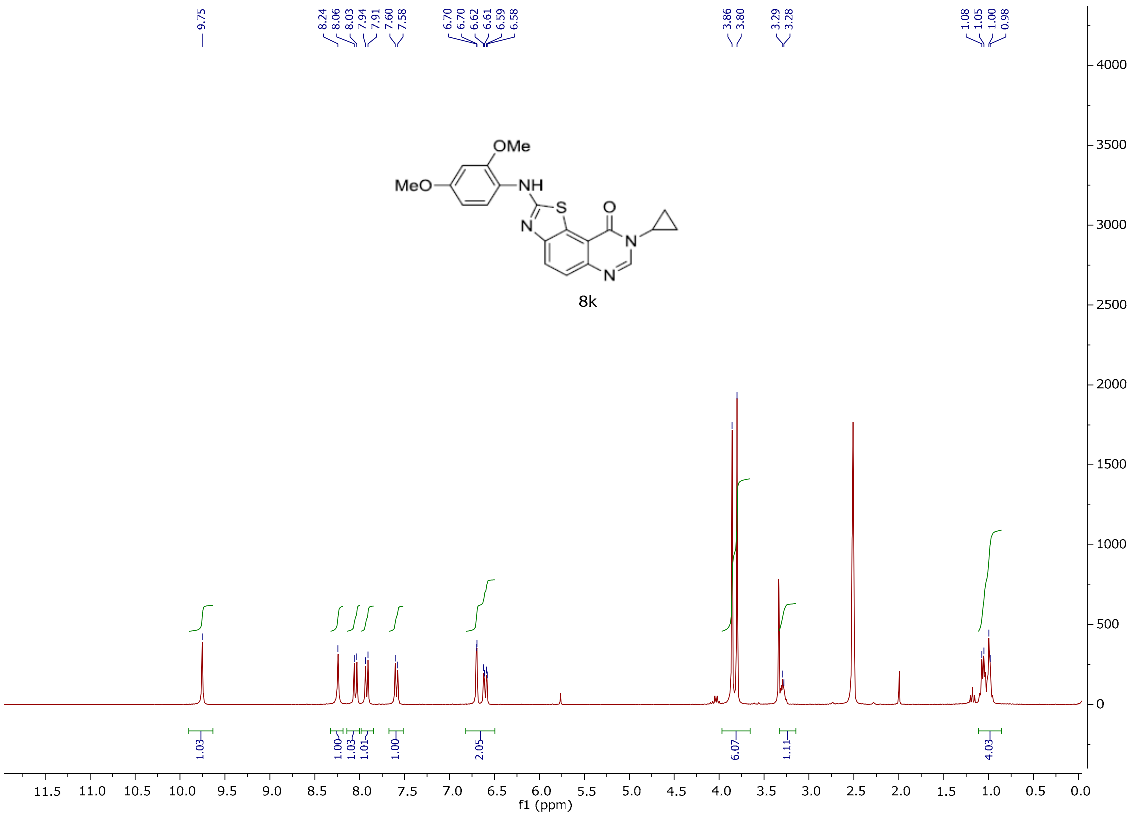

Figure S29. ^{1}H-NMR and ^{13}C-NMR Compound **8k**

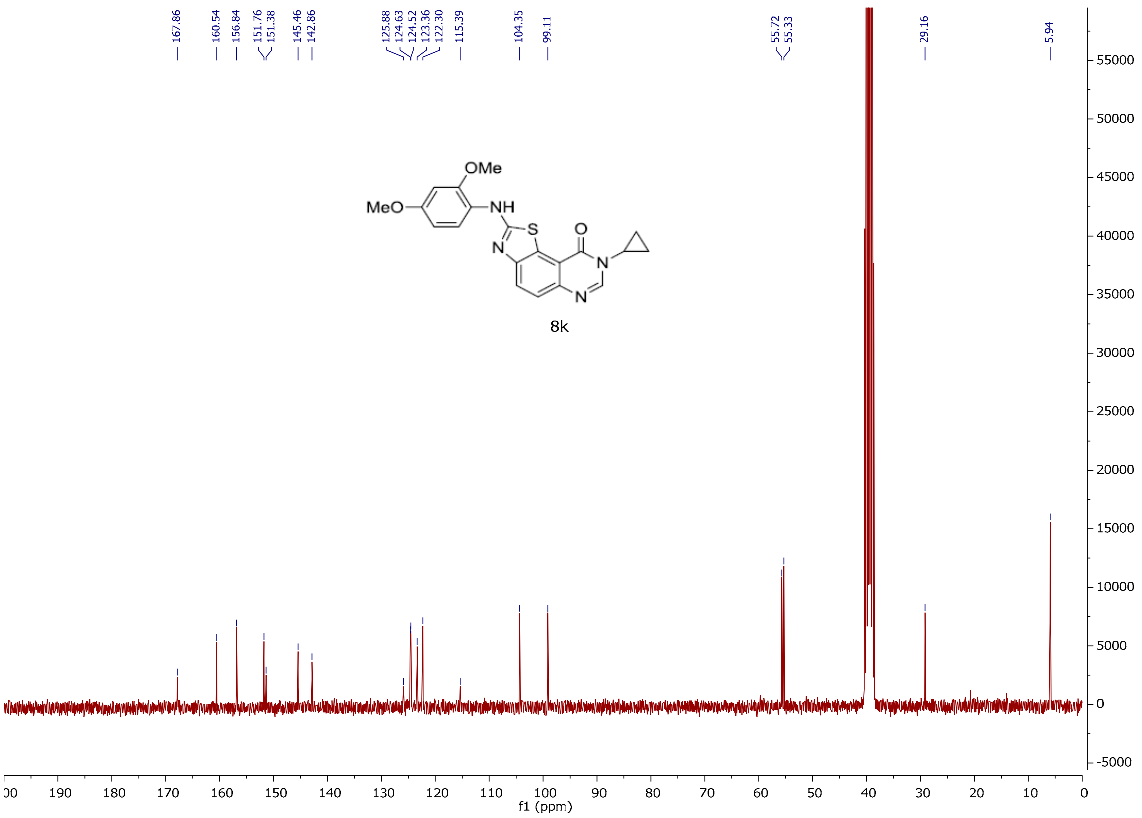

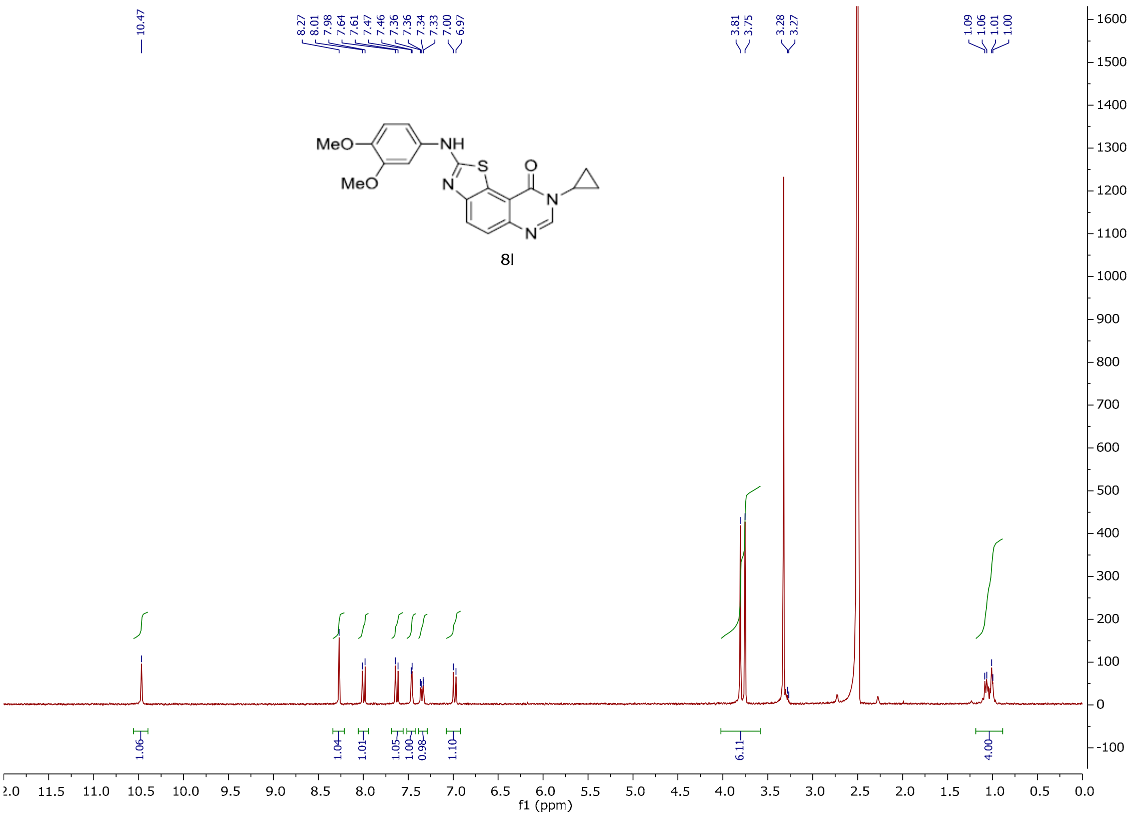

Figure S30. ^{1}H-NMR and ^{13}C-NMR Compound **8l**

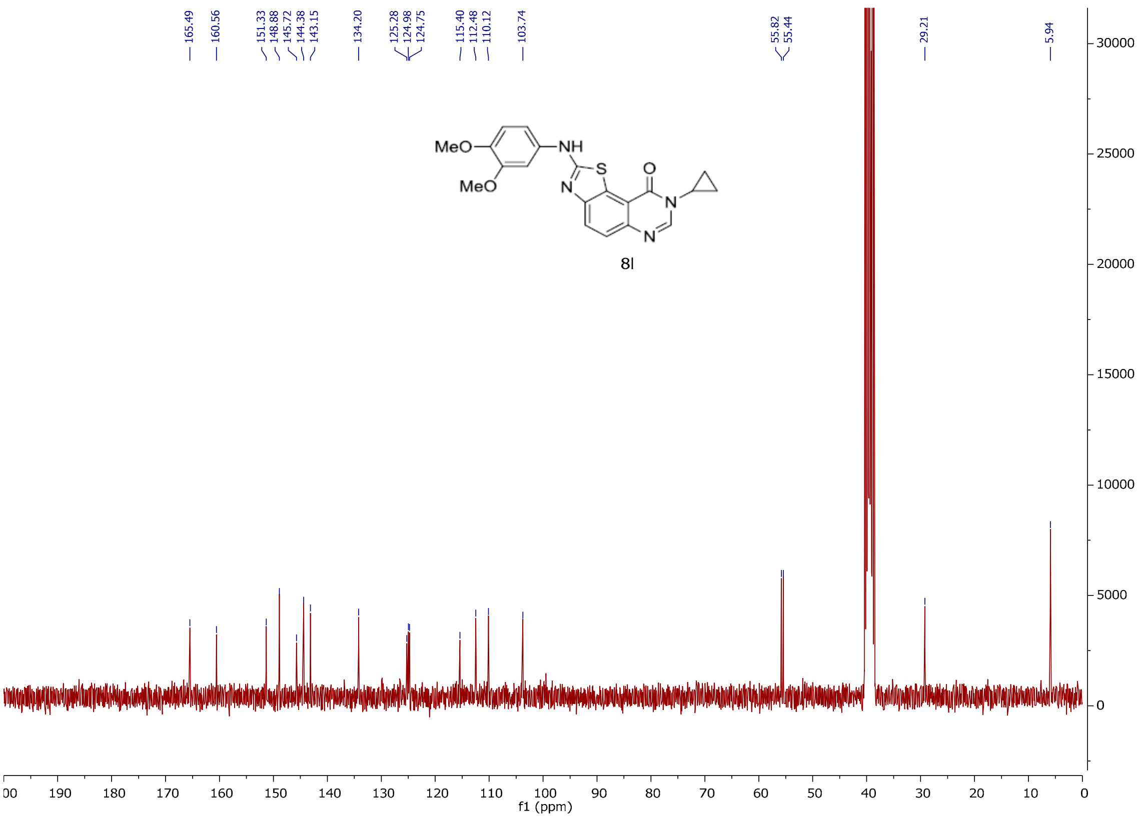

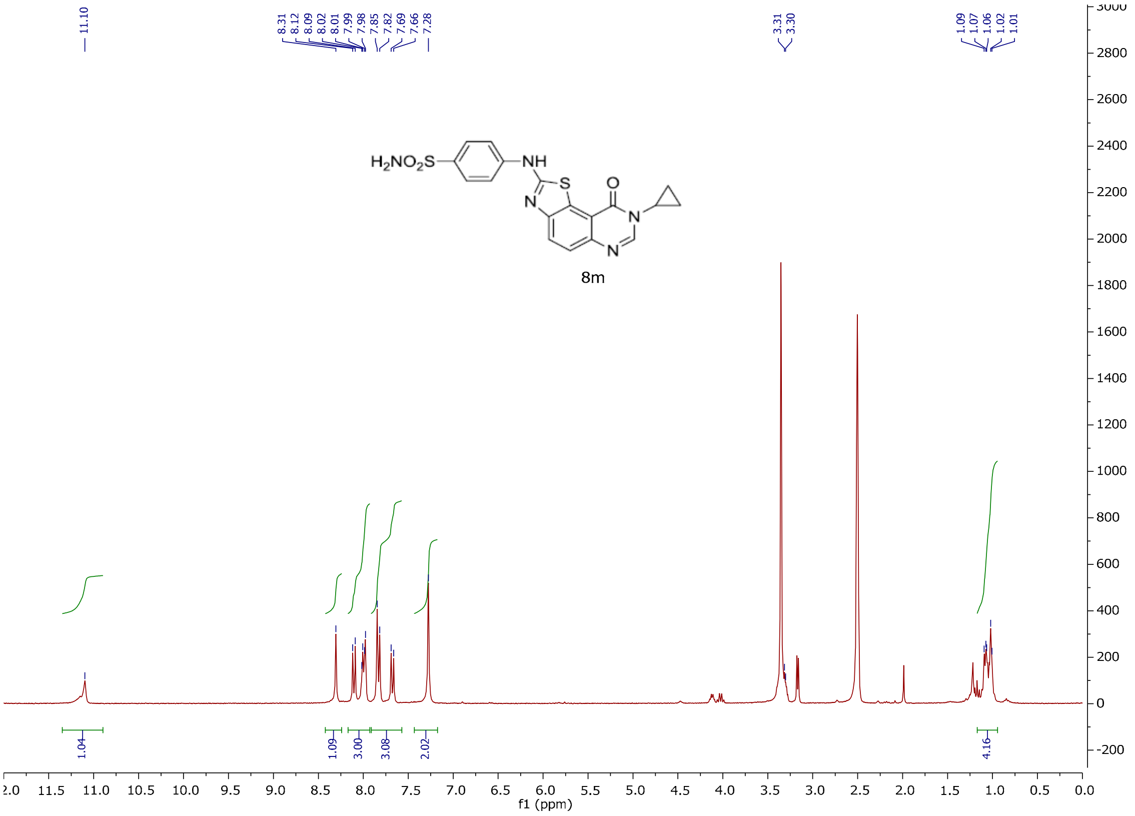

Figure S31. ^{1}H-NMR and ^{13}C-NMR Compound **8m**

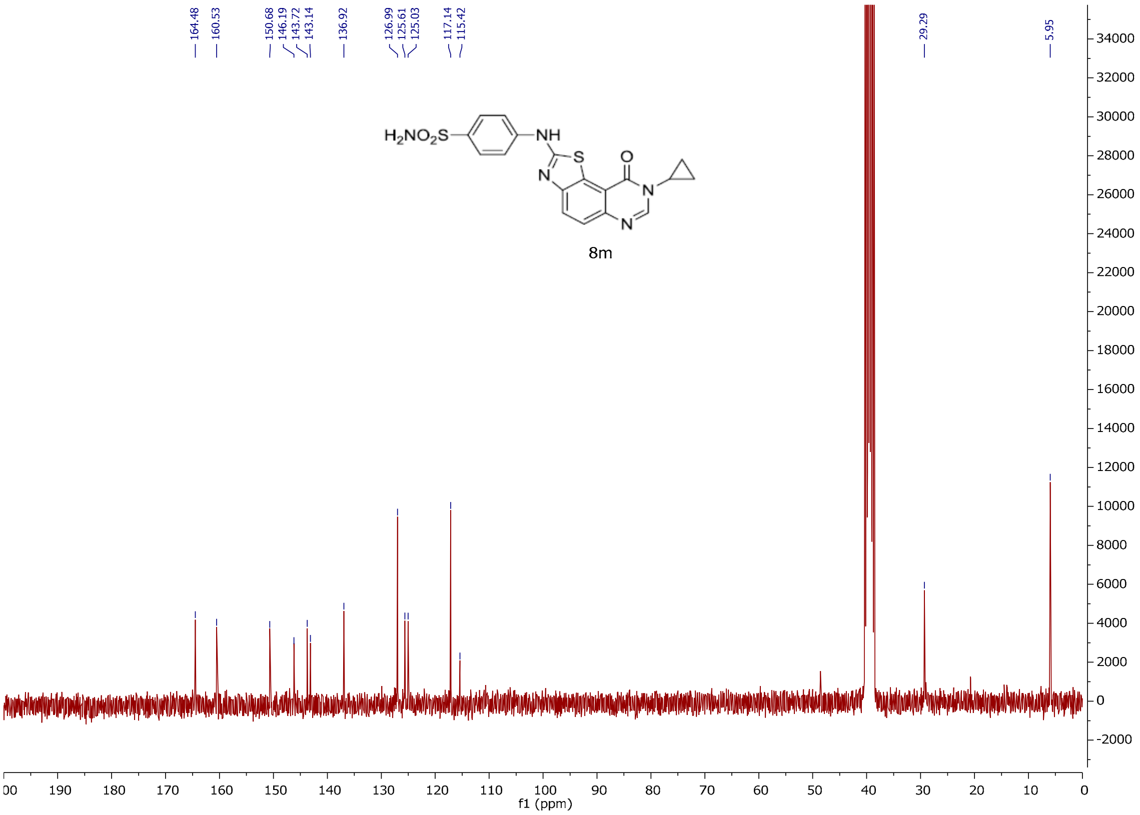

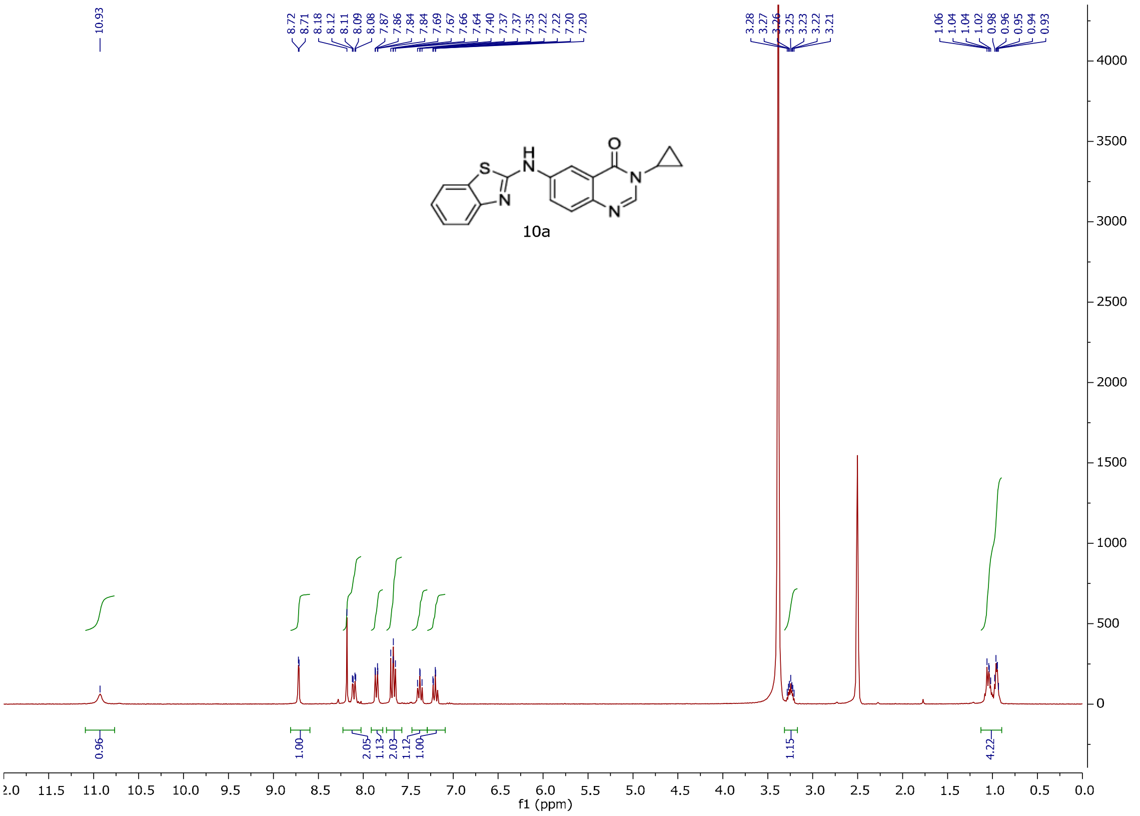

Figure S32. ^{1}H-NMR and ^{13}C-NMR Compound **10a**

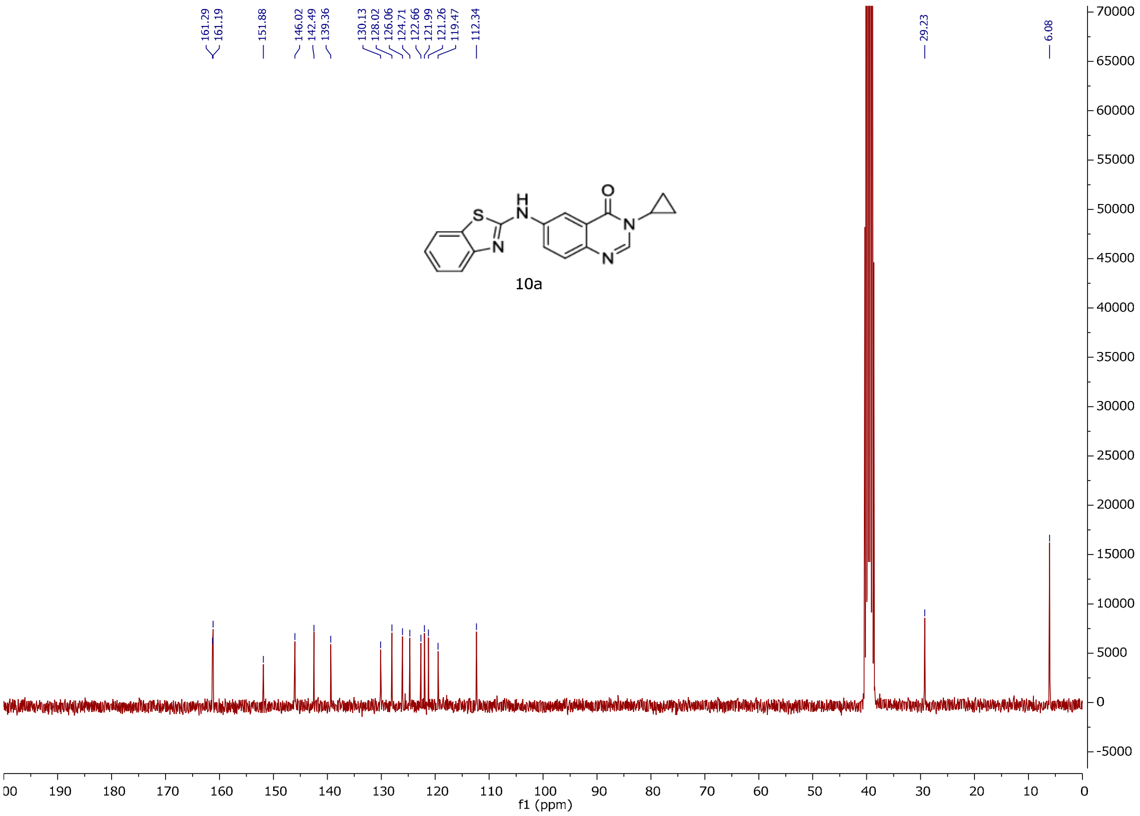

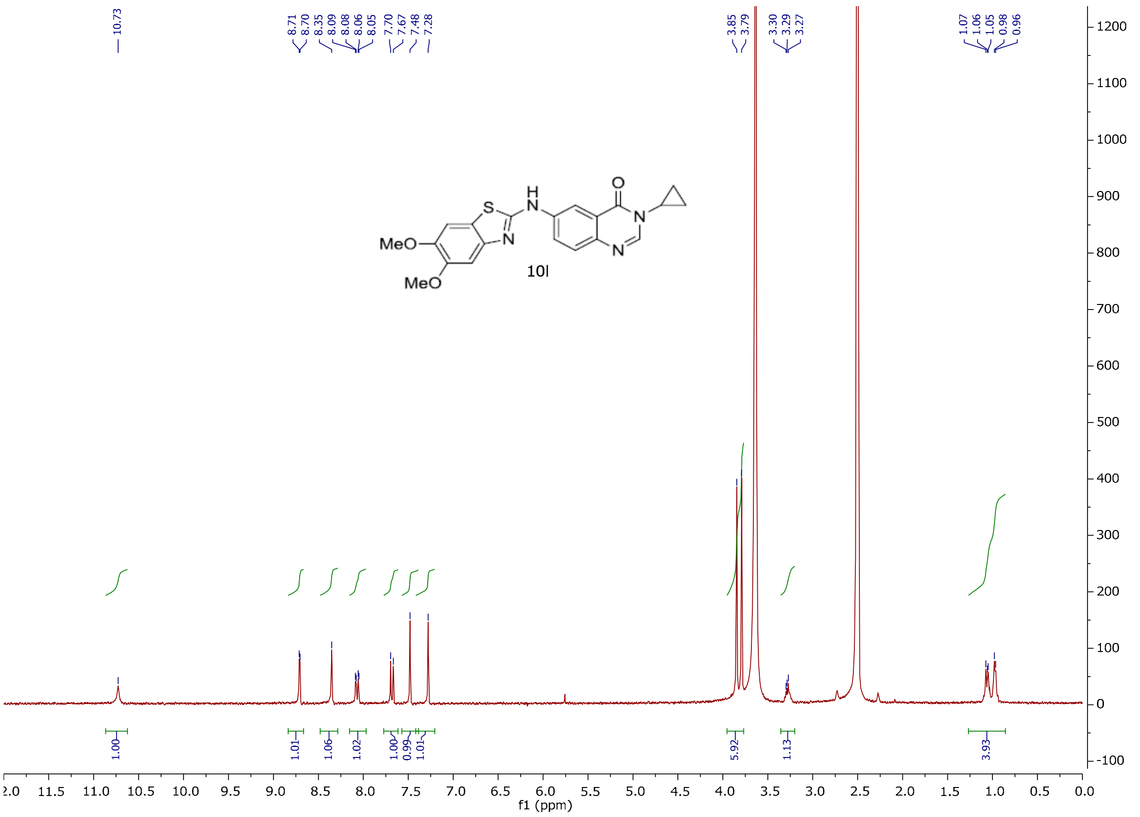

Figure S33. ^{1}H-NMR and ^{13}C-NMR Compound **10l**

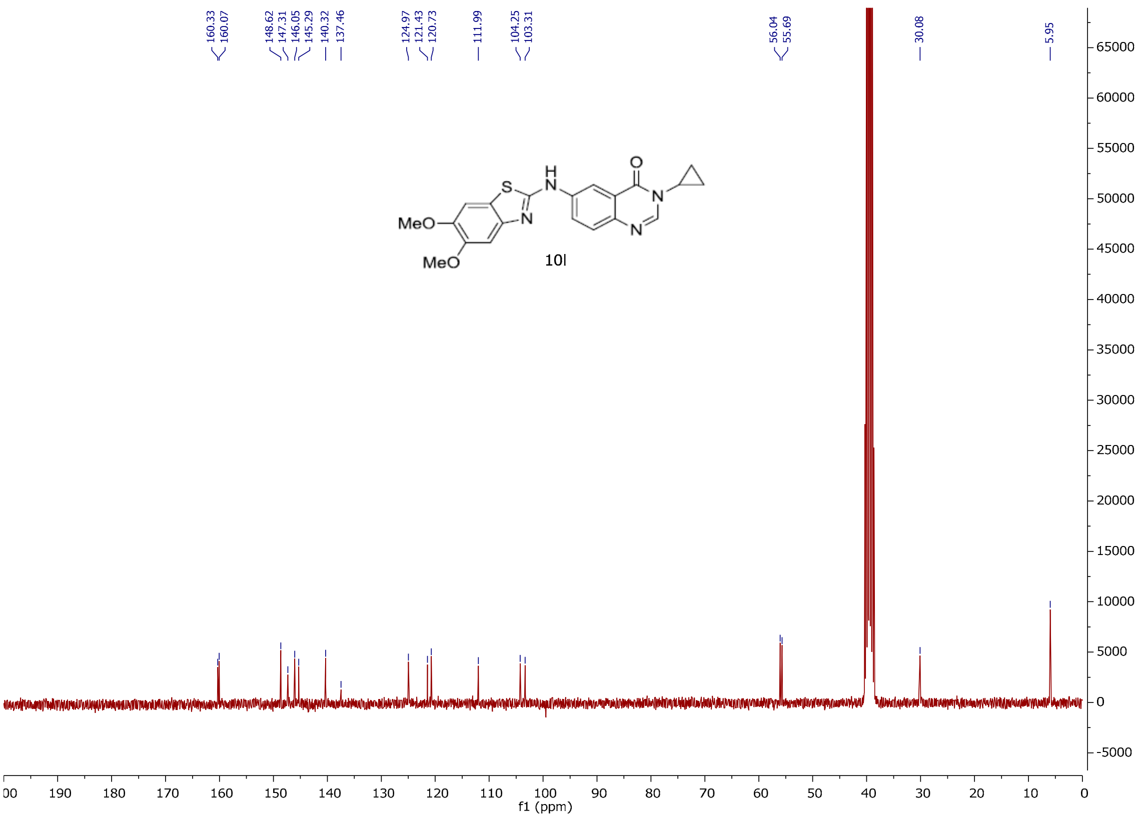

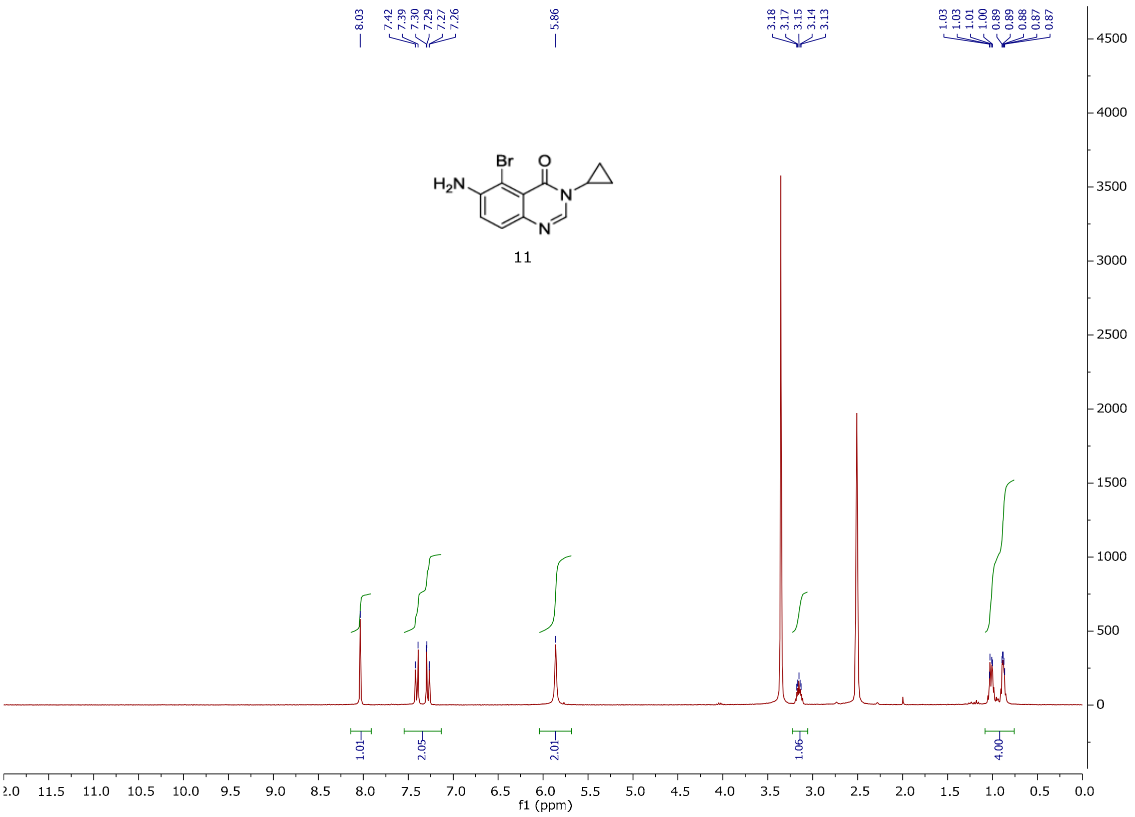

Figure S34. ^{1}H-NMR and ^{13}C-NMR Compound **11**

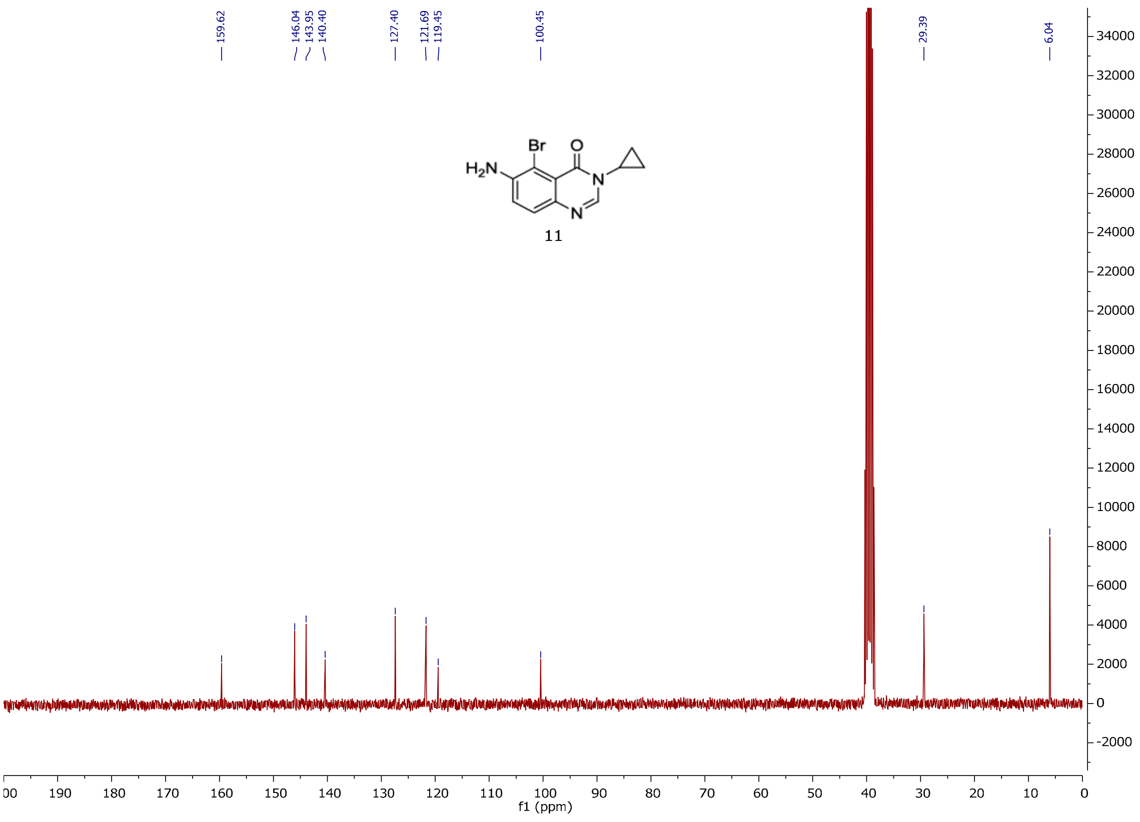

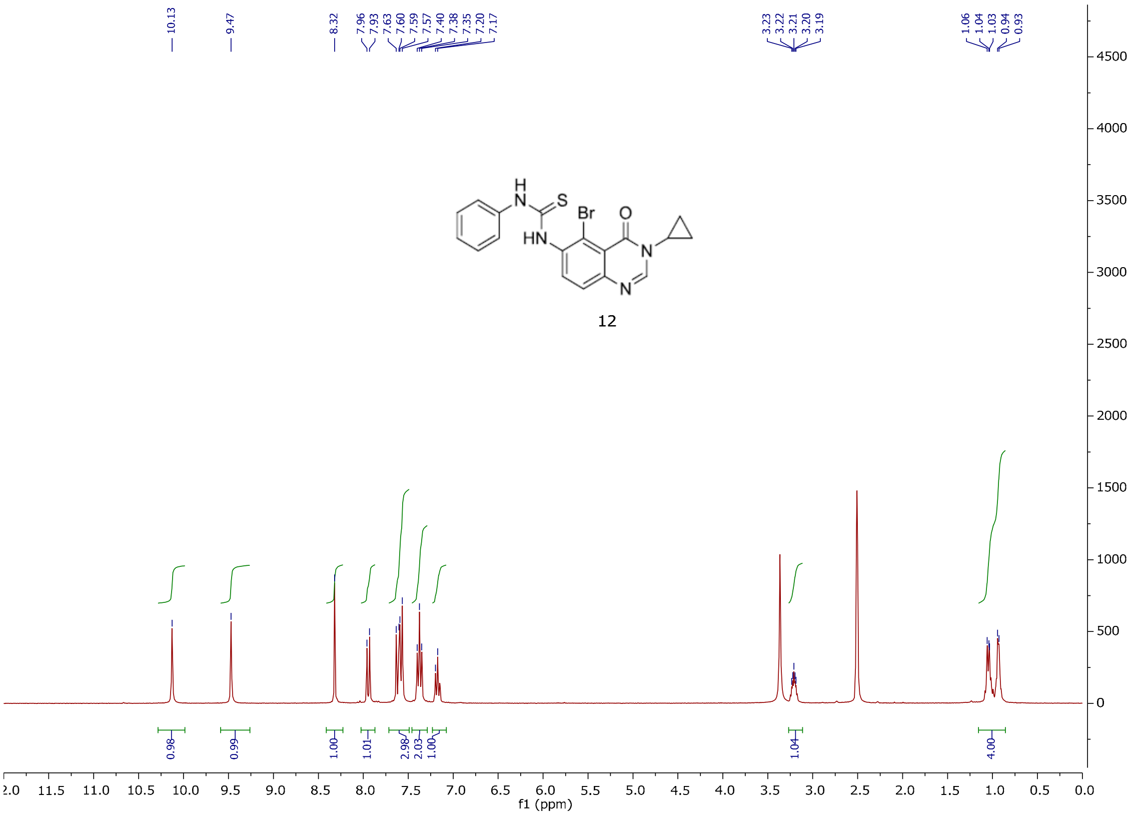

Figure S35. ^{1}H-NMR and ^{13}C-NMR Compound **12**

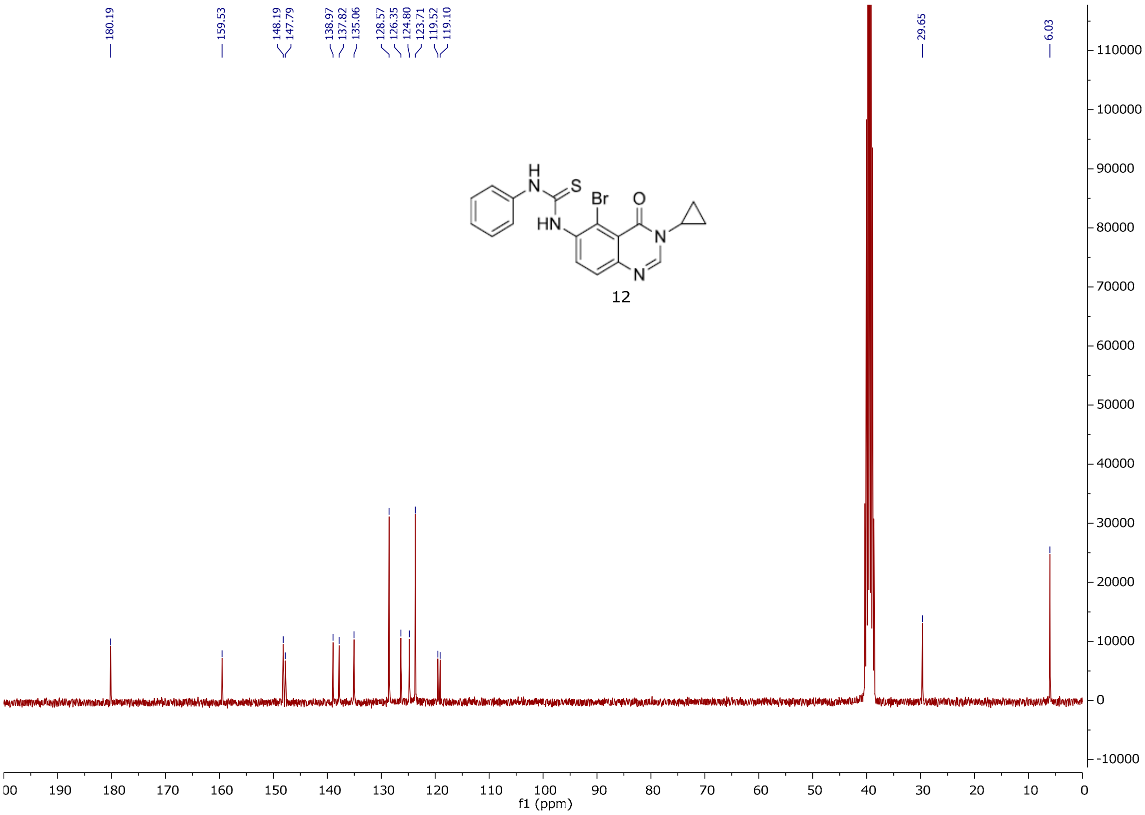

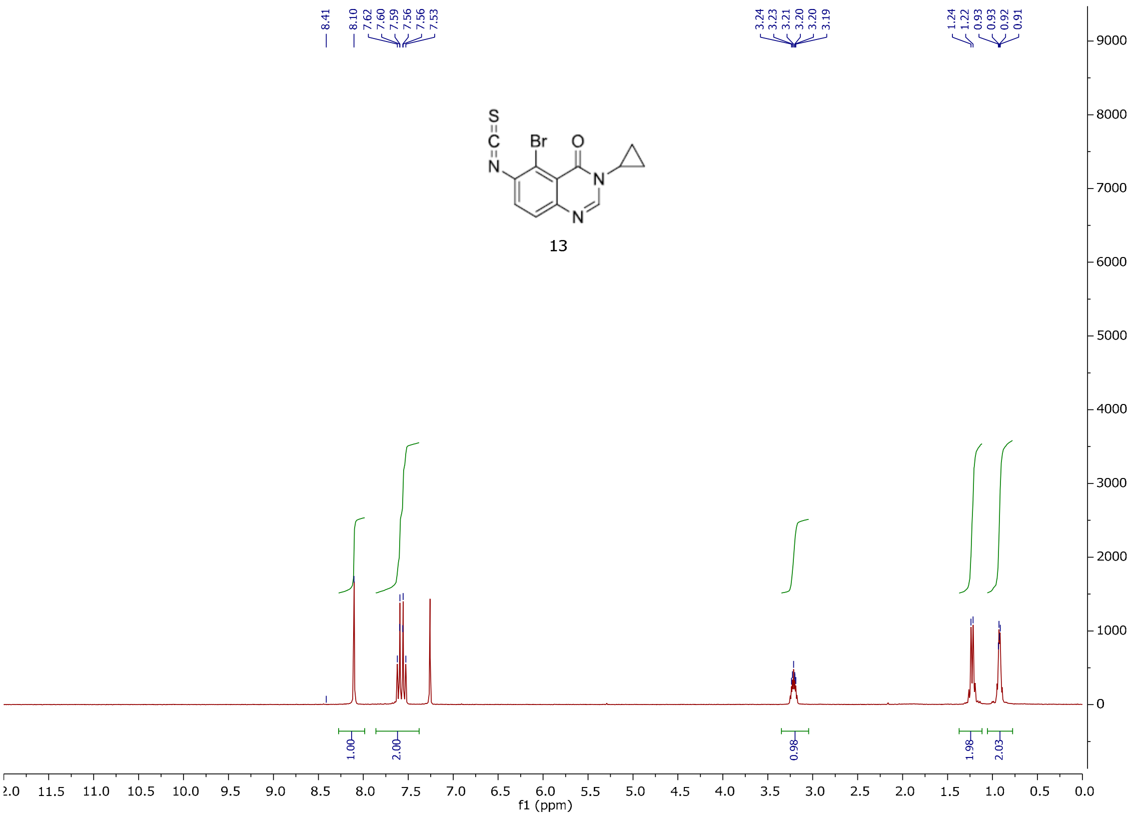

Figure S36. ^{1}H-NMR and ^{13}C-NMR Compound **13**

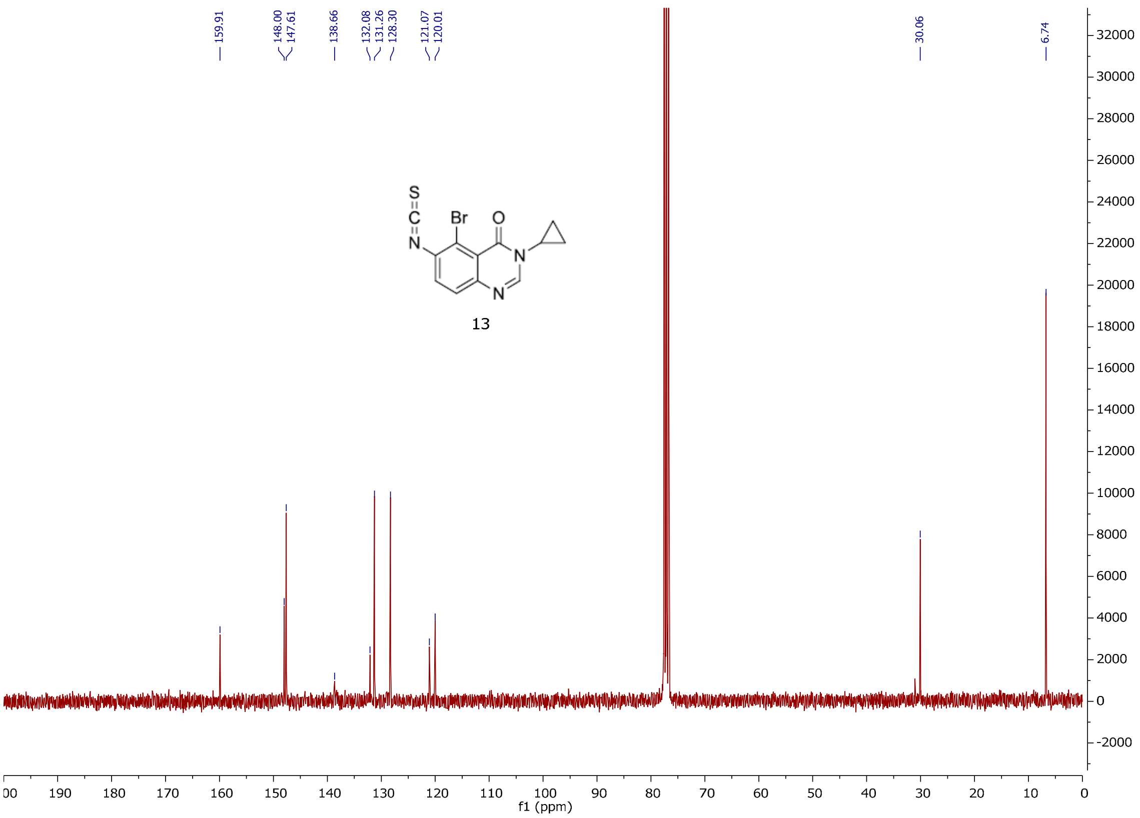

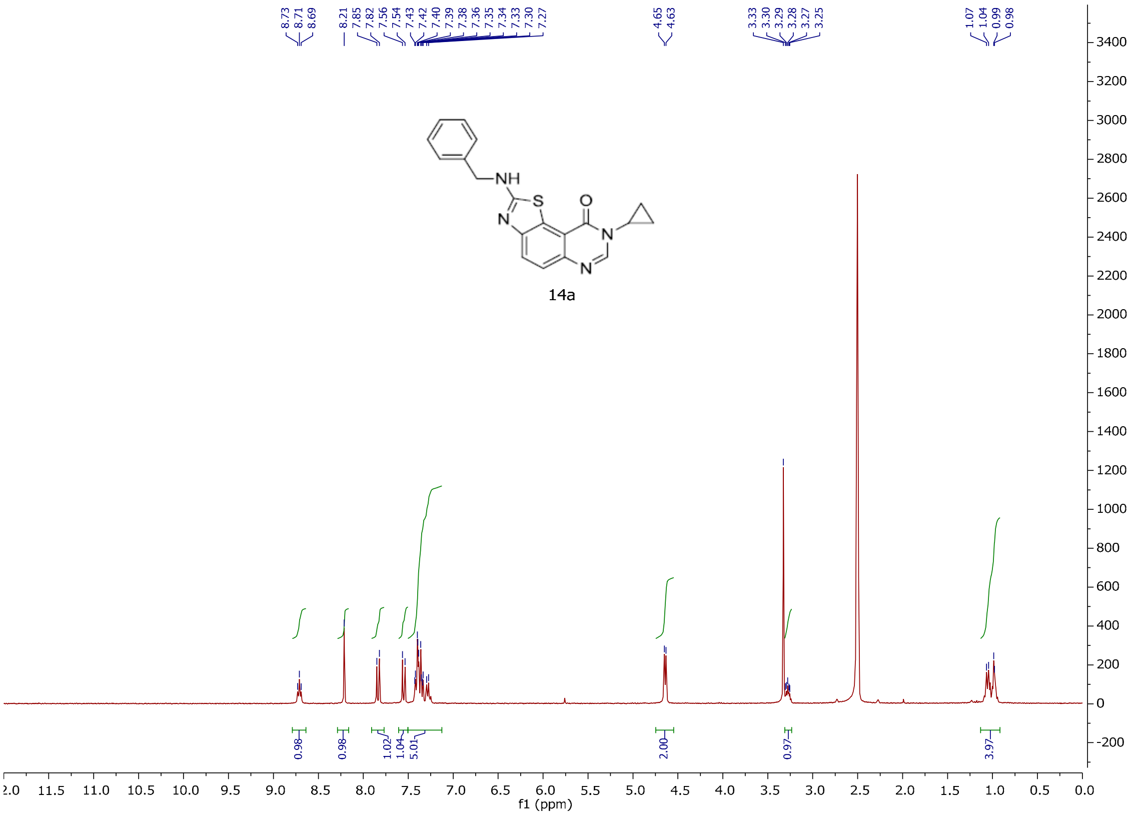

Figure S37. ^{1}H-NMR and ^{13}C-NMR Compound **14a**

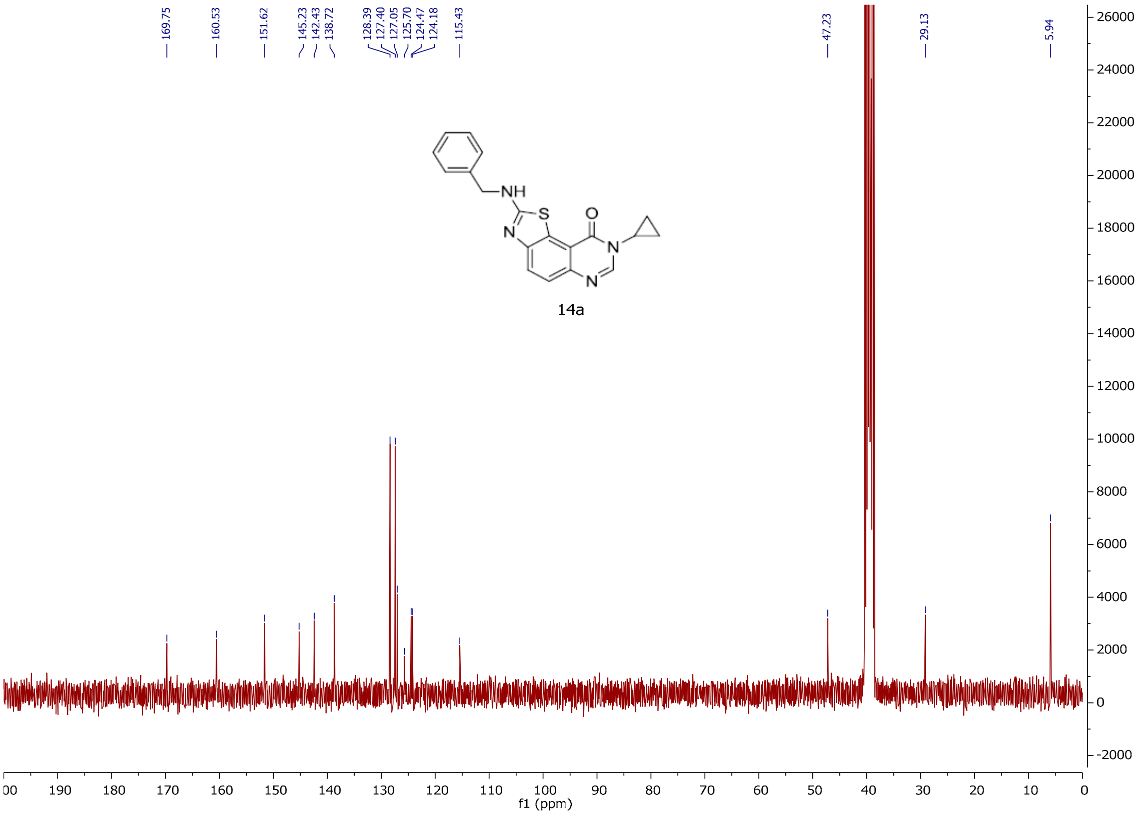

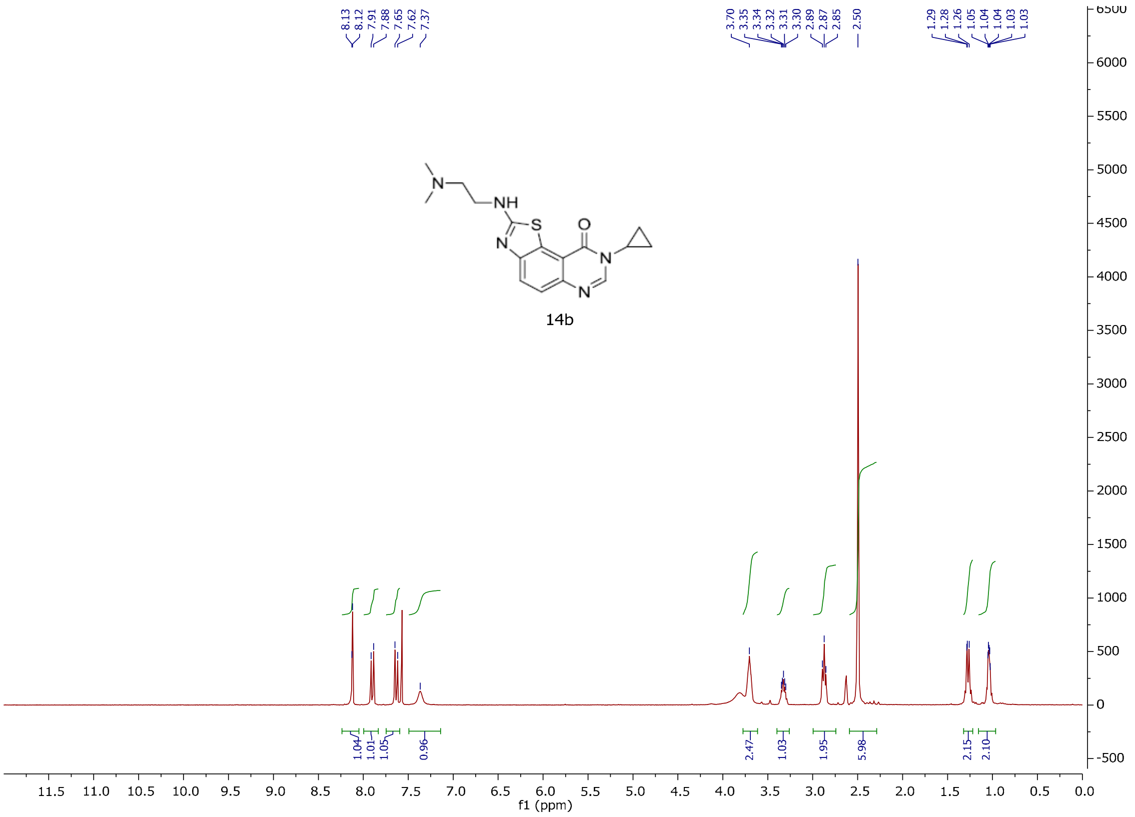

Figure S38. ^{1}H-NMR and ^{13}C-NMR Compound **14b**

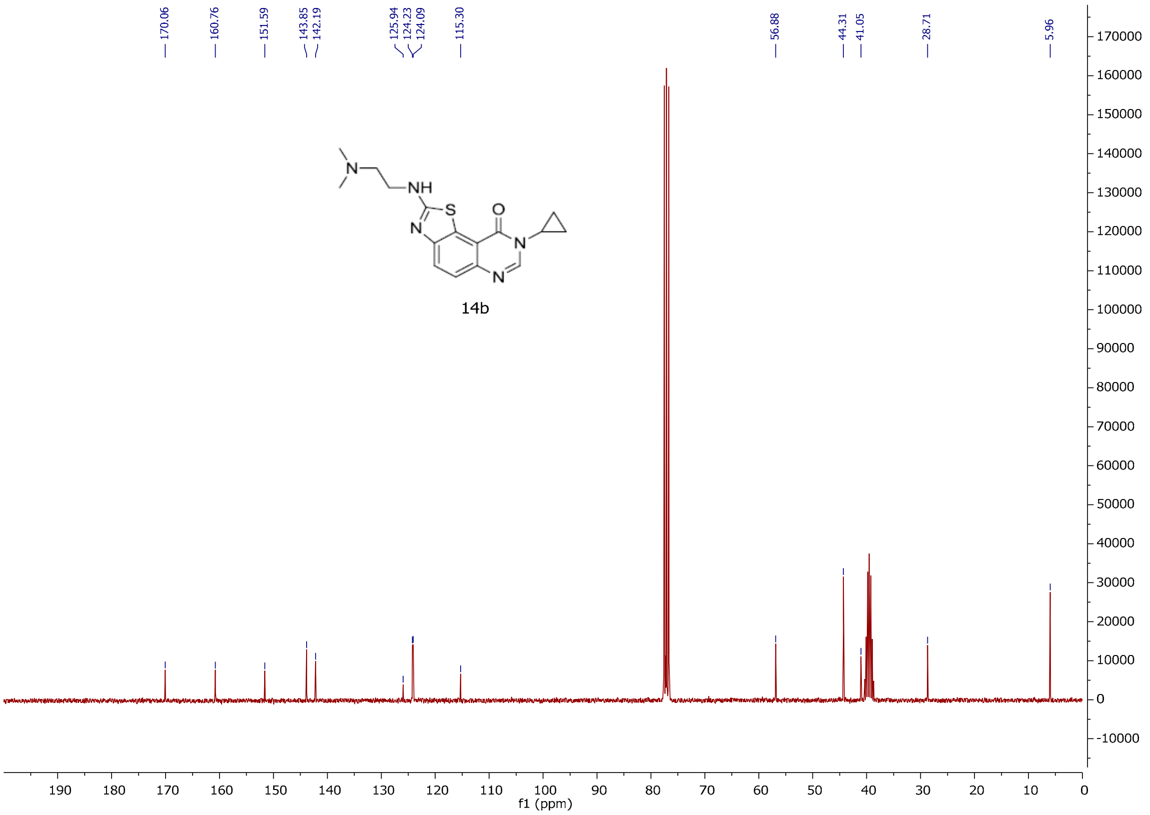

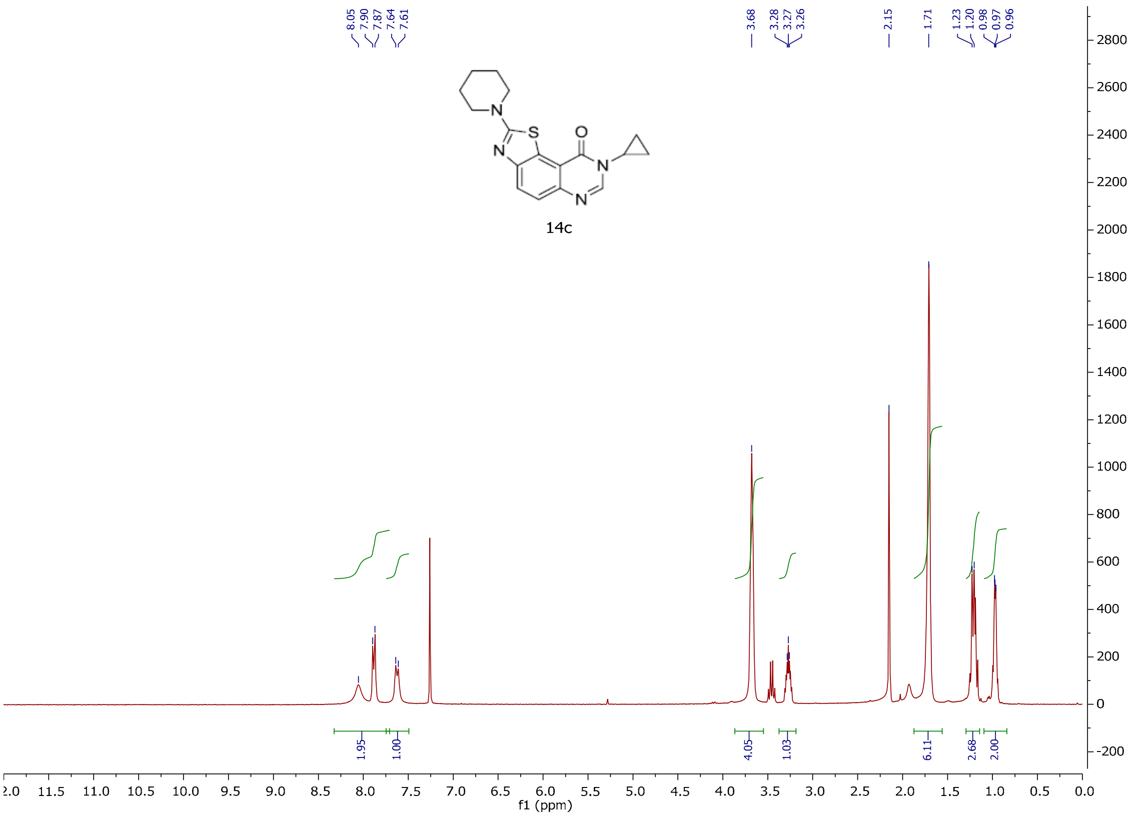

Figure S39. ^{1}H-NMR and ^{13}C-NMR Compound **14c**

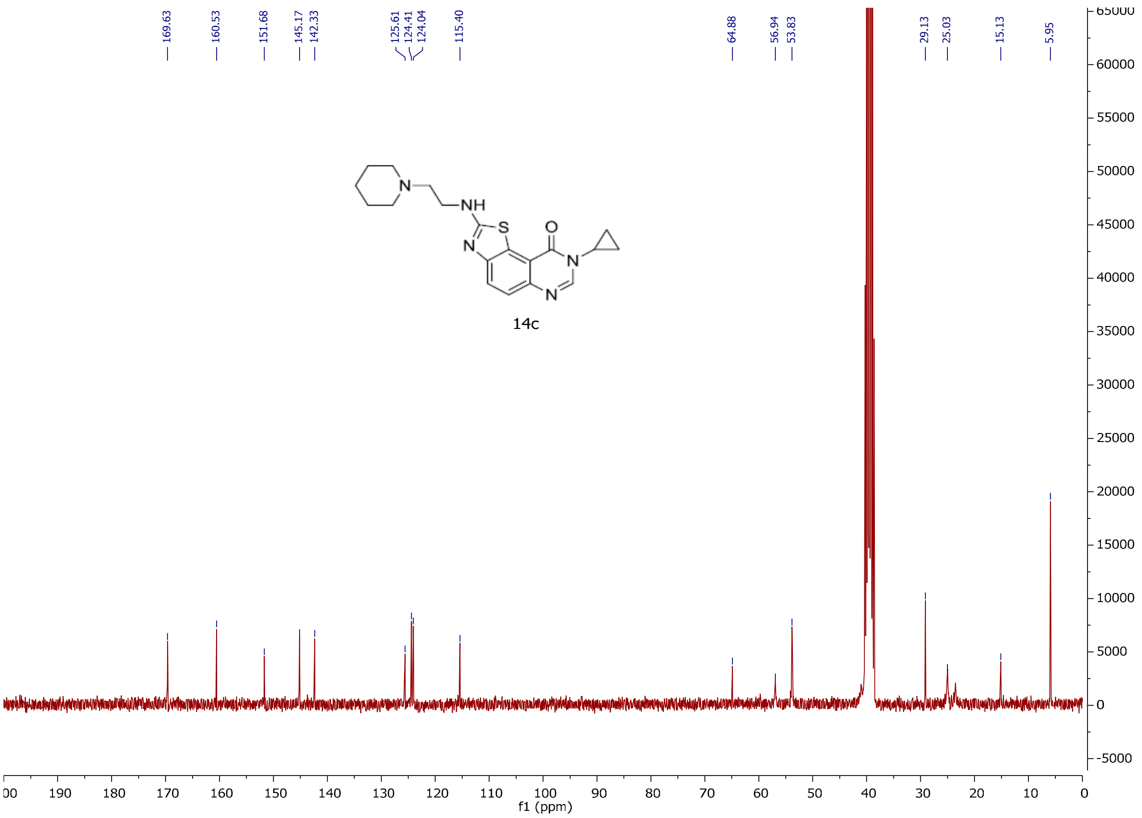

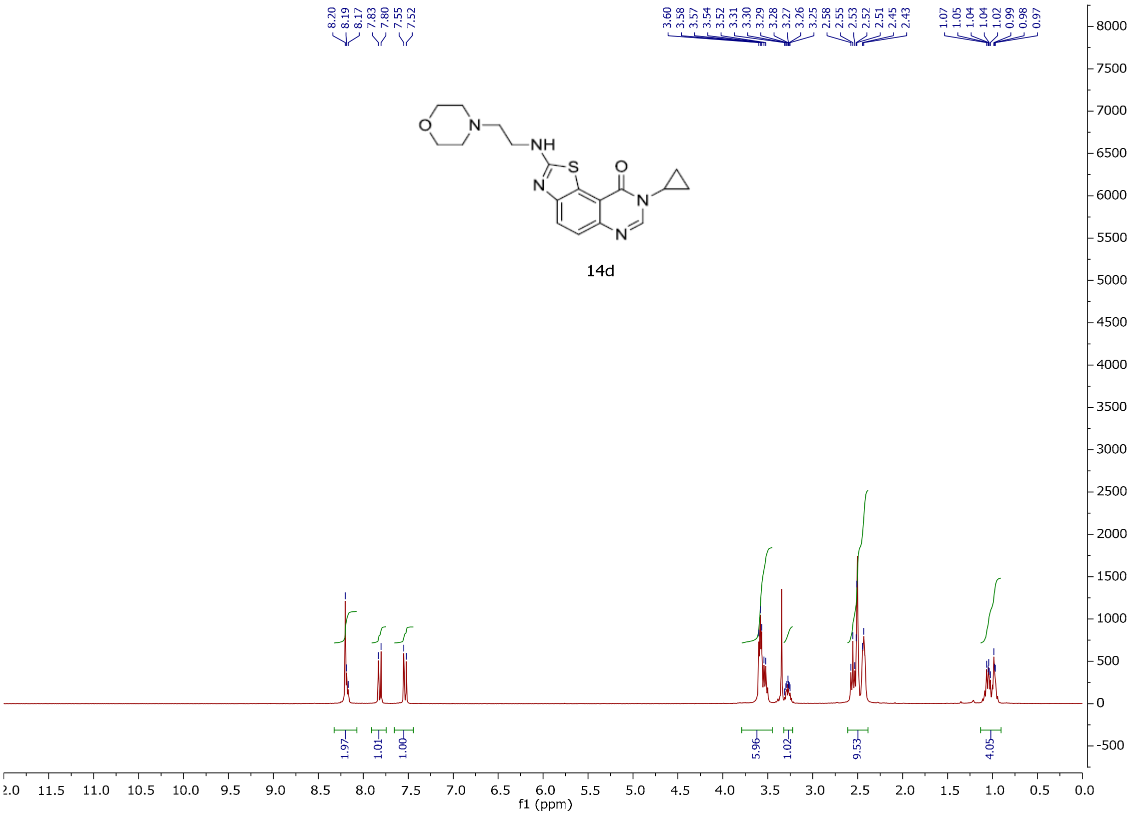

Figure S40. ^{1}H-NMR and ^{13}C-NMR Compound **14d**

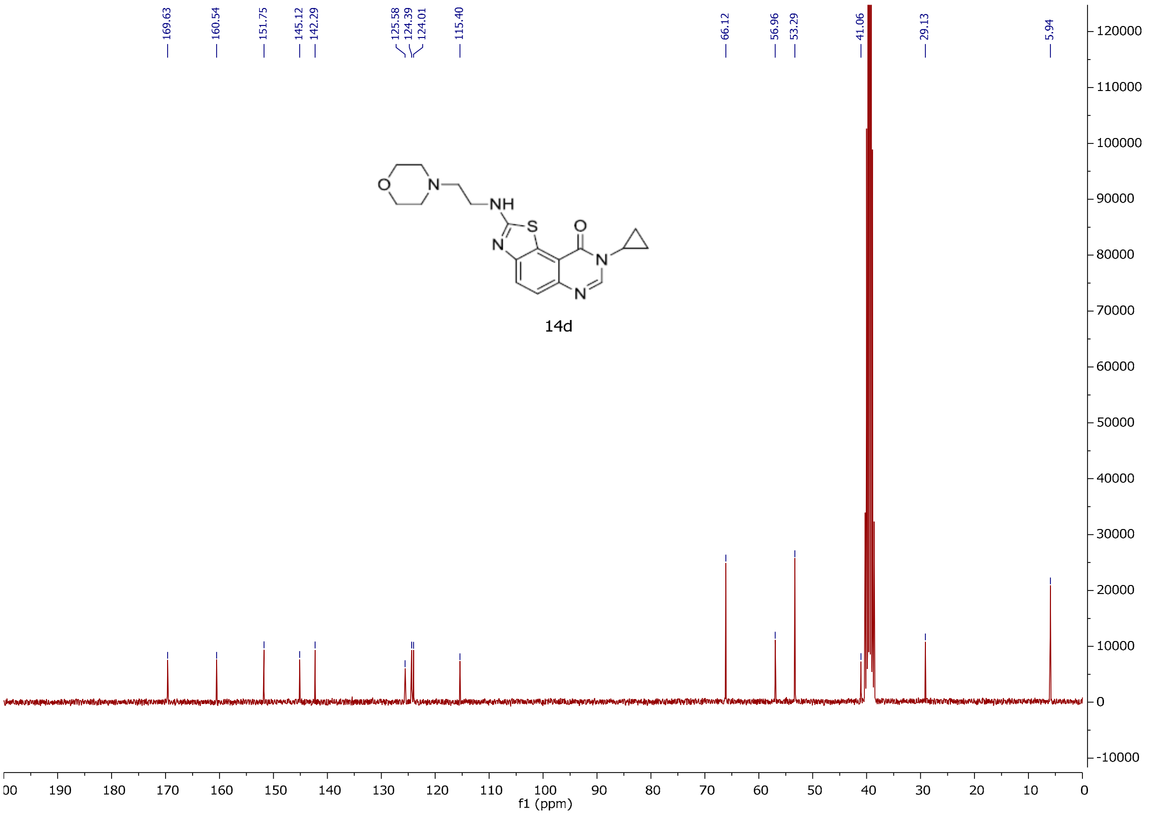

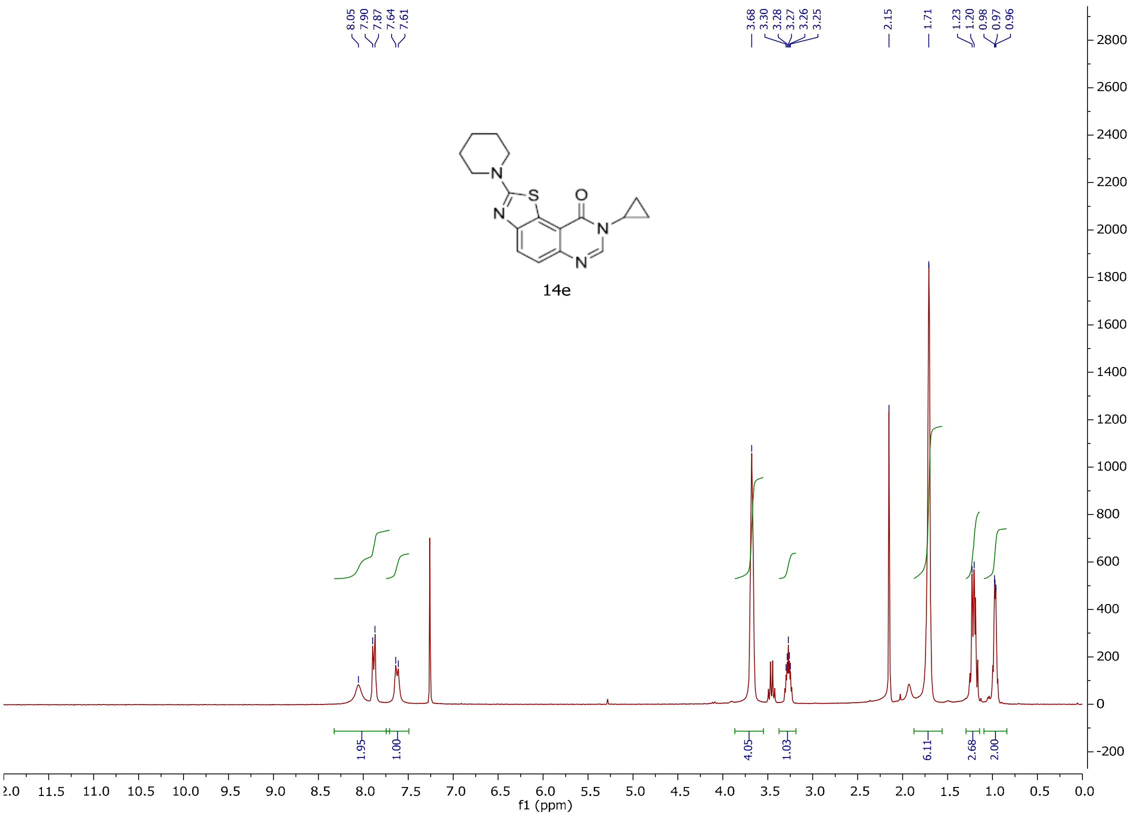

Figure S41. ^{1}H-NMR and ^{13}C-NMR Compound **14e**

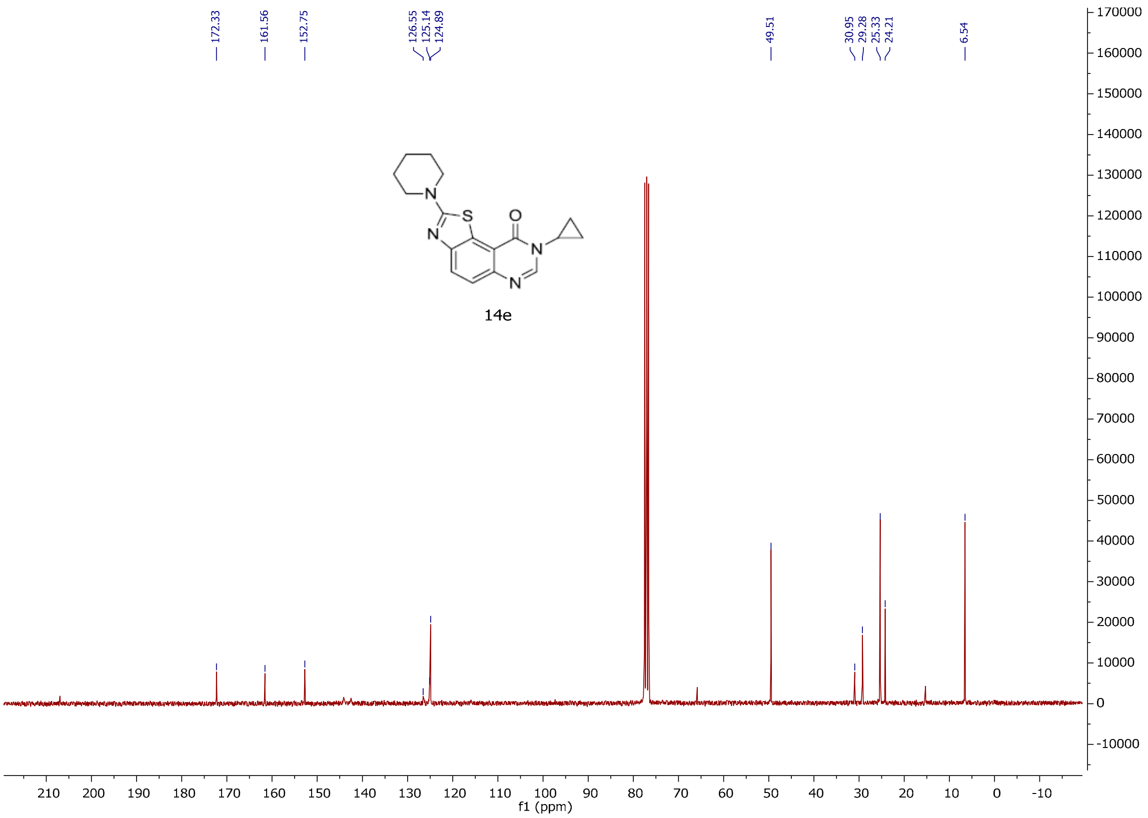

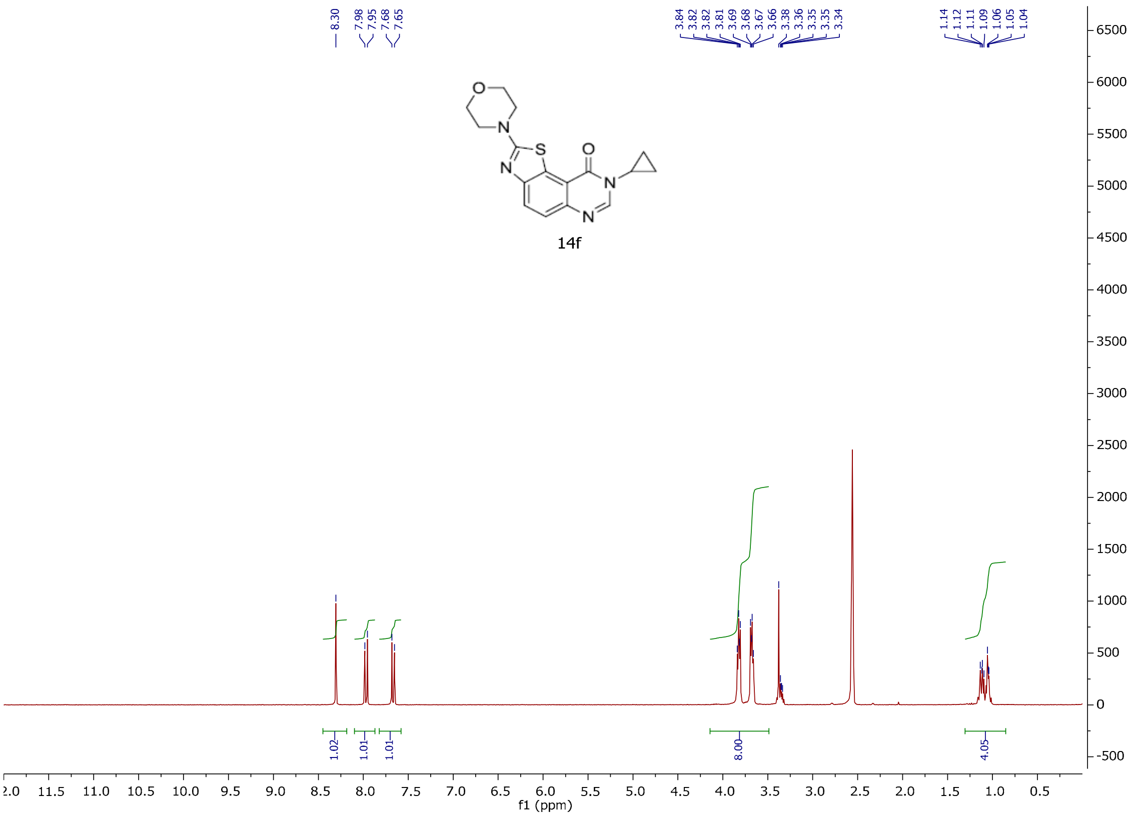

Figure S42. ^{1}H-NMR and ^{13}C-NMR Compound **14f**

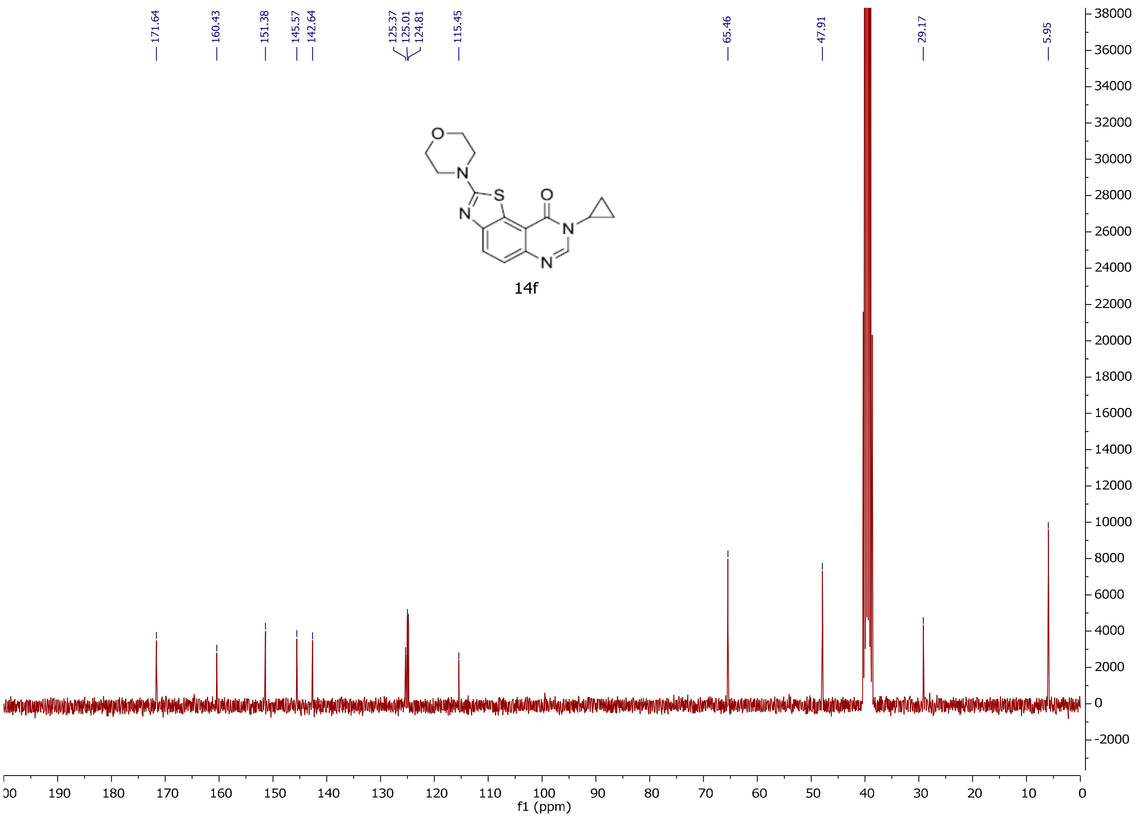

Figure S43. ^{1}H-NMR and ^{13}C-NMR Compound **15**

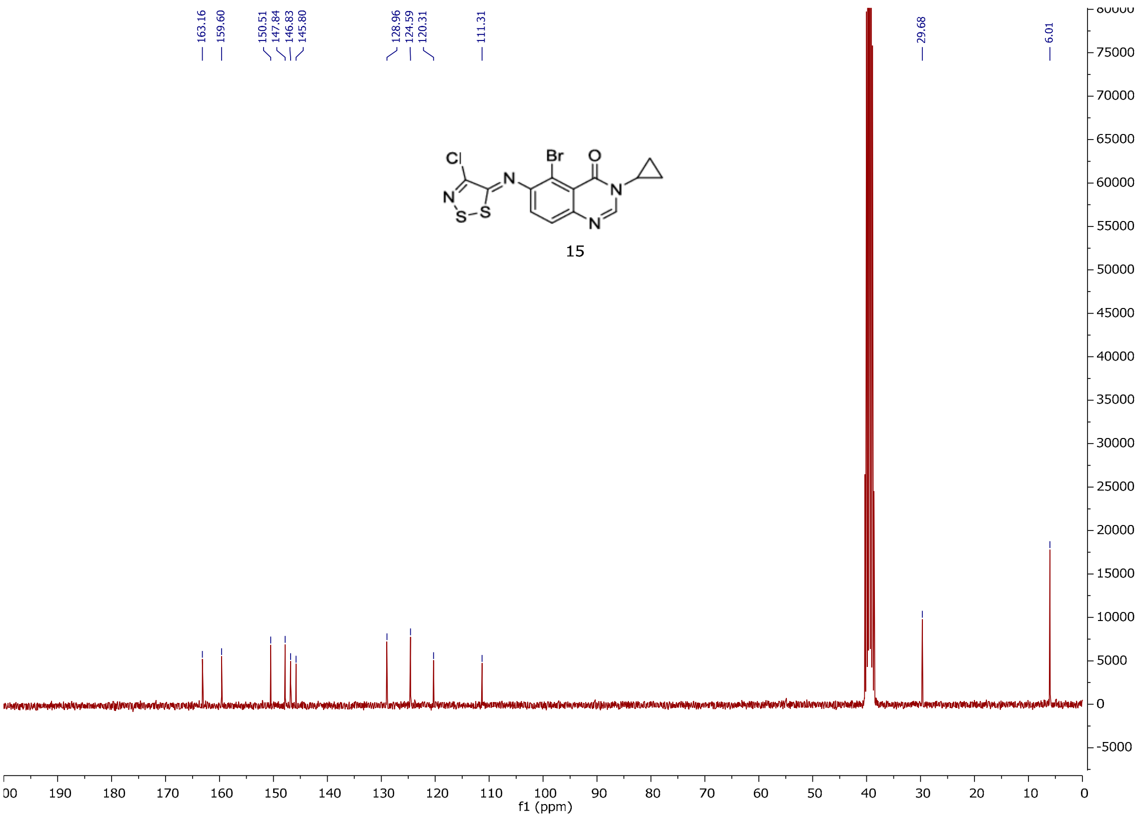

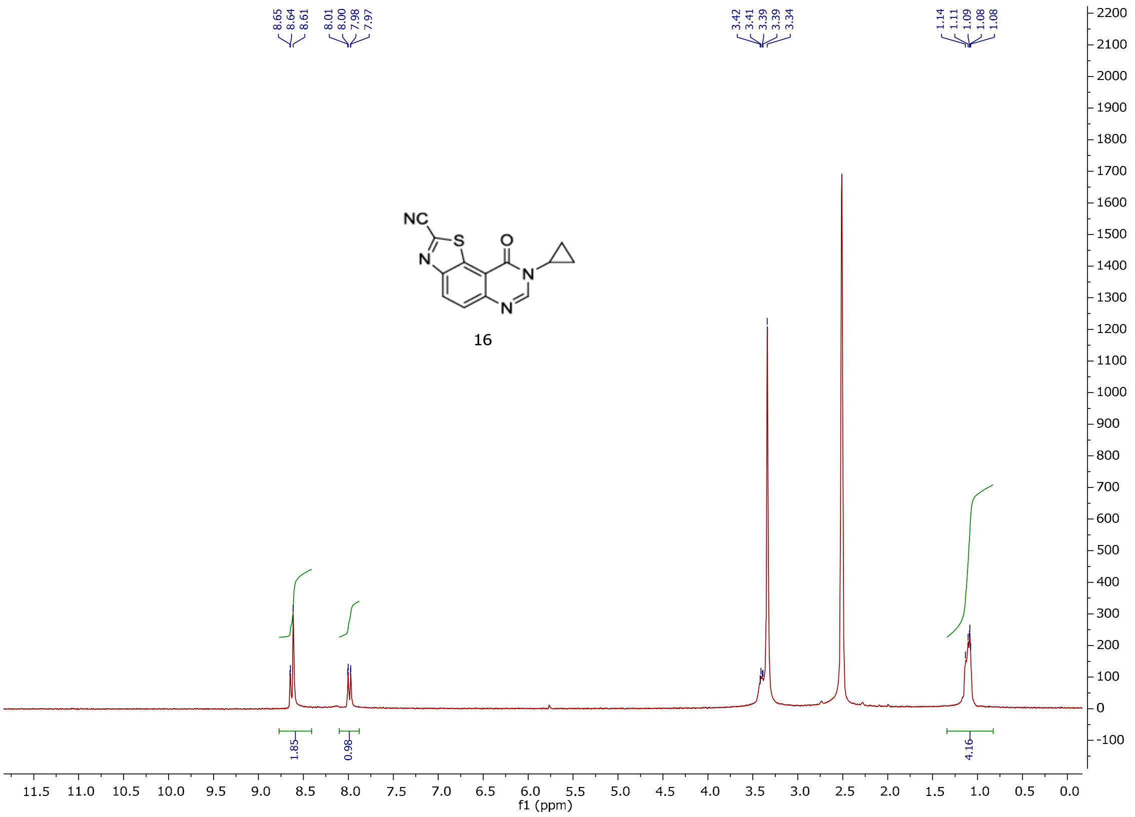

Figure S44. ^{1}H-NMR and ^{13}C-NMR Compound **16**

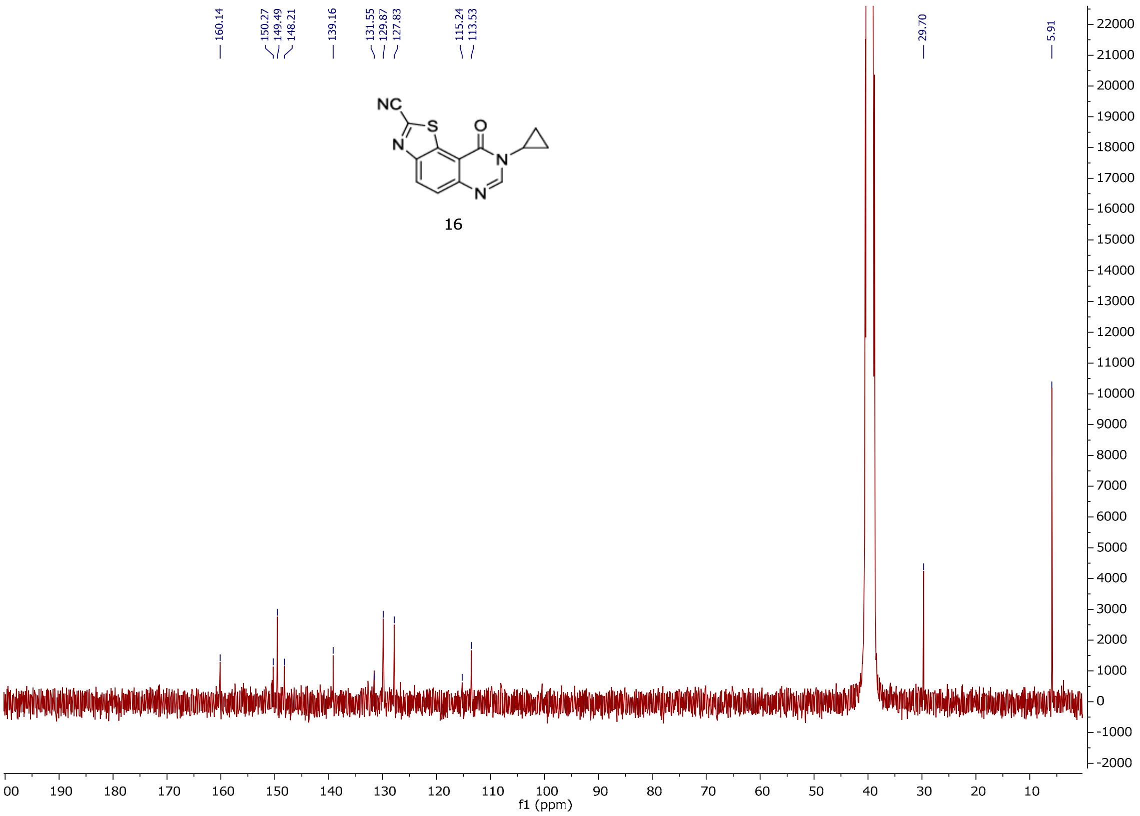

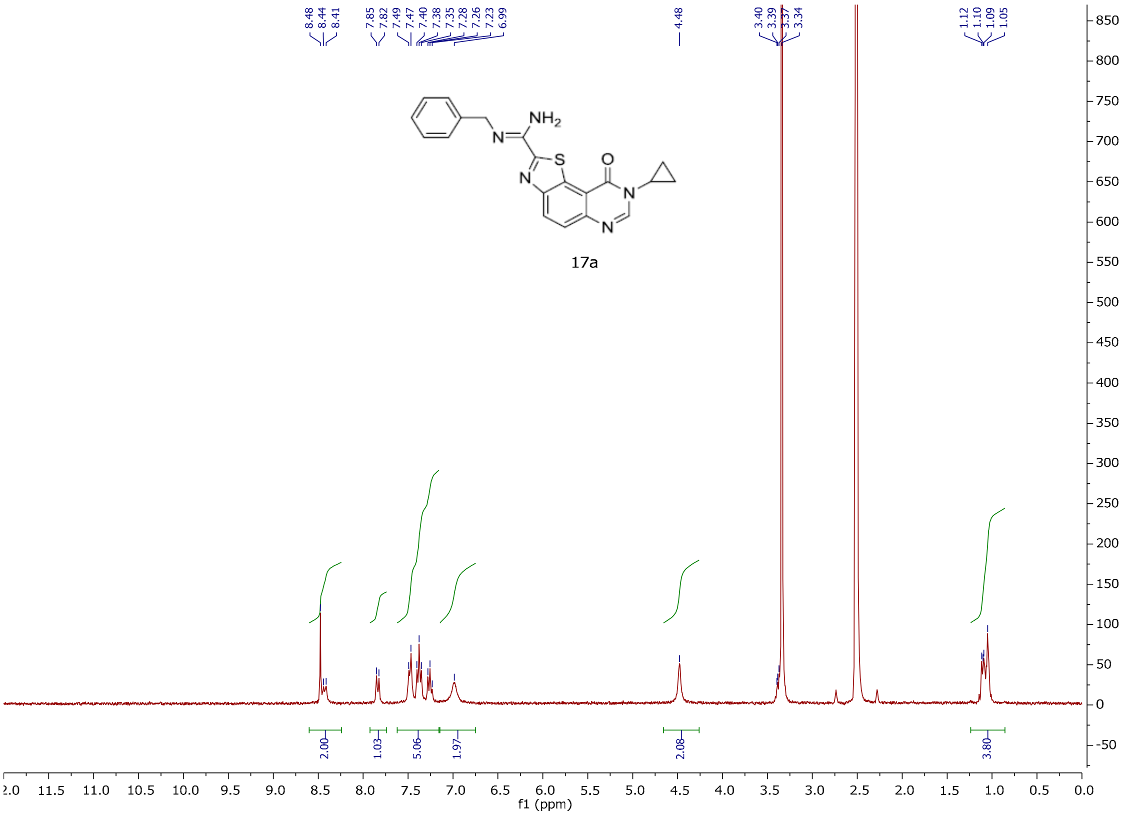

Figure S45. ^{1}H-NMR and ^{13}C-NMR Compound **17a**

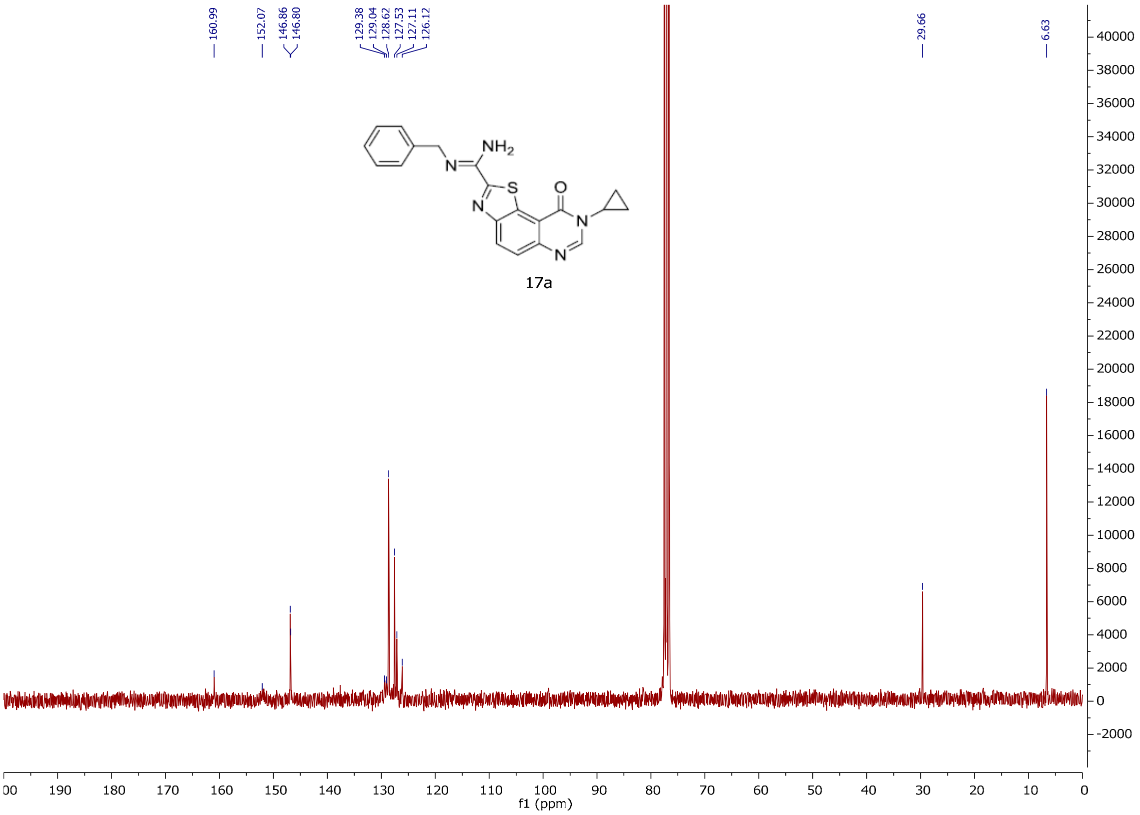

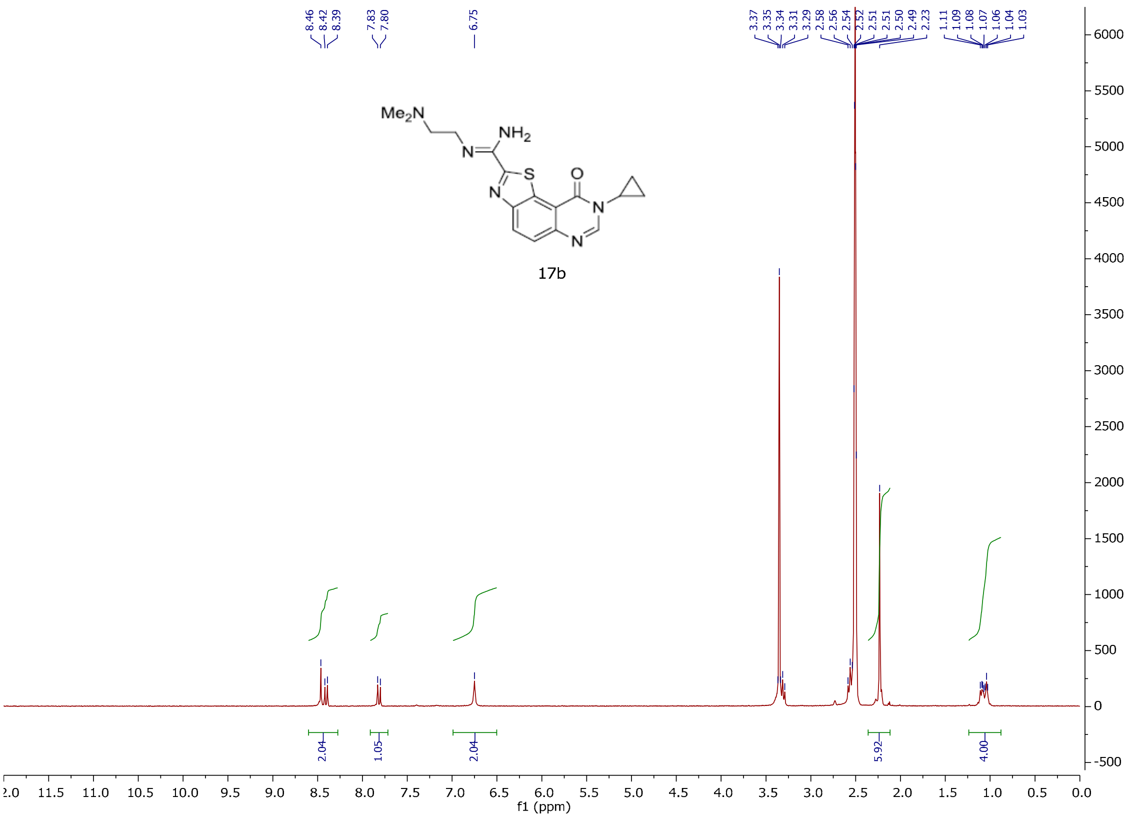

Figure S46. ^{1}H-NMR and ^{13}C-NMR Compound **17b**

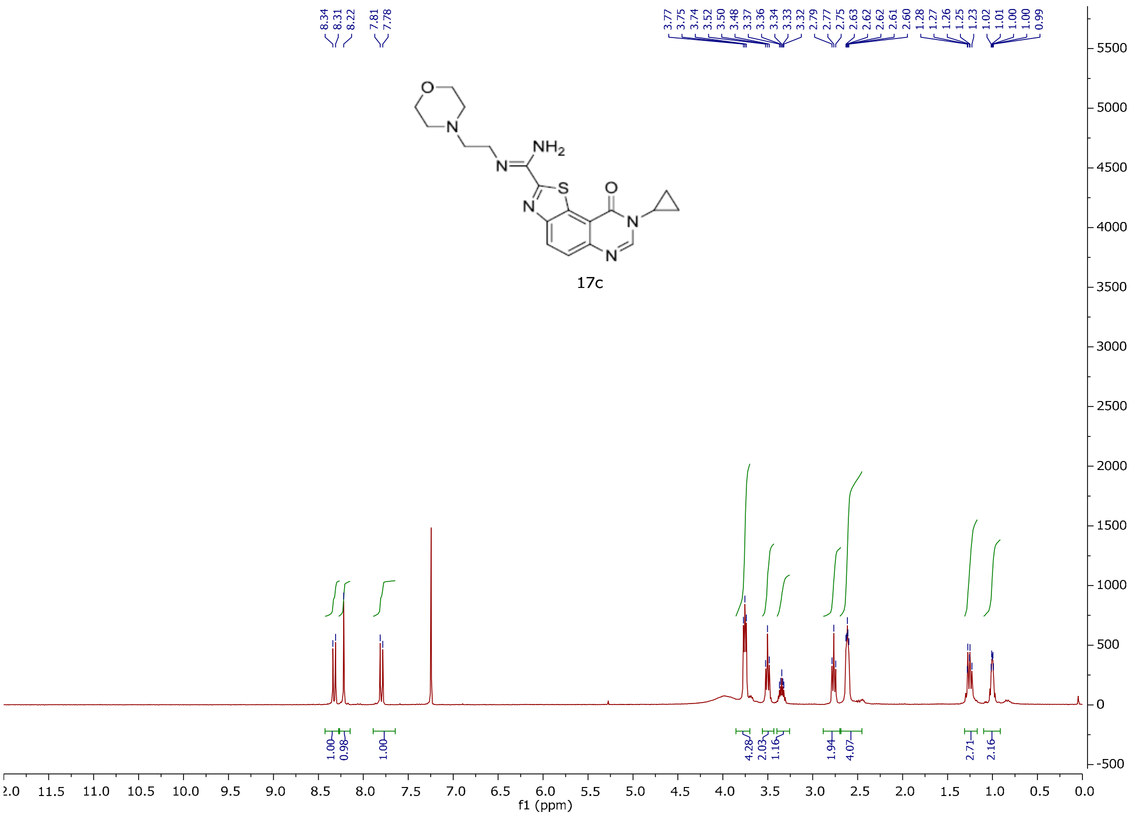

Figure S47. ^{1}H-NMR and ^{13}C-NMR Compound **17c**

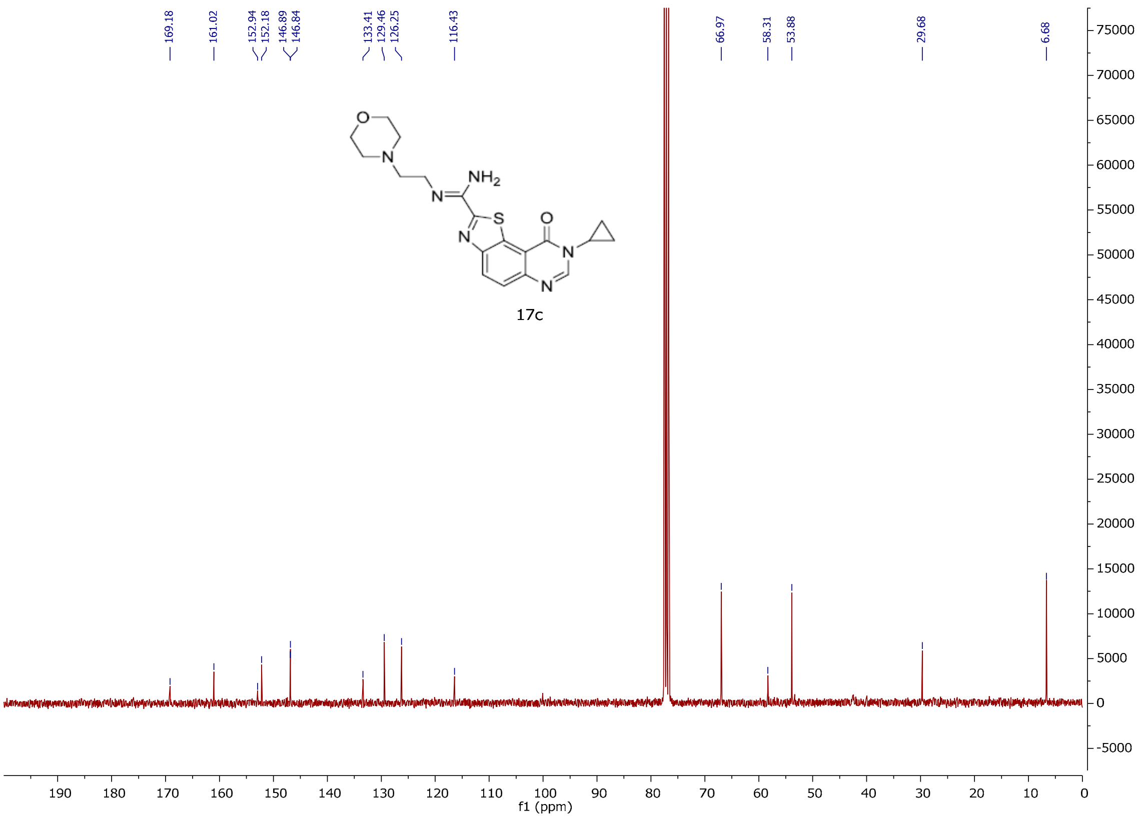

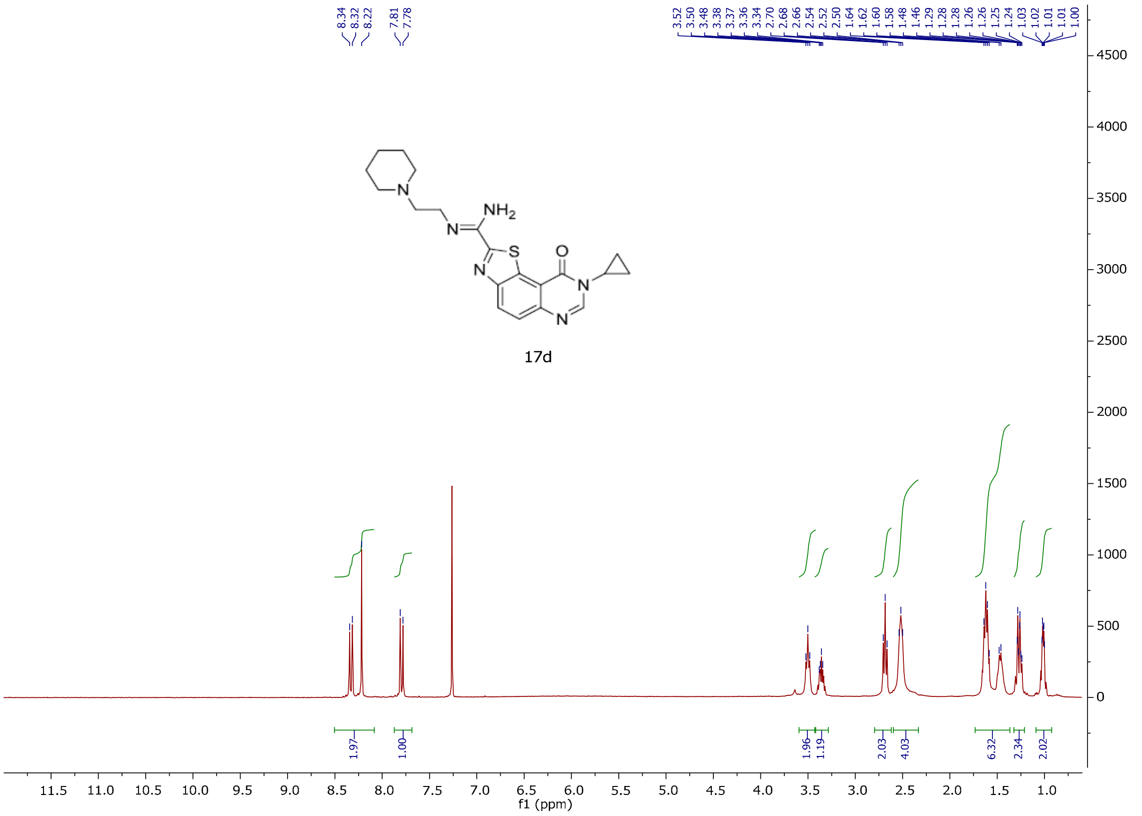

Figure S48. ^{1}H-NMR and ^{13}C-NMR Compound 1**7d**

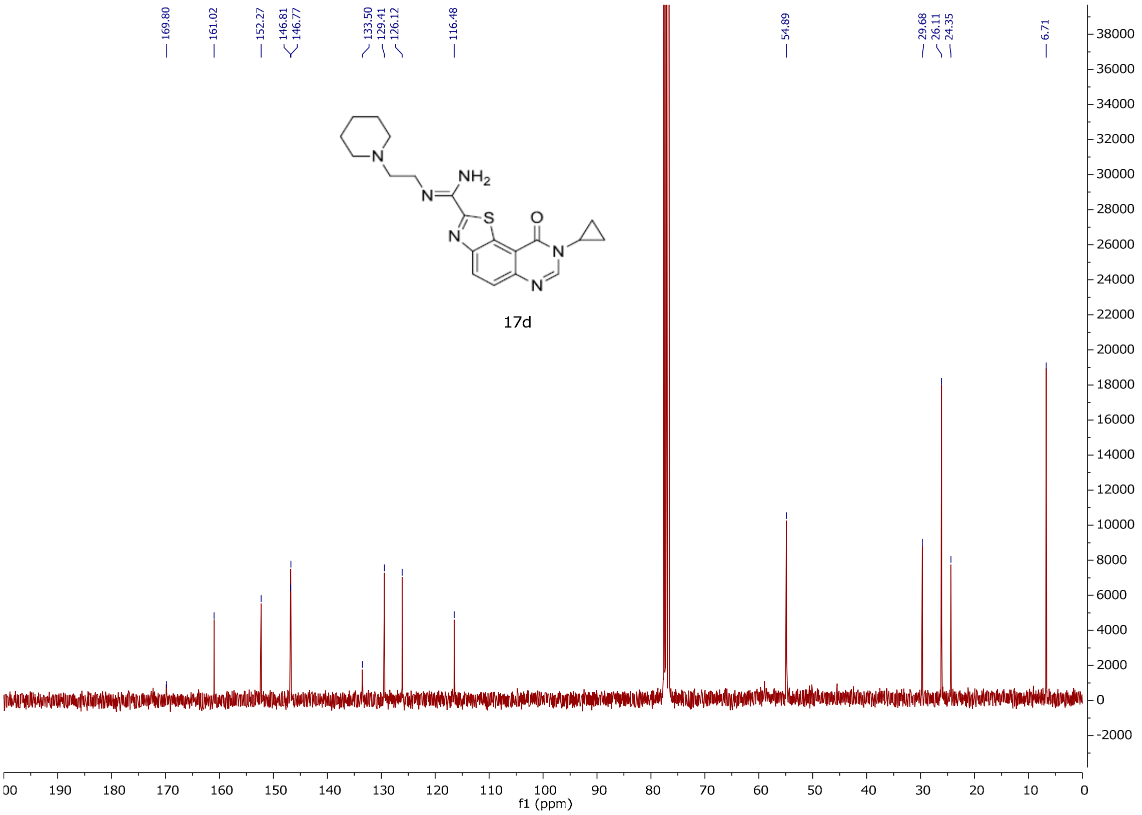

Supplement: Supplementary file 1 [file molecules-21-00794-s001.pdf]
